# A Unique Class of Cyclases with a Kinase Fold Catalyzes Enethiol-Mediated Macrocyclization of Aminovinyl-Cysteine Motifs in Lanthipeptides

**DOI:** 10.1021/acscentsci.5c00569

**Published:** 2025-06-18

**Authors:** Xiang-Qian Xie, Wen Guo, Yin-Zheng Xia, Li-Juan Liao, Meng-Xin Sun, Jing-Xue Wang, Jiang-Tao Gao, Hong-Wei Yao, Huan Wang

**Affiliations:** † State Key Laboratory of Coordination Chemistry, Chemistry and Biomedicine Innovation Center of Nanjing University, Jiangsu Key Laboratory of Advanced Organic Materials, School of Chemistry and Chemical Engineering, 12581 Nanjing University, Nanjing 210093, China; ‡ Institute of Molecular Enzymology, School of Life Sciences, Suzhou Medical College of Soochow University, 12582 Soochow University, Suzhou 215123, China; § State Key Laboratory of Ecological Pest Control for Fujian and Taiwan Crops, College of Life Sciences, 12449 Fujian Agriculture and Forestry University, 350002 Fuzhou, China

## Abstract

2-Aminovinyl-cysteine (AviCys) motifs represent a unique class of macrocyclic structures found in many ribosomally synthesized and post-translationally modified peptides (RiPPs). Despite their essential role in bioactivity, their biosynthetic machinery, particularly the cyclases catalyzing Avi­(Me)­Cys macrocyclization, has not been fully characterized. Herein, we report the discovery and biosynthetic elucidation of class V lanthipeptides rosin A1–A3, which feature a lanthionine (Lan) macrocycle and a C-terminal 2-aminovinyl-3-methyl-cysteine (AviMeCys) macrocycle. Rosins promote the migration of human foreskin fibroblast (HSF) cells, representing the first examples of lanthipeptides with cell migration-promoting activity. Comprehensive *in vitro* reconstitution revealed that the regio- and stereoselective AviMeCys macrocyclization is catalyzed by RosX, a newly identified cyclase with a kinase-like fold. Therefore, RosX-like cyclases, originally misannotated as kinase-like proteins, represent a unique class of cyclases that utilize the enethiol group for AviCys/AviMeCys macrocyclization. Furthermore, we demonstrate that Lan formation in rosins follows a substrate-controlled cyclization pathway with kinetic acceleration by the complex of kinase RosK and lyase RosY, which is distinct from the AviMeCys macrocyclization. This study resolves the long-standing ambiguity of enzymatic AviCys macrocyclization and provides a basis for biosynthetic exploration and bioengineering of AviCys-containing natural products across RiPP subfamilies.

## Graphical abstract

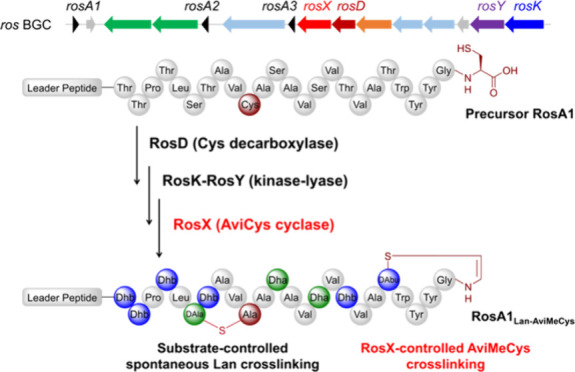

## Introduction

Ribosomally synthesized and posttranslationally modified peptides (RiPPs) have emerged as a major family of natural products with diverse bioactivities and potential as pharmaceutical agents.^1,2^ (2*S*,3*S*)-*S*-((*Z*)-2-aminovinyl)-d-cysteine (AviCys) or (2*S*,3*S*)-*S*-((*Z*)-2-aminovinyl)-3-methyl-d-cysteine (AviMeCys), collectively referred to as Avi­(Me)­Cys motifs,^3^ are unique macrocyclic structures found in diverse RiPP subfamilies, including lanthipeptides, linaridins, thioamitides, and kintamdin-like peptides, and are essential for the antimicrobial and anticancer activities of these natural products.^4–6^ However, the biosynthesis of Avi­(Me)­Cys motifs, especially the key cyclases for the macrocyclization step, is not yet fully understood in all Avi­(Me)­Cys-containing RiPPs, highlighting a significant knowledge gap in understanding the key biosynthetic processes of these unique multicyclic peptide natural products.

Lanthipeptides are one of the largest subfamilies of RiPPs, characterized by the β-thioether cross-linked bis amino acids lanthionine (Lan) and methyllanthionine (MeLan).^2,7^ The biosynthesis of lanthipeptides begins with the ribosomal production of a precursor peptide (LanA), which consists of an N-terminal leader peptide (LP) and a C-terminal core peptide (CP) containing Ser/Thr and Cys residues. LanA is then modified by dehydratase(s) encoded in the biosynthetic gene cluster (BGC) through the dehydration of Ser and Thr residues to generate dehydroalanine (Dha) and dehydrobutyrine (Dhb) residues, respectively. Subsequently, cyclase(s) catalyze the macrocyclization between cysteine and Dha/Dhb to form the characteristic methyllanthionine (MeLan) or methyllabionin (MeLab) cross-links.^8^ Lanthipeptides are now classified into five groups based on the characteristics of their biosynthetic enzymes. Class I lanthipeptides are synthesized by a combination of aminoacyl-tRNA-dependent dehydratases (LanBs) and cyclases (LanCs),^9,10^ whereas class II lanthipeptides are produced by ATP-dependent bifunctional lanthipeptide synthetases (LanMs).^11–13^ The biosynthesis of class III and IV lanthipeptides involves three-domain enzymes: LanKCs and LanLs, respectively, which contain an N-terminal lyase, a central kinase, and a C-terminal cyclase domain.^14–17^

Class V lanthipeptides are the most recently discovered members of this family and can be divided into 2 subgroups. Members of the class V_a_ subgroup contain both the class-defining (Me)­Lan cross-links and a C-terminal (2*S*,3*S*)-*S*-((*Z*)-2-aminovinyl)-3-methyl-d-cysteine (AviMeCys) cross-link, while the class V_b_ subgroup contains only (Me)­Lan cross-links (Figure 1).^18–22^ The biosynthesis of class V lanthipeptides differs notably from that of class I–IV lanthipeptides at the dehydration step by utilizing standalone LanKs (PF01636) and LanYs (PF17914) as kinases and lyases, respectively. Regarding macrocyclization, the class V_b_ group utilizes LanC enzymes for the (Me)­Lan formation.^23^ In contrast, the class V_a_ subgroup requires a minimal set of four enzymes to construct the macrocyclic structures: a kinase-like LanK and an effector-like LanY enzyme for the dehydration of Ser/Thr residues, a flavin-dependent Cys decarboxylase LanD for the oxidative decarboxylation of the C-terminal Cys residue, and a LanX protein that is annotated as a kinase homologue of unknown function. Notably, no gene encoding LanC cyclases or LanC-like cyclase domains has been identified in the class of V_a_ lanthipeptide BGCs. Previous studies have implicated that LanX proteins are important for the AviCys formation. For example, Tao et al. showed that LxmX is essential for the heterologous expression of lexapeptide.^22^ The biosynthetic investigation of thioamitides by Liu et al. indicated that TvaE_S‑87_ and TvaF_S‑87_, homologues of LanX and LanD, respectively, form a minimum AviCys synthetase complex, raising the potential of TvaE_S‑87_ as an AviCys cyclase.^24^ Similarly, Muller, Koehnke, et al. showed that ThoE from thioholgamide biosynthesis facilitates AviMeCys macrocyclization.^25^ However, the catalytic functions of LanX in the Avi­(Me)­Cys macrocyclization have not been experimentally confirmed. As a result, the mechanisms of Avi­(Me)­Cys formation in class V_a_ lanthipeptides remain unclear, leaving an important gap in our understanding.

**Figure 1:**
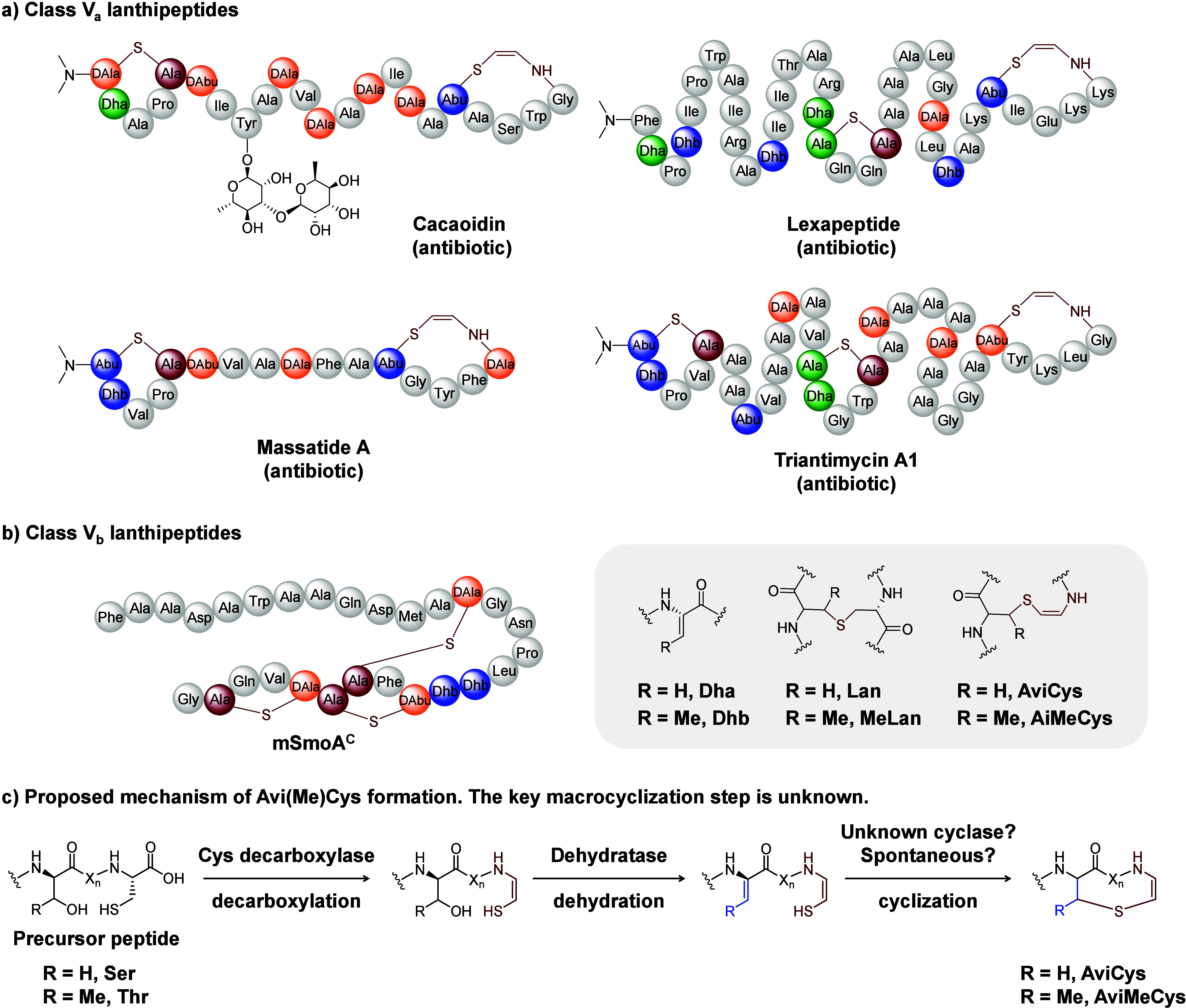
Structures of representative class V lanthipeptides and the proposed mechanism of Avi­(Me)­Cys macrocyclization. (a) Class V_a_ lanthipeptides. (b) Class V_b_ lanthipeptide mSmoA. (c) The proposed mechanism of Avi­(Me)­Cys macrocyclization.

Herein, we report the discovery, structural characterization, and biosynthesis of bicyclic class V_a_ lanthipeptides, named rosins, which contain a Lan ring and a C-terminal AviMeCys ring. The successful *in vitro* reconstitution and characterization of biosynthetic enzymes of rosins reveal that the regio- and stereoselective AviMeCys macrocyclization is catalyzed by RosX, a newly identified cyclase with a kinase-like fold. RosX-like cyclases (LanX proteins) represent the first class of cyclases that utilize the enethiol group for Avi­(Me)­Cys macrocyclization, expanding our understanding of the diverse enzymatic strategies for macrocycle formation in RiPPs. Furthermore, our study shows that the Lan ring is generated through a substrate-controlled Cys-to-Dha Michael-type addition, which is facilitated by the complex of kinase RosK and lyase RosY. This work furthers the appreciation of diverse enzymatic mechanisms in RiPP biosynthesis and facilitates efforts for expanding structural diversity of Avi­(Me)­Cys-containing cyclic peptides using synthetic biology methods.

## Results

### Discovery, Structural Elucidation, and Bioactivity Evaluation of Class V_a_ Lanthipeptide Rosins

We performed targeted genome mining of genomes (Taxonomy ID: 1760) from the NCBI Reference Sequence database. Using cblaster tool^26^ for remote homology search, we queried the sequences of LxmK, LxmY, LanX, and LxmD—key enzymes involved in lexapeptide biosynthesis,^22^ which revealed 851 putative class V_a_ lanthipeptide biosynthetic gene clusters (BGCs). As class V_a_ lanthipeptides are typically *N*-methylated antibiotics,^18–22^ we focused on a subgroup of candidate BGCs (151 in total, Table S1) lacking genes encoding *N*-methyltransferases in hope of new structures and biofunctions. Finally, we selected a putative class V lanthipeptide BGC spanning 14.3 kb from the strain of ATCC 12950, which we designated the *ros* gene cluster. The *ros* BGC consists of genes encoding three putative precursor peptides RosA1/A2/A3, a putative Ser/Thr kinase RosK, a putative lyase RosY, a flavin-dependent cysteine decarboxylase RosD, a unknown function protein RosX, two M16B family peptidases RosP1 and RosP2, a F_420_H_2_-dependent reductase RosJ, and three ATP-binding cassette (ABC) transporters RosT1/T2/T3 (Figure 2a, Table S2). Precursor peptides RosA1/A2/A3 contain leader peptides (LPs) that are homologous to known class V lanthipeptides (Figure S1).

**Figure 2:**
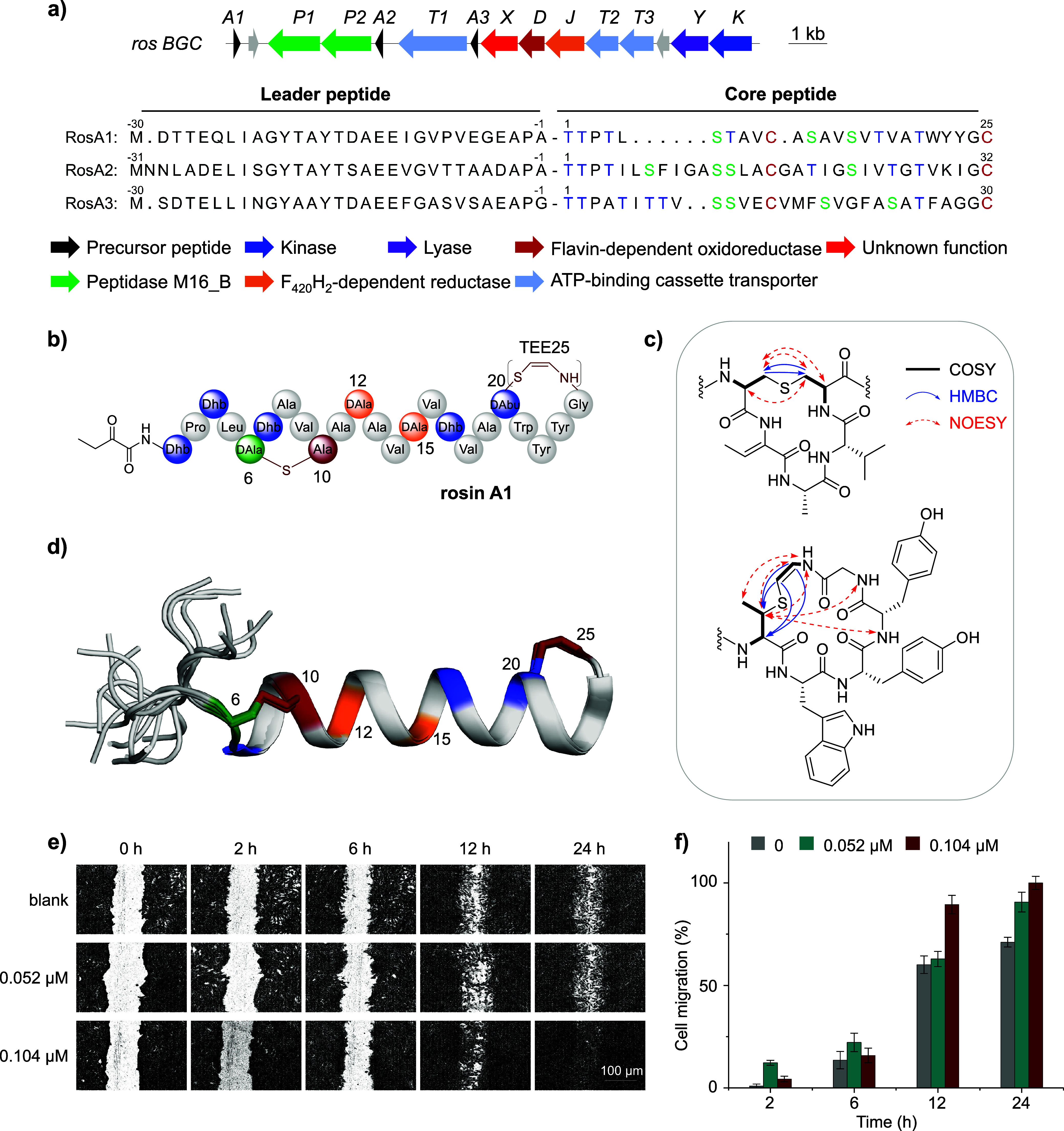
The *ros* gene cluster, the chemical structure of rosin A1 and its pro-migratory activity in HSF cells. (a) The *ros* BGC and the sequences of precursor peptides. (b) The planar structure of rosin A1. (c) The key (2D) NMR correlations of the Lan and the AviMeCys. (d) Ensemble of the 20 lowest energy conformers derived from (2D) NMR analysis. Residues Ala6, Ala10, Ala12, Ala15, Abu20, and TEE25 are colored to match the structure in (b). (e) Wound-healing assay of HSF cells with rosin A1. (f) Rosin A1 promotes the migration of HSF cells in a dose-dependent manner.

We cloned the entire *ros* BGC into the pSET152 vector, yielding the recombinant plasmid pROS (Figure S2a), and expressed it in TK24. Three peptide products, rosin A1/A2/A3, were detected in the culture medium with masses of [M + 3H]^3+^ *m*/*z* = 783.7111, 940.1753, and 907.7613, respectively (Figure S2b,c). Since rosin A1 (0.2 mg/L) was the predominant product, we focused on the structural characterization and biosynthesis of rosin A1 (Figure S3).

Deletion of *rosK*, *rosY*, *rosX*, or *rosD* genes in the pROS plasmid abolished the production of rosin A1/A2/A3 in the TK24 (Figure S4), indicating that these four genes are essential for their production. F_420_H_2_-dependent LanJ reductases are responsible for the reduction of Dha residues to Ala residues^22^ in class V lanthipeptide biosynthesis. Deletion of the *rosJ* gene in the pROS plasmid resulted in the accumulation of a product RosA1_Δ*rosJ*_ with a mass of 4 Da less than rosin A1 ([M + 3H]^3+^ *m*/*z* = 782.3681, Figures S4 and S5), suggesting that the F_420_H_2_-dependent reductase RosJ catalyzed the reduction of two Dha residues during rosin A1 biosynthesis.

The mass of rosin A1 matches that of the RosA1 core peptide (RosA1_CP_) after a combination of modifications, including 9-fold dehydration of Ser/Thr residues, oxidative decarboxylation of the C-terminal Cys, 2-fold Dha/Dhb reduction, and the deamination of an N-terminal Dhb residue. Treatment of rosin A1 with the thiol-selective modification reagent iodoacetamide (IAA) and the Dha modification reagent β-mercaptoethanol (βME) resulted in no modification, indicating the absence of free Cys or Dha residues (Figure S6a). High-performance liquid chromatography (HPLC) coupled with electrospray ionization-quadrupole-time-of-flight tandem mass spectrometry (ESI-QTOF-MS/MS) analysis of rosin A1 suggested the presence of two ring structures between residues Ser6-Cys10 and Thr20-Cys25. MSMS analysis also revealed the presence of an N-terminal 2-oxobutyryl group (Obu), which is likely generated from the spontaneous deamination of an N-terminal Dhb residue after the leader removal (Figure S6b).^27,28^

To analyze the amino acid components of rosin A1, we conducted Marfey’s analysis for rosinA1 using 1-fluoro-2,4-dinitrophenyl-5-l-alanine amide (L-FDAA) (Figure S7).^29^ The results revealed the presence of l-enantiomers of Pro, Leu, Val, Trp, and Tyr, consistent with the RosA1_CP_ sequence. Derivatives of Ser and Thr residues were absent, supporting the proposed 9-fold dehydration of RosA1_CP_ during rosin A1 biosynthesis. Additionally, the analysis identified both l- and d-Ala residues with a peak area ratio of 2:1. Since RosA1 contains four l-Ala residues in total, rosin A1 should contain two d-Ala residues. Combined with the MS/MS analysis, two d-Ala residues are located at the 12 and 15 positions.

The structure of rosin A1 was further characterized by a suite of two-dimensional (2D) NMR spectra, including ^1^H–^1^H COSY, NOESY, ^1^H–^13^C HSQC, HMBC, and ^1^H–^15^N HSQC (Figures S8–S17, Table S3). Abundant ^1^H–^1^H and ^1^H–^13^C correlations between the former Ser6 and Cys10, and between residues 20 and 25, observed in the NOESY and HMBC spectra, confirmed the presence of a Lan cross-link between the former Ser6 and Cys10, as well as a C-terminal AviMeCys cross-link in rosin A1 (Figure 2b,c, Figures S15 and S17). A *Z*-geometry of the double bond in the AviMeCys cross-link was determined based on the corresponding ^3^ *J*_H,H_ value of 7.2 Hz (Figure S8).

To further elucidate the configuration of the Lan and AviMeCys cross-links, rosin A1 was hydrolyzed by HCl, derivatized by FDAA and analyzed by LC-MS. L-FDAA derivative of the Lan cross-link (*m*/*z* 713.16) was detected and determined to be in the DL-configuration (Figure S18a), following a protocol recently developed by the van der Donk group.^30^

The FDAA derivative of D-aminobutyric acid (D-Abu) (*m*/*z* 356.12) was also detected in the hydrolysate of rosin A1 (Figure S18b), which is derived from the acidic hydrolysis of the AviMeCys cross-link.^19^ Based on Marfey’s analysis, the α-C of the AviMeCys cross-link was determined to adopt the “*S*” configuration. NOE correlations were observed between Abu20-Hα (the αH of the former Thr20, 3.47 ppm, dd), Abu20-HN (9.31 ppm, d), and Abu20-Hγ (1.29 ppm, d), as well as between Abu20-Hγ and Ala19-Hβ. Moreover, strong NOE correlations were observed between Abu20-Hβ and TEE25-HN (the NH of the former Cys25), along with relatively weaker correlations between Abu20-Hβ and Tyr23-HN, Gly24-HN, and TEE25-Hα (Figures S12, S17, and S18c). Combined with Marfey’s analysis, these NOE correlations support that the AviMeCys cross-link adopts the (2*S*,3*R*) configuration.^19,31^

With the configuration of the Lan and the AviMeCys cross-links characterized, three-dimensional conformations of rosin A1 were modeled using ^1^H–^1^H distance constraints derived from 2D ^1^H–^1^H NOESY spectra (PDB Code 9LYH, Table S4). Results show that rosin A1 adopts a α-helical structure extended from residue Ala8 to Tyr23 and is stabilized by the Lan and AviMeCys cross-links. The α-helix formation is further supported by its characteristic CD spectra (Figure S19). The N-terminal five-residue segment displays flexible conformations in the 3D NMR structural model (Figure 2d).

The bioactivity of rosin A1 was subsequently evaluated. Although class V lanthipeptides usually display antimicrobial activities, rosin A1 showed no growth inhibitory effect against a range of bacterial strains, including , , , , and . We then assessed the effect of rosin A1 on the migration of HSF cells (derived from human superficial skin tissue) *in vitro*. Results showed that rosin A1 significantly promoted the migration of HSF cells in a dose-dependent manner, as observed in a scratch wound healing experiment (Figure 2e,f, Figure S20a–c). The unmodified RosA1_CP_, in contrast, exhibited no such activity (Figure S20d–f), indicating the modifications are essential for the bioactivity of rosin A1. Rosin A1 therefore represents the first example of a lanthipeptide with cell-migration-promoting activity, expanding the functional diversity of this remarkable RiPP family.

### The RosK-RosY Complex Catalyzes the Dehydration of RosA1 to Initiate the Macrocyclization of the Lan_6–10_ Ring

Expression of His_6_-RosK and His_6_-RosY in both yielded soluble proteins (Figure S21). RosK forms dimer under neutral buffer conditions and RosY exists as a monomer, as determined by gel electrophoresis and size exclusion chromatography (SEC) analysis (Figure 3a, Figure S22a). When His_6_-RosK was coexpressed with RosY, a trimeric RosK-RosY complex in 2:1 ratio was copurified via immobilized metal affinity chromatography (IMAC) (Figure S22a). Such a trimeric complex can be reconstituted by mixing RosK and RosY proteins in 2:1 ratio *in vitro* (Figure S22b). This is distinct from their homologues CaoK-CaoY from cacaoidin biosynthesis^32,33^ and SpaC-SpaD from thiosparsoamide biosynthesis (Figure S22a),^6^ which form heterodimers in solution. Maximum likelihood phylogeny analysis indicates that RosK-RosY are evolutionarily distant from CaoK-CaoY and SpaC-SpaD, which may explain the differences in their oligomerization patterns (Figure S22c).

**Figure 3:**
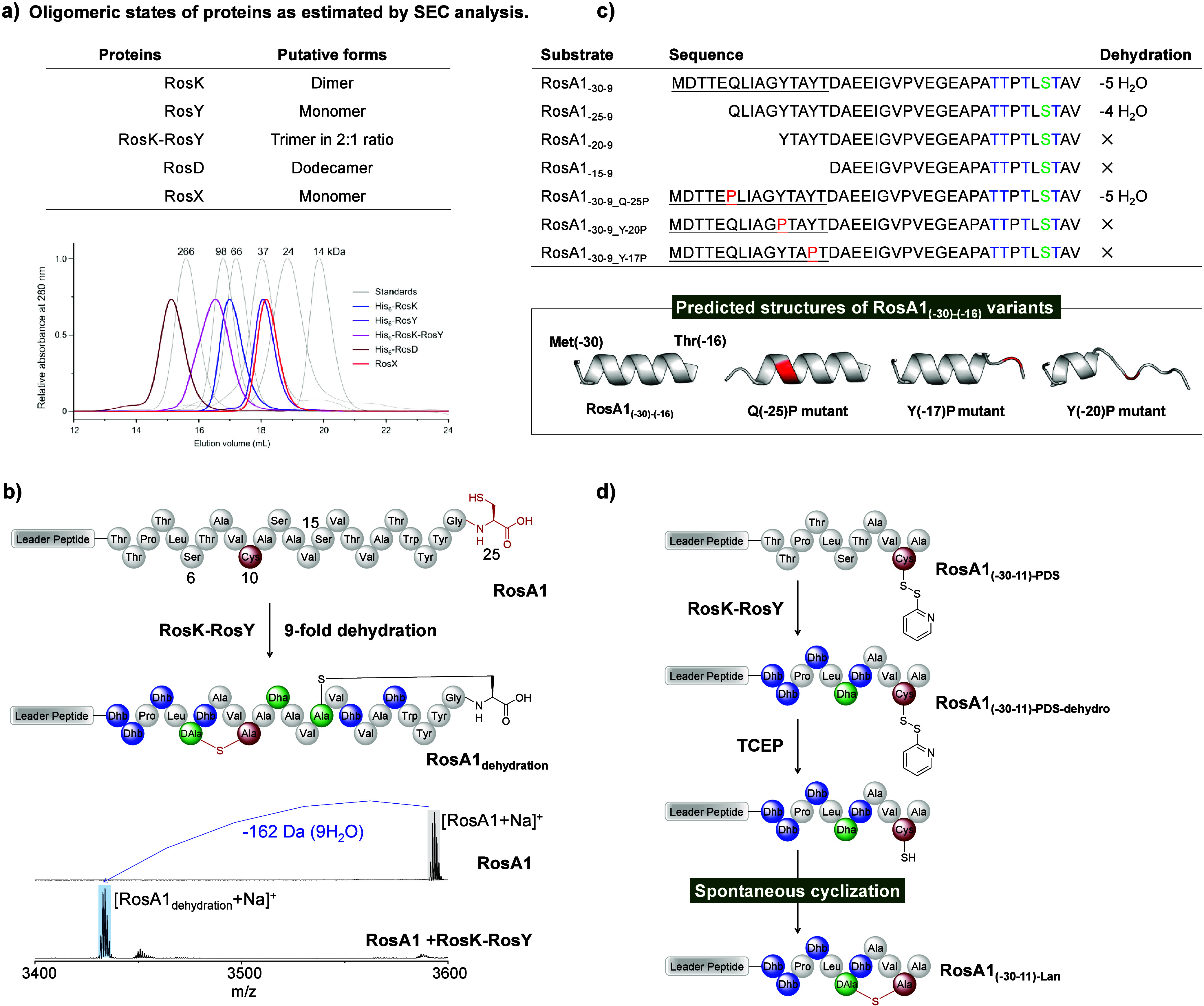
RosK-RosY complex dehydrates RosA1 by recognizing the N-terminal helical segment of the leader peptide and spontaneous cyclization of the Lan. (a) Oligomeric states of RosK, RosY, RosK-RosY, RosD and RosX based on SEC results (see Figure S22 for details). (b) MALDI-TOF-MS analysis of the dehydration of RosA1 by RosK-RosY. [RosA1 + Na]^+^: *M*_calc_. = 3593.72 Da, *M*_obs_. = 3593.58 Da; [RosA1_dehydration_ + Na]^+^: *M*_calc_. = 3431.62 Da, *M*_obs_. = 3431.57 Da. (c) Modification of RosA1_–30–9_ variants by RosK-RosY, and the structural prediction of RosA1 leader peptides by AlphaFold3. (d) Spontaneous cyclization of the DL-Lan cross-link in RosA1_–30–11_ peptide.

The successful purification of RosK and RosY as separate proteins allowed the measurement of their binding mode with each other and the peptide substrates. Microscale thermophoresis (MST) analysis showed that the RosK dimer binds to RosY with a *K*_D_ of (5 ± 1) μM. The dimeric RosK or RosY alone showed weak to neglectable binding affinity toward the leader peptide of RosA1 (RosA1_LP_). The leader-binding affinity was significantly enhanced when RosK and RosY form a trimeric complex (*K*_D_ = 15 ± 6 μM with RosA1_LP_) (Figure S23). These results demonstrate that the formation of the RosK-RosY complex is crucial for their substrate binding and subsequent modification. To characterize the enzymatic activity of RosK, the RosA1 peptide was incubated with RosK in the presence of ATP and Mg^2+^ ions. Results showed that RosK catalyzed 2-fold phosphorylation at the Thr2 and Ser6 residues of RosA1, confirming its kinase activity (Figure S24). The RosK-RosY complex efficiently converted RosA1 to the 9-fold dehydration product RosA1_dehydration_, as determined by MALDI-TOF-MS analysis (Figure 3b).

To dissect the leader recognition mechanism of RosK-RosY, we synthesized a series of RosA1 variant peptides containing a truncated core peptide. RosA1_(−30)‑core9_ was efficiently dehydrated by RosK-RosY up to 5 times. Under identical reaction conditions, RosK-RosY modified RosA1_(−25)‑core9_ with an impaired efficiency. Additional truncation in leader peptide of RosA1 resulted in no modification by RosK-RosY (Figure 3c, Figure S25). AlphaFold3 prediction^34^ suggests that residues −18 to −26 of RosA1_LP_ form an α-helix structure. Proline mutations at positions −20 and −17 disrupt this α-helix structure in RosA1_LP_ and abolished the modification of peptides RosA1_–30–9_Y‑20P_ and RosA1_–30–9_Y‑17P_ by RosK-RosY (Figure 3c and Figure S26). Together, these data suggest that the α-helix structure in RosA1_LP_ is crucial for substrate recognition by RosK-RosY.

Next, we characterized the structure of the dehydration product RosA1_dehydration_ (Figure 3b). Treatment of RosA1_dehydration_ by IAA resulted in no mass change, suggesting that RosA1_dehydration_ contains no free Cys (Figure S27a). LC-MS/MS analysis of the RosA1_dehydration_ indicated the formation of two Lan ring structures between the Ala6-Cys10 and Ala15-Cys25 (Figure 3b and Figure S27b). Incubation of RosA1_dehydration_ with βME led to one-fold βME addition, and MSMS analysis confirmed that the βME addition occurred at Dha12 (Figures S27a and S28). Although the Ala6-Cys10 Lan cross-link (Lan_6–10_) was identical to that in the authentic rosin A1, the C-terminal Ala15-Cys25 Lan cross-link was formed with distinct regioselectivity compared with the AviMeCys cross-link in rosin A1. We further performed time-course assays of RosK-RosY activity. At abbreviated reaction times (20–30 min), we detected intermediates with only 7–8 dehydrations. LC-MS/MS analysis revealed that Thr17 and Thr20 were the last dehydration sites, and the Cys25-to-Dha15 Lan cross-linking was already evident in these partially dehydrated intermediates (Figure S27c,d), indicating its rapid formation.

To understand the mechanism of the Lan_6–10_ formation, peptide RosA1_–30–11_ was synthesized as a model substrate and incubated with RosK-RosY (Figure S29a). MS/MS analysis showed that the RosK-RosY complex efficiently catalyzed the dehydration and the Lan_6–10_ cross-linking in RosA1_–30–11_, producing the expected product RosA1_(−30–11)‑Lan_ (Figure S30). Marfey’s analysis confirmed the Lan configuration in RosA1_(−30–11)‑Lan_ as DL-Lan, which is identical to that of the authentic rosin A1 (Figure S29b). Collectively, these results show that the RosK-RosY complex is sufficient for the generation of the Lan_6–10_ ring in rosin A1.

To specifically explore the mechanism of the Lan_6–10_ cross-linking, dehydrated peptide RosA1_(−30–11)‑PDS‑dehydro_ was synthesized with the Cys residue protected by the PDS group (Figure 3d, Figure S31a).^35^ After deprotecting the Cys residue with TCEP in HEPES buffer (pH 8.0), RosA1_(−30–11)‑PDS‑dehydro_ spontaneously cyclized to form RosA1_(−30–11)‑Lan_ with the Lan_6–10_ cross-link in the correct dl-configuration. This result indicated that the Lan_6–10_ macrocyclization proceeded nonenzymatically and was independent of the C-terminal core peptide segment (Figure 3d, Figure S31b–e). Addition of RosK-RosY along with TCEP to the solution of RosA1_(−30–11)‑PDS‑dehydro_ improved the efficiency of the Lan_6–10_ cyclization moderately (Figure S32). The Thr7-to-Ala mutation in RosA1_(−30–11)‑PDS‑dehydro_ abolished the stereoselectivity of the Lan_6–10_ cross-linking to generate a mixture of dl-Lan_6–10_ and ll-Lan_6–10_ cross-links in near 1:1 ratio, regardless of the absence or the presence of the RosK-RosY complex (Figure S29c,d and S31f–j). Together, these results show that the regio- and stereoselective formation of the Lan_6–10_ ring in RosA1 occurs nonenzymatically and is highly dependent on the “Dha-Dhb-Xxx-Xxx-Cys” sequence within the peptide. Similar substrate-controlled macrocyclization are observed in several class II and class V_a_ lanthipeptides containing (Dha/Dhb)_2_-Xxx-Xxx-Cys motifs.^19,36,37^ Although Lan/MeLan cross-links in class V_a_ lanthipeptides are predominantly ll-configured due to their substrate-controlled mechanism, the Lan_6–10_ cross-link in rosin A1 exhibits the rare dl-configuration. Although the RosK-RosY complex is not strictly required for the stereoselective Lan_6–10_ macrocyclization, it appears to enhance the cyclization kinetically, likely by providing additional conformational stabilization to the self-organized dehydrated peptide intermediate.

### RosX Controls the Regio- and Stereoselectivity of the AviMeCys Cross-Linking in RosA1

Both Ser/Thr dehydration and Cys decarboxylation are essential for Avi­(Me)­Cys biosynthesis. We expressed and purified RosD as a soluble N-terminally His tagged fusion protein from . Consistent with typical LanD proteins, RosD forms a dodecamer *in vitro*, as determined by SEC analysis (Figure S22a).^38,39^ Incubation of RosD with His_6_-RosA1 peptide results in the oxidative decarboxylation of the C-terminal Cys residue by producing the product RosA1_decarboxyl_ with a C-terminal enethiol group (Figure 4a, Figure S33), confirming its function as a Cys decarboxylase.

**Figure 4:**
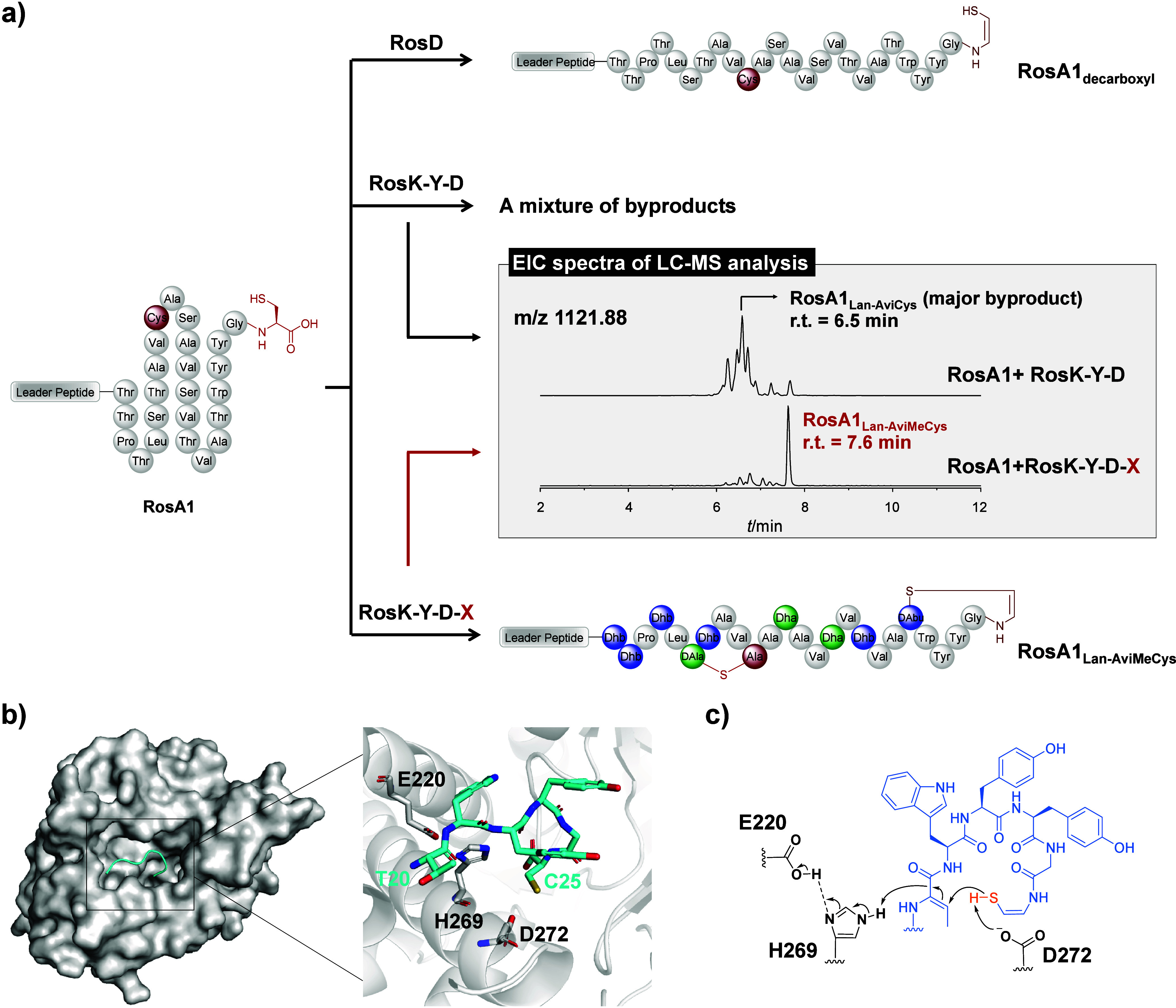
RosX catalyzes regioselective AviMeCys macrocyclization in RosA1. (a) LC-MS analysis of modifications of RosA1 by RosD, RosK-Y-D, and RosK-Y-D-X. EIC profiles of the fully modified products are shown ([M + 3H]^3+^ = 1121.88). (b) Predicted structure of RosX bound with RosA1_C6_, the precursor sequence for the AviMeCys ring. (c) Proposed mechanism for the AviMeCys macrocyclization catalyzed by RosX.

LanX has been proposed to be essential for the Avi­(Me)­Cys biosynthesis in class V_a_ lanthipeptides and thioamitides.^22,24,25^ However, due to the difficulty in acquiring soluble LanX proteins, no functional reconstitution of LanX *in vitro* has been reported to date. We constructed an MBP-RosX fusion protein and successfully obtained soluble, monomeric RosX protein in high purity after the removal of the MBP tag via TEV proteolytic cleavage (Figures S21 and S22a). To reconstitute the biosynthesis of the AviMeCys ring *in vitro*, RosA1 peptide was incubated with RosK, RosY, RosD and RosX, which resulted in the generation of product RosA1_Lan‑AviMeCys_. LC-MS/MS analysis indicated that RosA1_Lan‑AviMeCys_ displayed a ring topology identical with that of the authentic rosin A1 (Figure 4a, Figure S34). Furthermore, incubation of RosA1_Lan‑AviMeCys_ with IAA led to no modification, and βME derivatization of RosA1_Lan‑AviMeCys_ resulted in 2-fold βME addition at residues Dha12 and Dha15, supporting that the formation of the Lan and AviMeCys rings is in the correct regioselectivity (Figure 4a, Figure S35).

To verify the configuration of the AviMeCys motif generated *in vitro*, RosA1_Lan‑AviMeCys_ and rosin A1 were digested by proteinase K, and the resulting AviMeCys macrocycle fragments were analyzed by LC-MS. Results show that the AviMeCys macrocycles derived from RosA1_Lan‑AviMeCys_ and rosin A1 have the same retention time (Figure S36). We further conducted Marfey’s analysis on these two AviMeCys macrocycle samples. Since the Abu motif represents the sole stereocenter generated during macrocyclization in both compounds, we focused on its analysis. Results showed that the Abu residues in AviMeCys-1 and authentic AviMeCys exist as single isomers exclusively in the d-configuration (Figure S37). This result further supports that the enzymatically synthesized AviMeCys macrocycle in RosA1_Lan‑AviMeCys_ is structurally identical to that in the naturally derived rosin A1, confirming the fidelity of the *in vitro* enzymatic modifications.

Due to the difficulty to access the full length RosA1 by heterologous expression in or chemical peptide synthesis, we synthesized peptide RosA1_C9_ as a simplied model peptide, which contains the C-terminal sequence for the AviMeCys formation as the core peptide (Figure S38). After modification by the combination of RosK-Y-X-D enzymes *in vitro*, an AviMeCys ring was installed in RosA1_C9_ efficiently by producing RosA1_C9‑AviMeCys_ (Figures S36, S38, and S39). Marfey’s analysis of the amino acid components of RosA1_C9‑AviMeCys_ detected the AviMeCys-derived d-Abu product as a single isomer (Figure S37), indicating that the AviMeCys cross-link in RosA1_C9‑AviMeCys_ is in the “*S*” configuration identical with that in the authentic rosin A1. Thus, RosA1_C9_ is a suitable substrate to specifically probe the enzymatic formation of the AviMeCys macrocycle.

We next focused on the enzymatic role of RosX in AviMeCys macrocyclization. RosX binds to RosA1_LP_ with a *K*_D_ of (6 ± 2) μM (Figure 5b, Figure S40). Truncation of the predicted α-helix spanning residues −18 to −26 in RosA1_LP_ significantly decreased the level of RosX-RosA1_LP_ binding (Figure S40), which is similar to that observed in the RosA1_LP_ recognition by RosK-RosY (Figure 3c). In addition, RosX displays tight binding to RosD with a *K*_D_ of (547 ± 54) nM, but negligible binding to the RosK-RosY complex (*K*_D_ > 67 ± 8 μM) (Figure 5b, Figure S41).

**Figure 5:**
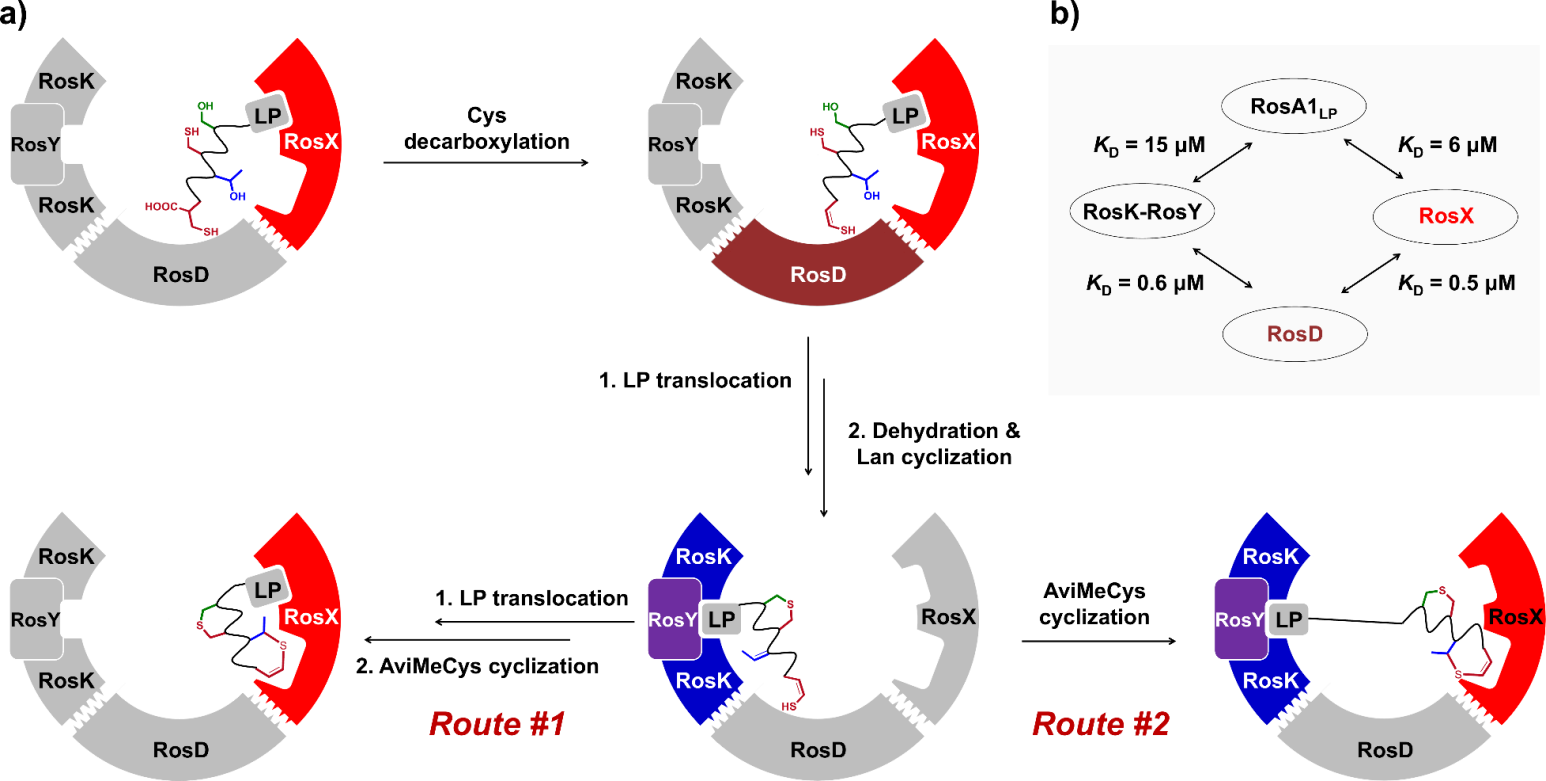
Proposed mechanism of the Lan and AviMeCys macrocyclization in rosin A1 biosynthesis. (a) Proposed model of Lan and AviMeCys formation catalyzed by RosX, RosD, RosK, and RosY. (b) Binding affinities between the RosK-RosY complex, RosD, RosX, and RosA1_LP_.

Omission of RosX from the RosK-Y-D-X combination abolished the conversion of RosA1 to RosA1_Lan‑AviMeCys_ *in vitro*. Instead, LC-MS analysis detected a mixture of byproducts with molecular weights identical to those of RosA1_Lan‑AviMeCys_ but distinct retention times, indicating that RosA1 was fully dehydrated and decarboxylated by RosK-Y-D but not correctly cyclized (Figure 4a). One major byproduct is RosA1_Lan‑AviCys(15–25)_ with an AviCys cross-link between residues Dha15 and Cys25 (Figure 4a, Figure S42). Similarly, modification of RosA1_C9_ peptide by the RosK-Y-D combination generated product RosA1_C9‑AviCys(1–9)_ containing an AviCys cross-link between Ala1 and Cys9 residues, instead of the desired Abu4-Cys9 AviMeCys cross-link (Figures S37 and S43). The AviCys cross-linking in byproducts RosA1_Lan‑AviCys(15–25)_ and RosA1_C9‑AviCys(1–9)_ is likely due to the nonenzymatic cyclization driven by the high reactivity of Dha residues. Overall, these results show that RosX is critical for regioselective AviMeCys macrocyclization in rosin A1.

Next, we generate a modeled structure of RosX with high confidence by AlphaFold3,^34^ as indicated by the predicted template modeling (pTM) score (Figure S44a). A DALI search^40,41^ of the rank_1 predicted structure model against the Protein Data Bank (PDB)^42^ reveals that the closest structural homologue of RosX is the kinase LxmK from the biosynthesis of class V_a_ lanthipeptide lexapeptide (PDB Code 9DK3; *Z*-score of 20.7 and 282 Cα residues aligned with an RMSD of 3.5 Å),^43^ despite their low sequence identity (∼17%). RosX adopts a kinase-like fold in the predicted structure, consisting of an N-terminal lobe that is variable among kinases and a highly conserved C-terminal lobe (),^44^ but lacks putative catalytic and Mg^2+^ binding residues conserved in typical kinases. Specifically, key residues present in LxmK, such as those involved in catalysis (Asp221), Mg^2+^ coordination (Asp241 and Gln226), and ATP binding (Arg49 and Lys66),^43^ are completely absent in RosX (Figure S44c,d).

We further utilized AlphaFold3 ^34^ to model the complex structure of RosX with hexapeptide RosA1_20–25_, the precursor of the AviMeCys ring in rosin A1 (Figure 4b, Figure S45a–c). The predicted complex structure suggests that RosA1_20–25_ binds within a hydrophobic cavity in RosX, adopting a helix-like conformation. In this model, the thiol group of Cys25 oriented toward the side chain of Thr20. Key amino acids in the cavity, including Glu220, His269, and Asp272, are highly conserved among LanX proteins but conspicuously absent in LanK proteins (Figure S45d,e). These residues are positioned in close contact with the enethiol and Dhb motifs in the predicted model and may act as general acids or bases during Michael-type addition (Figure 4c). Indeed, Ala mutations of residues Glu220, His269, and Asp272 completely disrupted the proper enzymatic formation of the AviMeCys cross-link in RosA1, generating a mixture of products similar to the system lacking RosX (Figure S46). These findings further confirm that RosX is the enzyme responsible for the regioselective AviMeCys formation and represents a unique Avi­(Me)­Cys cyclase with a kinase-like fold.

## Discussion

Macrocyclization in cyclic peptide natural products is essential for stabilizing these molecules in specific 3D conformations. Class V lanthipeptide RosA1 adopts an elongated helical conformation, which is stabilized by the Lan and AviMeCys cross-links. Preliminary structural analyses suggest that RosA2 and RosA3 also adopt similar α-helical conformations (Figures S47–S49). This feature is relatively rare in lanthipeptides, only reported in lichenicidin VK2146, cytolysin, and mSmoA^C^.^23,27,45^ Notably, structural predictions reveals a conserved helix-forming propensity across class V_a_ core peptides, and the potential (Me)­Lan and Avi­(Me)­Cys cross-linking residues are always arranged in (*i*, *i* + 3) or (*i*, *i* + 4) spacing patterns (Figure S50). These findings suggest that the (Me)­Lan and Avi­(Me)­Cys cross-links function as “molecular staples” in class V_a_ lanthipeptides, indicating evolutionary optimization for their conformational stability.

The factors influencing the macrocyclization in RiPPs exhibit significant variation across different systems, particularly for multicyclic peptides. Both enzymes and substrates contribute to the regioselectivity and stereoselectivity of the cyclization process. The discovery of RosX-like proteins as a novel class of Avi­(Me)­Cys cyclases, previously misannotated as kinase homologues, addresses a critical gap in the biosynthetic understanding of Avi­(Me)­Cys macrocycles. The Lan ring and the AviMeCys ring are generated independently and via distinct enzymatic mechanisms. The macrocyclization of the Lan ring follows a substrate-controlled mechanism, where the (Dhx)_2_–Xxx–Xxx–Cys sequence adopts a preorganized helical structure that dictates the regio- and stereochemistry of cyclization. The RosK-RosY complex moderately accelerates the cyclization reaction. In contrast, the regioselectivity of AviMeCys cross-linking is strictly controlled by the cyclase RosX. In the absence of RosX, the enethiol group preferentially conjugates to a Dha residue over a Dhb residue, aligning with inherent electrophilicity trends but resulting in a non-native regiopattern of AviCys cross-linking. Notably, the Zn-free catalytic pocket of RosX displays a divergent structural organization from LanC cyclases, highlighting an evolutionary adaptation for enethiol-mediated cross-linking rather than the canonical Cys thiol-based cyclization observed in LanC enzymes.

*In vitro* reconstitution of AviCys formation provides insights into the biosynthetic order. The spontaneous Cys25-to-Dha15 cross-linking in the (RosK-RosY)-dehydrated RosA1 as a side reaction suggests that the RosD-catalyzed Cys decarboxylation should occur prior to the dehydration during the AviMeCys biosynthesis. This ordering of enzymatic steps is supported by *in vitro* modification experiments, where the addition of RosD and RosX before RosK-RosY resulted in the optimal generation of the expected AviMeCys product, compared to other conditions tested (Figure S51). RosX likely accommodates the C-terminal segment of decarboxylated RosA1, preventing the hydrolytic side reactions of the unstable C-terminal enethiol intermediate before dehydration and macrocyclization. Such a protective role of RosX-like proteins is also observed in biosynthesis of thioamitide TVA and thioholgamide, where TvaE_S‑87_ and ThoE, structural homologues to RosX, protects the thioenol intermediate from hydrolysis.^24,25^ Comparative analysis reveals that RosX shares limited sequence similarity with TvaE_S‑87_ (∼23%), and phylogenetic analysis places them in distinct clades (Figure S52a). RosX and TvaE_S‑87_ display overall structural similarity in their predicted structures with a kinase-like fold (RMSD = 3.8 Å) (Figure S52b). Both RosX and TvaE_S‑87_ lack the canonical catalytic residues and Mg^2+^-chelating motif typical of kinases.^24^

Both the RosK-RosY complex and RosX display similar binding affinity toward RosA1 leader peptide, suggesting a potential dynamic exchange of substrate binding among modification enzymes during biosynthesis, which may coordinate the formation of both the Lan and AviMeCys rings. Additionally, RosD binds to RosX (*K*_D_ = 547 ± 54 nM) and the RosK-RosY complex (*K*_D_ = 647 ± 17 nM) with similar binding affinity (Figure 5b and Figure S41). Genome mining also reveals that in some class V_a_ lanthipeptide BGCs, LanD and LanX are fused as a single protein (Figure S53). This suggests that the LanD-LanX complex might remain bound during biosynthesis, further emphasizing the possibility of a coordinated multienzyme complex in the catalysis of AviMeCys formation. Based on these findings, we propose that the Lan and AviMeCys macrocyclization was initiated by the RosD-catalyzed Cys decarboxylation with RosA1_LP_ bound to RosX (Figure 5a). Subsequently, RosA1_LP_ translocates to the binding site in RosK-RosY, where the dehydration of RosA1 and the spontaneous Lan cross-linking occur. The AviMeCys macrocyclization then proceeds in the active site in RosX, with the RosA1_LP_ bound to RosK-RosY (route #1) or exchanged to the leader binding site in RosX (route #2). The formation of a four-component complex facilitates the precise and controlled formation of the AviMeCys macrocycle. This insight opens new avenues for bioengineering multicyclic peptides with controlled and tailored modifications.

## Supplementary Material





## Notes

Detailed materials and methods for protein expression, purification, and biochemical characterization, detailed structural elucidation and NMR data of rosin A1, and additional data including sequence alignment, size exclusion chromatography, MALDI-TOF MS analysis, mass spectra, and tandem MS analyses (PDF)
Transparent Peer Review report available (PDF)
