# Supplementary material for: A Unique Class of Cyclases with a Kinase Fold Catalyzes Enethiol-Mediated Macrocyclization of Aminovinyl-Cysteine Motifs in Lanthipeptides
Source: ACS Cent Sci. 2025 Jun 18;11(7):1178–88. doi: 10.1021/acscentsci.5c00569 (PMC12291118; doi:10.1021/acscentsci.5c00569)
Supplement: Supplementary file 1 [file oc5c00569_si_001.pdf]

Supporting Information

A Unique Class of Cyclases with a Kinase Fold Catalyzes Enethiol-mediated Macrocyclization of Aminovinyl-Cysteine Motifs in Lanthipeptides

Xiang-Qian Xie[1], Wen Guo[1], Yin-Zheng Xia[1], Li-Juan Liao[3], Meng-Xin Sun[1], Jing-Xue Wang[1], Jiang-Tao Gao[3]*, Hong-Wei Yao[2]* and Huan Wang[1]*

[1] State Key Laboratory of Coordination Chemistry, Chemistry and Biomedicine Innovation Center of Nanjing University, Jiangsu Key Laboratory of Advanced Organic Materials, School of Chemistry and Chemical Engineering, Nanjing University, Nanjing, 210093, China

[2] Institute of Molecular Enzymology, School of Life Sciences, Suzhou Medical College of Soochow University, Soochow University, Suzhou, 215123, China

[3] State Key Laboratory of Ecological Pest Control for Fujian and Taiwan Crops, College of Life Sciences, Fujian Agriculture and Forestry University, 350002, Fuzhou, China

*e-mail: wanghuan@nju.edu.cn; hwyao@suda.edu.cn; jgaotao@gmail.com

Supplemental Materials and Methods

Materials. All oligonucleotides were purchased from Genscript Biotech (Nanjing, China). Restriction endonucleases were purchased from New England Biolabs (Ipswich, MA, USA). Phanta® Max Master Mix and ClonExpress II/MultiS one Step Cloning Kits were purchased from Vazyme Biotech (Nanjing, China). Medium components for bacterial cultures were purchased from Thermo Fisher (Waltham, MA, USA). Chemicals were purchased from Aladdin Reagent (Shanghai, China) or Sigma-Aldrich (Schnelldorf, Germany) unless noted otherwise. Endoprotease GluC was purchased from Roche Biosciences (Basel, Switzerland). *E. coli* DH5α was used as the host for cloning and plasmid propagation, and *E. coli* BL21 (DE3) was used as a host for expression of proteins and peptides. *Microbispora rosea* ATCC 12950 was purchased from the China General Microbiological Culture Collection Center (CGMCC). HSFs (derived from human superficial skin tissue), fetal bovine serum (FBS), Dulbecco's modified Eagle medium (DMEM), phosphate-buffered saline (PBS), penicillin-streptomycin solution (double antibody) 100×, and 0.25% trypsin solution were purchased from Xiamen Immocell Biotechnology Co., Ltd. (Xiamen, China). A Cell Counting Kit 8 (CCK-8) was purchased from Beijing Solar Science & Technology Co., Ltd. (Beijing, China).

General methods. Polymerase chain reactions (PCR) were carried out on a C1000 Touch™ thermal cycler (Bio-Rad). DNA sequencing was performed by Sangon Biotechnology (Shanghai, China), using appropriate primers. Matrix-assisted laser desorption/ionization time-of-flight mass spectrometry (MALDI-TOF MS) was carried out on a Bruker UltraFlextreme. Liquid chromatography electrospray ionization tandem mass spectrometry (LC/ESI-MS/MS) was carried out and processed using a Triple TOF 4600 System (AB Sciex) equipped with a Prominence Ultra-Fast Liquid Chromatography (UFLC) system (Shimadzu). Conditions for all ESI-MS and MS/MS were set as follows: nebulizer gas: 55 psi; heater gas: 55 psi; curtain gas: 35 psi; drying temperature: 550°C; ion spray voltage: 5500 V; declustering potential: 100 V; collision energy: 35 V (positive); collision energy spread: 10 V. The mass range and accumulation time are 400-4000 m/z, 250 ms for ESI-MS and 100-2000 m/z, 100 ms for MS/MS, respectively. Collision-induced dissociation (CID) was performed for fragmentation of the respective peptide ions. Calibration solutions purchased from AB SCIEX were used for instrument calibration, and high resolution was chosen in the ESI+ mode. NMR experiments were performed at 298 K on Bruker AVANCE III 600 MHz and AVANCE NEO 800 MHz spectrometers equipped with 5 mm z-gradient $^{1}H/^{13}C/^{15}N$ TCI cryogenic probes. Two-dimensional (2D) ^{1}H-^{1}H COSY, TOCSY, ^{1}H-$^{13}C/^{15}N$ HSQC, and ^{1}H-^{13}C HMBC were measured to obtain chemical shifts. 2D ^{1}H-^{1}H NOESY experiments with 300 ms of mixing time were performed to obtain ^{1}H-^{1}H distance constraints. All NMR spectra were processed using TopSpin 4.4.0 and analyzed using POKY. The chemical shifts for ^{1}H and ^{13}C were referenced to 3.31 ppm (CHD_2OH) and 49.0 ppm (CD_3OH) on the TMS scale, respectively, and ^{15}N chemical shifts were referenced indirectly. The Xplor-

NIH program (version 3.9) was used for the structure determination and refinement. The 20 lowest energy structures were selected from 100 calculated structures for analysis. The Ramachandran statistics obtained from PROCHECK indicate that 81.9% of residues fall within the most favored region, while 14.7% of residues are within the allowed region. Figure generation was performed using PyMOL (version 2.5.4).

General nomenclature. In this report, we use the standardized nomenclature recommended by the lanthipeptide community in 2020[1]. Residue numbering in lanthipeptide precursor peptides begins with the first residue of the core peptide, while residues in the leader peptide are assigned negative numbers, counting backward from the junction between the leader and core peptides.

Bioinformatic Analysis. To systematically identify novel class Va lanthipeptide biosynthetic gene clusters (BGCs), targeted genome mining was performed on *Actinomycetes* genomes (Taxonomy ID: 1760) from the NCBI Reference Sequence (RefSeq) database. Remote homology searches were conducted using cblaster,[2] with the sequences of LxmK, LxmY, LanX, and LxmD (key enzymes involved in lexapeptide biosynthesis) as queries. Stringent search criteria were applied (maximum E-value: 0.01; minimum identity: 30%; minimum coverage: 80%; minimum number of hits: 3), resulting in the identification of 851 candidate BGCs encoding conserved post-translational modification enzymes characteristic of class V_a lanthipeptides. Considering that most characterized class V_a lanthipeptides are N-methylated and exhibit antibacterial activity,[3-7] subsequent analysis focused on identifying structurally and functionally diverse candidates. The RODEO tool,[8] a rule-based genome mining platform, was utilized to further screen the 851 candidates, specifically excluding BGCs encoding methyltransferase genes. This refinement reduced the candidate set to 151 high-confidence BGCs potentially encoding non-N-methylated lanthipeptides. Among these, the *ros* BGC from *Microbispora rosea* ATCC 12950 was prioritized for experimental investigation due to its atypical biosynthetic architecture (featuring three precursor peptides and lacking a methyltransferase) and the commercial availability of the producing strain, enabling downstream validation.

Phylogenetic analysis and Sequence analysis. Phylogenetic trees were constructed using MEGA Software (v.11.0.13, Pennsylvania State University)[9]. Multiple sequence alignments of protein sequences were performed with ClustalW method[10]. Maximum likelihood (ML) phylogenetic trees were generated based on these alignments using the Jones–Taylor–Thornton (JTT) model with 2,000 bootstrap replications, assuming uniform rates among sites and applying partial deletion of gaps/missing data (site coverage cutoff = 95%). Only bootstrap values greater than 70 are shown on the trees. The resulting trees were visualized using MEGA11. Multiple sequence alignment of selected sequences was performed using ClustalW[10] and visualized using ESPript 3.0[11].

Construction of the pROS. The *ros* BGC was first divided into three fragments for PCR amplification from the *Microbispora rosea* ATCC 12950 genome and then directly cloned into the pSET152 vector using In-Fusion DNA assembly, yielding the recombinant plasmid pROS.

Heterologous expression of the *ros* gene cluster in *S. lividans* TK24. pROS was transferred into *S. lividans* TK24 via *E. coli* ET12567 by conjugation. Colonies that could grow on an MS plate with apramycin and nalidixic acid at 30°C and confirmed by PCR and sequencing were identified as target recombinant strain *S. lividans* TK24/pROS. The strain was inoculated into 150 mL YEME medium and cultured under shaking conditions (220 r.p.m.) at 28 °C for 3 days. After centrifugation to remove the culture medium, the cells were extracted with methanol, and 2 μL of the methanol extract was used for LC-HRMS analysis. The heterologous expression of *S. lividans*/pROS-RosA1 and the knockout strains of different genes was performed as described above.

Culture and fermentation. *S. lividans* TK24/pROS-RosA1 was spread on MS agar plates that contained a medium composed of mannitol (20 g/L), soybean meal (20 g/L), and agar (20 g/L), pH 7.0, and then incubated at 28°C. Upon sporulation, approximately 1 cm^2 of the agar was cut, chopped, transferred to 150 mL of the YEME medium, which was composed of yeast extract (4 g/L), malt extract (4 g/L), and glucose (4 g/L), pH 7.0, and then cultured under shaking conditions (220 r.p.m.) at 28°C for 3 days.

Isolation of rosinA1. The fermentation broth of *S. lividans* TK24/pROS-RosA1 (30 L) was centrifuged, and the resulting pelleted mycelial was extracted with methanol (2 L) three times. After filtration to remove the mycelium, the methanol extract was concentrated under reduced pressure. The methanol extract was then dissolved in water, and an equal volume of ethyl acetate was added to extract three times. The organic phase was dried under rotary evaporation and subsequently dissolved in methanol for reverse-phase silica gel column chromatography, which was eluted with H_2O-methanol solvents. Fractions containing rosinA1 were combined, concentrated and purified by RP-HPLC using an Ultimate Polar RP column (250 × 10 mm, 5 μm, Welch Technology Co., Ltd., Shanghai) with a gradient elution of solvent A (H_2O + 0.1% formic acid) and solvent B (acetonitrile + 0.1% formic acid) at a flow rate of 4 mL/min over a 35 min period as follows: T = 0 min, 50% B; T = 5 min, 50% B; T = 25 min, 90% B; T = 27 min, 99% B; T = 35 min, 99% B. Fractions containing rosinA1 were collected, and the solvents were removed under vacuum. The typical yield of rosinA1 was 6 mg from 30 L culture broth.

Molecular cloning. Plasmids containing the target genes were cloned from the genomic DNA of *Microbispora rosea* ATCC 12950 and amplified by PCR following 30 cycles of denaturing (95°C for 15 s), annealing (60°C for 15 s), and extending (72°C, 1 min/kb) using high fidelity Phanta® DNA Polymerase. Amplification of target genes was confirmed by 1.5% agarose gel electrophoresis. The PCR products were purified using an Omega Biotech Gel

Extraction Kit. The target vectors were digested in separate reactions containing 1× NEB buffer (New England Biolabs) with a selected pair of restriction enzymes for 2 h at 37°C. The digested products were purified by agarose gel electrophoresis, and the target DNA fragments were extracted using an Omega Biotech Gel Extraction Kit. The resulting DNA products were ligated by homologous recombination at 37°C for 30 min in 5 × CE II buffer with CE II enzyme. *E. coli* DH5α cells were transformed with 5 μL of the ligation product by heat shock. The resulting cells were plated on Luria-Bertani (LB) agar plates containing appropriate antibiotic(s) and grown for 15 h at 37°C. Single colonies were picked and used to inoculate separate 5 mL cultures of LB medium containing appropriate antibiotic(s). The cultures were grown at 37°C for 12 h, and plasmids were isolated using an Omega Biotech Plasmid Mini Kit. The sequences of the resulting plasmids were confirmed by DNA sequencing. In detail, RosA1 and its variants were cloned into the MCSI site of pRSFDuet-1 vector, while RosK, RosY, RosD and their variants were cloned into the pET-28a(+) vector. RosX was constructed in the pMCSG9 vector, which contains a His_6-MBP-TEV tag. RosK (tagged) was cloned into pRSFDuet-1 MCSI and RosY (untagged) in MCSII for RosK-RosY co-expression.

Overexpression and purification of precursor peptides. *E. coli* BL21(DE3) cells were transformed with pRSFDuet-1-His_6-LanA and plated on a LB agar plate containing 50 mg/L of kanamycin. A single colony was used to inoculate a 5 mL culture of LB supplemented with 50 mg/L of kanamycin at 37°C for 12 h. The culture was used to inoculate 4 L of LB containing 50 mg/L of kanamycin. Cells were grown at 37°C to an optical density (OD_{600}) of roughly 0.6–0.8, cooled to 18°C before isopropyl-β-D-thiogalactoside (IPTG) was added to a final concentration of 0.2 mM. The resulting cultures were grown for another 16 h. Cells were harvested by centrifugation at 12,000 ×g for 30 min at 4°C. The resulting cell pellet was resuspended in 30 mL of start buffer (20 mM NaH_2PO_4, pH 7.5, 500 mM NaCl, 0.5 mM imidazole, 20% glycerol), and the suspension was sonicated on ice for 30 min to lyse the cells. Cell debris was removed by centrifugation at 23,700 ×g for 30 min at 4°C. The supernatant was discarded, and the pellet containing peptide products was resuspended in 30 mL of start buffer. The sonication and centrifugation steps were repeated. Again, the supernatant was discarded, and the pellet was resuspended in 30 mL of buffer 1 (6 M guanidine HCl, 20 mM NaH_2PO_4, pH 7.5, 500 mM NaCl, 0.5 mM imidazole). The sample was sonicated and insoluble material was removed by centrifugation at 23,700 ×g for 30 min at 4°C, followed by filtration of the supernatant through a 0.45 μm filter. The filtered sample was applied to a 5 mL HisTrap HP (GE Healthcare Life Sciences) immobilized metal affinity chromatography (IMAC) column previously charged with $NiSO_4$ and equilibrated in buffer 1. The column was washed with two column volumes of buffer 1, followed by two column volumes of buffer 2 (4 M guanidine HCl, 20 mM NaH_2PO_4, pH 7.5, 500 mM NaCl, 30 mM imidazole). The peptide was eluted with two column volumes of elution buffer (4 M guanidine HCl, 20 mM NaH_2PO_4, pH 7.5, 500 mM NaCl, 1 M

imidazole). The fractions were desalted using a Sep-Pak® C18 Cartridges and analyzed by MALDI-TOF MS and the organic solvents were removed by rotary evaporation, followed by lyophilization. The product was kept at −80°C for long-term storage. Typical yields from 4 L culture were 1 mg for His_6-RosA1.

Overexpression and purification of proteins. *E. coli* BL21 (DE3) cells were transformed with the plasmid containing the gene encoding the target enzymes. A single colony was used to inoculate a 30 mL culture of LB supplemented with appropriate antibiotic (50 mg/L of kanamycin for pET-28a(+), while 100 mg/L of Ampicillin for pMCSG9). The culture was grown at 37°C for 12 h and further used to inoculate three 1 L of LB cultures in 2 L flasks supplemented with appropriate antibiotic. The culture was grown at 37°C to an OD_{600} of roughly 0.6–0.8, and cooled at 4°C on ice for 20 min before the addition of IPTG to a final concentration of 0.2 mM. The culture was grown at 18°C for additional 16 h. Cells were harvested by centrifugation at 12,000 ×g for 15 min at 4°C, and the pellet was resuspended in 30 mL of start buffer (20 mM Tris buffer, pH 8.0, 500 mM NaCl, 1.0 mM TCEP, 10% glycerol).

All protein purification steps were performed at 4°C. The cell paste was suspended in start buffer, and the cells were lysed using a high-pressure homogenizer (Avestin, Inc.). Cell debris was removed via centrifugation at 23,700 ×g for 30 min at 4°C. The supernatant was loaded onto a 5 mL HisTrap HP IMAC column charged with $NiSO_4$ and equilibrated with start buffer. The column was washed with 50 mL of buffer A (30 mM imidazole, 20 mM Tris, pH 7.5, 300 mM NaCl), and the protein was eluted using a linear gradient of 0-100% buffer B (200 mM imidazole, 20 mM Tris, pH 7.5, 300 mM NaCl) over 40 min at a 2 mL/min flow rate. Fractions containing proteins were collected and analyzed by SDS-PAGE. Fractions containing target proteins were combined and concentrated using an Amicon® Ultra Centrifugal Filter Unit (Millipore). The protein sample was purified by gel filtration using an FPLC system (ÄKTA) equipped with an HiLoad™ 16/600 (GE Healthcare Life Sciences) column packed with SuperDex 200 resin. Fractions containing target proteins were collected, combined and concentrated using an Amicon Ultra Centrifugal Filter Unit. The resulting protein sample was stored at −80°C. Protein concentration was determined using a Bradford Assay Kit (Pierce).

Specifically, the RosX protein was expressed as a fusion protein conjugated to the N-terminus of MBP tag. After purification of the MBP-RosX fusion protein following the abovementioned procedure, the MBP tag was removed by TEV protease by overnight incubation at 4°C. The mixture was applied to a HisTrap HP column and eluted with 10% buffer B. The resulting RosX was further purified by an HiLoad™ 16/600 (GE Healthcare Life Sciences) column packed with SuperDex 200 resin as a soluble protein.

***In vitro* enzymatic assays.** To reconstitute the activity of RosK, His_6-RosA1 (100 μM) and His_6-RosK (20 μM) were incubated with 5 mM ATP, 1 mM $MgCl_2$ and 1 mM DTT in 50 mM HEPES, pH 8.0, at 28°C for 3 h.

To reconstitute the activity of the RosK-Y complex, His_6-RosA1 or variants (100 μM) and His_6-RosK-RosY (20 μM) were incubated with 5 mM ATP, 1 mM $MgCl_2$ and 1 mM DTT in 50 mM HEPES pH 8.0, at 28°C for 3 h.

For decarboxylation, His_6-RosA1 (100 μM) and His_6-RosD (20 μM) were incubated with 5 μM FMN, and 1 mM DTT in 50 mM HEPES, pH 8.0, at 28°C for 30 min.

To reconstitute the biosynthesis of the AviMeCys ring, His_6-RosA1 (100 μM) and His_6-RosD (20 μM), RosX (20 μM) were incubated with 5 mM ATP, 1 mM $MgCl_2$, 5 μM FMN and 1 mM DTT in 50 mM HEPES, pH 8.0, at 28°C for 10 min, His_6-RosK-RosY complex (20 μM) was then added to proceed for an additional 4 h.

To analyze each assay, the reactions were quenched by heating at 50°C for 5 min, followed by digestion with GluC (1 μM) for 1 h at 37°C. The supernatant of the reaction mixture was desalted by a SPE column before analysis by MALDI-TOF-MS and LC-MS/MS. LC-MS/MS analysis was performed using a Triple TOF 4600 System (AB Sciex) equipped with a Prominence Ultra-Fast Liquid Chromatography (UFLC) system (Shimadzu) and a C18 reverse-phase column (Phenomenex Aeris™ PEPTIDE XB-C18, 2.6 μm, 4.6×150 mm). The elution gradient for the enzymatic modification of RosA1 was solvent A (H_2O with 0.1% formic acid) and solvent B (acetonitrile) at a flow rate of 0.2 mL/min over a 15 min period as follows: T = 0 min, 5% B; T = 1 min, 5% B; T = 3 min, 30% B; T = 8 min, 70% B; T = 11 min, 90% B; T = 11.1 min, 5% B; T = 15 min, 5% B. The elution gradient for the enzymatic modification of RosA1 variants was carried out using solvent A (H_2O with 0.1% formic acid) and solvent B (acetonitrile) at a flow rate of 0.2 mL/min over a 15 min period as follows: T = 0 min, 5% B; T = 1 min, 5% B; T = 3 min, 25% B; T = 8 min, 40% B; T = 9 min, 90% B; T = 11 min, 90% B; T = 11.1 min, 5% B; T = 15 min, 5% B. Large-scale reactions were performed multiple times at a 500 μL scale using the reaction setup described above. The reactions were quenched by heating at 50°C for 5min, followed by centrifugation at 16,000 g for 20 min (10 kDa Amicon® Ultra-0.5). The filtrate was then directly prepared by HPLC on an Ultimate Polar RP column (250 × 4.6 mm, 5 μm, Welch Technology Co., Ltd., Shanghai), using a gradient elution of solvent A (H_2O with 0.1% formic acid) and solvent B (acetonitrile with 0.1% formic acid) at a flow rate of 1.0 mL/min over a 35 min period as follows: T = 0 min, 30% B; T = 5 min, 30% B; T = 25 min, 90% B; T = 27 min, 99% B; T = 35 min, 99% B.

Modification of free cysteine residues in peptides with IAA. Peptides (100 μM) were typically modified by 1 mM IAA in 20 mM Tris-HCl (pH 8.0) and 0.5 mM TCEP at room temperature in the dark for 30 min. The reaction was then quenched by the addition of 20 mM DTT. The reaction mixture was desalted by a SPE column and further analyzed by MALDI-TOF-MS.

Modification of Dha/Dhb residues in peptides with βME. Peptides (100 μM) were typically treated by βME (0.5 mM) in 20 mM Tris-HCl (pH 8.0) at 37°C for 1 h. The resulting mixture was desalted by a SPE column and further

analyzed by MALDI-TOF-MS.

Modification of free cysteine residues in peptides with DPDS. Peptides (100 μM) were typically modified by 500 μM DPDS in 50 mM HEPES (pH 8.0) at 37°C for 12 h. The supernatant of the reaction mixture was analyzed by HPLC. Peptides protected by PDS were then used as substrates for enzyme activity assays.

Marfey assay for chiral analysis of native amino acid residues. Pure peptides (0.5 mg) were hydrolyzed in 1 mL 6 N HCl (5% thioglycolic acid to prevent the degradation of Trp)[5, 12] in a sealed tube at 110°C for 12 h. The hydrolysate was dried under reduced pressure and dissolved in 20 μL ddH$_2$O, transferred to a 1.5 mL Eppendorf tube, which was followed by the addition of 40 μL of 1% acetone solution of FDAA (N-(5-fluoro-2, 4-dinitrophenyl)-L-alaninamide, Marfey's reagent) and 8 μL of 1 N NaHCO$_3$ solution for derivatization. The reaction mixture was heated with frequent shaking over a hot plate at 40°C for 1 h and cooled to ambient temperature. The reaction was quenched by the addition of 4 μL of 2 N HCl and diluted with 200 μL MeOH. Standards (D/L-amino acid) were treated identically. Nisin and (LL-, DL-lanthionine) were obtained from Sigma-Aldrich, and nisin was extracted using a previously reported method[13]. FDAA-derivatized amino acids (2 μL) were injected onto an Triple TOF 4600 System (AB Sciex) equipped with a Prominence Ultra-Fast Liquid Chromatography (UFLC) system (Shimadzu) and a C18 reverse-phase column (Phenomenex Aeris™ PEPTIDE XB-C18, 2.6 μm, 4.6×150 mm). The elution gradient was solvent A (H$_2$O with 0.1% formic acid) and solvent B (acetonitrile) at a flow rate of 0.2 mL/min over a 35 min period as follows: T = 0 min, 5% B; T = 2 min, 10% B; T = 25 min, 40% B; T = 27 min, 90% B; T = 30 min, 90% B; T = 35 min, 5% B. The mass spectrometer was operated in positive mode with a mass range of 50–1700 *m/z*. The molecular mass corresponding to FDAA-residues was extracted for data analysis.

Peptide Synthesis. Automated peptide synthesis was performed on a CEM Liberty Blue Lite peptide synthesis system (909650) with wang resin or 2-chlorotrityl resin. Peptide synthesis was performed following the general protocol using DMF as solvent, deblocking (5 min × 1) in piperidine/DMF (20: 80, v/v), coupling for 10 min using DIC/Oxyma (1: 1) as coupling reagents. For amino acids after steric hindered residues, the coupling cycle was repeated twice. The synthesized peptide was dissolved in 50 mM HEPE buffer (pH 8.0) and purified on a preparative HPLC system (Shimadzu, Japan) equipped with an ACE 5 C18-300 (10 mm × 250 mm) column.

Measurement of binding affinities by MST. As a representative example, the binding affinity between the purified His$_6$-RosK and RosY (His$_6$-tag free) was measured using the Monolith NT.115 Pico (Nanotemper Technologies). His$_6$-RosK were site-specifically fluorescently labelled according to the protein labeling procedure described in The Monolith His-tag Labeling Kit RED-tris-NTA 2nd Generation. The same volume (10 μL) labelled His$_6$-RosK was mixed thoroughly with same volume unlabeled RosY of 16 different serial concentrations in PBS buffer supplemented with

0.05% Tween 20. The mixture was then loaded into 16 silica capillaries and measured at 25°C using the standard method set on the Monolith NT.115 Pico. Each assay was repeated three times and data analyses were performed using Nanotemper analysis software and Origin 2020 software. Specifically, to measure the binding affinity between the purified RosX and peptides, RosX was fluorescently labelled using the Monolith Protein Labeling Kit RED-NHS 2nd Generation (Amine Reactive), following the standard protein labeling procedure described in the manual.

Cell cytotoxicity and wound scratch assay. Cell cytotoxicity assays were performed according to the methods described in the reference[14]. Cells were divided into four groups and treated with rosin A1 at different concentrations (0 μM, 0.026 μM, 0.052 μM and 0.104 μM). Recombiant human epidermal growth factor (rhEGF) was utilized as the positive control at a concentration of 0.1mg/ml. The cell viability was then measured by a CCK-8 assay after culturing for 24 h. All trials were conducted in triplicate, and the statistical means and standard deviations were calculated.

Scratch experiments were conducted to assess the migratory capacity of HSFs treated with rosin A1. The experimental protocol was adapted from reference[15]. HSFs in the logarithmic growth phase were seeded into 6-well plates at a density of 1.0×10^5 cells per well. After 24 hours of incubation, scratches were introduced into the cell monolayer using a 10 μL pipette tip held perpendicular to the plate, ensuring uniform scratch widths. The wells were then gently washed twice with PBS to remove any detached cells or debris. A serum-free medium containing rosin A1 at concentrations of 0.052 μM and 0.104 μM was added to each well. Cell migration was observed at specific time intervals (2 hours, 6 hours, 18 hours, and 24 hours) using an inverted microscope at 40× magnification. The scratch area was analyzed quantitatively using ImageJ software to determine the extent of migration. All data were analyzed using SPSS 20.0 software. Each experiment was performed in triplicate, and results were expressed as the mean ± standard deviation.

Cell migration rate = (0 h scratch area-scratch area after culture)/(0 h scratch area)×100%

Table S1. Summary of putative class V_a lanthipeptide BGCs lacking methyltransferase genes, including their NCBI RefSeq accession numbers and associated organisms.

NCBI RefSeq Accession	Organism
NC_020504.1	*Streptomyces davaonensis* JCM 4913
NZ_AQUZ01000039.1	*Kribbella catacumbae* DSM 19601
NZ_BAAAGV010000173.1	*Streptomyces thermocarboxydus* JCM 10368
NZ_BAAAHL010000058.1	*Acrocarpospora macrocephala* JCM 10982
NZ_BAAAHV010000027.1	*Amycolatopsis albidoflavus* JCM 11300
NZ_BAAAPC010000014.1	*Nocardiopsis rhodophaea* JCM 15313
NZ_BAAATK010000034.1	*Streptomyces glaucus* JCM 6922
NZ_BBXC01000012.1	*Herbidospora sakaeratensis* NBRC 102641
NZ_BBXD01000019.1	*Herbidospora mongoliensis* NBRC 105882
NZ_BLAE01000047.1	*Acrocarpospora macrocephala* NBRC 16266
NZ_BOOD01000006.1	*Microbispora rosea* NBRC 14044
NZ_CP010849.1	*Streptomyces cyaneogriseus* NMWT 1
NZ_CP022433.1	*Streptomyces pluripotens* MUSC 137
NZ_CP024894.1	*Amycolatopsis* sp. AA4
NZ_CP030862.1	*Streptomyces globosus* LZH-48
NZ_CP041602.2	*Streptomyces* sp. RLB3-6
NZ_CP041604.2	*Streptomyces* sp. S1A1-7
NZ_CP041609.2	*Streptomyces* sp. S1D4-20
NZ_CP041610.2	*Streptomyces* sp. RLB3-17
NZ_CP041611.1	*Streptomyces* sp. S1A1-3
NZ_CP041613.2	*Streptomyces* sp. S1D4-23
NZ_CP041651.1	*Streptomyces* sp. RLB3-5
NZ_CP095011.1	*Streptomyces* sp. 2P-4
NZ_CP107567.1	*Streptomyces peucetius* NA0869
NZ_CP107812.1	*Streptomyces* sp. NBC_00539
NZ_CP107955.1	*Streptomyces mirabilis* NBC_00381
NZ_CP108053.1	*Streptomyces* sp. NBC_00287
NZ_CP108587.1	*Streptomyces* sp. NBC_01185
NZ_CP108707.1	*Streptomyces* sp. NBC_01003
NZ_CP109026.1	*Streptomyces* sp. NBC_00664
NZ_CP109334.1	*Streptomyces decoyicus* NBC_01586
NZ_CP136053.1	*Amycolatopsis* sp. WGS_07
NZ_FTNI01000012.1	*Microbispora rosea* ATCC 12950
NZ_GG657746.1	*Streptomyces* sp. AA4
NZ_JABAQG010000001.1	*Streptomyces* sp. RLA2-12
NZ_JADWYN010000012.1	*Streptomyces* sp. MUM 16J
NZ_JAGGOJ010000007.1	*Streptomyces* sp. ISL-10
NZ_JAGIYX010000014.1	*Kitasatospora* sp. RG8
NZ_JAHCST010000001.1	*Acrocarpospora catenulata* H8750
NZ_JAIUKD010000104.1	*Streptomyces* sp. 7G
NZ_JAKEIP010000330.1	*Streptomyces muensis* DSM 103493
NZ_JAMJFM010000001.1	*Streptomyces* sp. MCA2
NZ_JAMXMV010000010.1	*Actinoallomurus soli* WRP6H-15
NZ_JAPEMK010000001.1	*Streptomyces mirabilis* NBC_00174
NZ_JARAKF010000001.1	*Streptomyces mirabilis* P8-A2
NZ_JARAZH010000003.1	*Streptomyces europaeiscabiei* ID02-12
NZ_JARAZJ010000006.1	*Streptomyces europaeiscabiei* ID01-16c
NZ_JARBAC010000009.1	*Streptomyces europaeiscabiei* AK02-03a
NZ_JAUQWI010000008.1	*Streptomyces* sp. MK5
NZ_JAVACL010000013.1	*Streptomyces* sp. MK7
NZ_JBAGJZ010000009.1	*Streptomyces* sp. PTD5-9
NZ_JBDPLV010000018.1	*Acrocarpospora* sp. B8E8
NZ_JBEORY010000013.1	*Kitasatospora* sp. NPDC097691
NZ_JBEOUD010000066.1	*Streptomyces* sp. NPDC094034
NZ_JBEOXY010000001.1	*Streptomyces* mirabilis NPDC002021
NZ_JBEPAP010000009.1	*Streptomyces* sp. NPDC001508
NZ_JBEPAS010000030.1	*Streptomyces mirabilis* NPDC001349
NZ_JBEPCD010000129.1	*Amycolatopsis* sp. NPDC000746
NZ_JBEPCE010000075.1	*Amycolatopsis* sp. NPDC000740
NZ_JBEPCS010000006.1	*Streptomyces* sp. NPDC000658
NZ_JBEXFF010000001.1	*Streptomyces mirabilis* NPDC005394
NZ_JBEXMB010000025.1	*Streptomyces mirabilis* NPDC004235
NZ_JBEXMH010000004.1	*Amycolatopsis* sp. NPDC004079
NZ_JBEXMT010000026.1	*Nonomuraea* sp. NPDC003804
NZ_JBEXOW010000027.1	*Streptomyces* sp. NPDC006540
NZ_JBEXUQ010000040.1	*Streptomyces mirabilis* NPDC005980
NZ_JBEXXZ010000173.1	*Streptomyces mirabilis* NPDC005755
NZ_JBEYAJ010000004.1	*Streptomyces* sp. NPDC019990
NZ_JBEYFR010000059.1	*Streptomyces mirabilis* NPDC007098
NZ_JBEYVS010000001.1	*Streptomyces* sp. NPDC047028
NZ_JBEZAH010000009.1	*Streptomyces varsoviensis* NPDC045862
NZ_JBEZHI010000002.1	*Streptomyces* sp. NPDC048669
NZ_JBEZIH010000005.1	*Streptomyces* sp. NPDC048565
NZ_JBEZIS010000018.1	*Streptomyces mirabilis* NPDC048539
NZ_JBEZLH010000014.1	*Streptomyces* sp. NPDC048309
NZ_JBEZXQ010000075.1	*Streptomyces aureus* NPDC026676
NZ_JBFAJO010000051.1	*Kitasatospora* sp. NPDC050463
NZ_JBFAZD010000004.1	*Streptomyces* sp. NPDC051662
NZ_JBHJTZ010000087.1	*Streptomyces mirabilis* NPDC056196
NZ_JBHJUK010000186.1	*Kitasatospora* sp. NPDC056181
NZ_JBHJYC010000040.1	*Streptomyces vinaceus* NPDC055980
NZ_JBHJYM010000003.1	*Streptomyces mirabilis* NPDC055963
NZ_JBHTXB010000016.1	*Streptomyces* sp. NPDC056656
NZ_JBHUKQ010000006.1	*Amycolatopsis albidoflavus* CGMCC 4.7638
NZ_JBHVOM010000014.1	*Streptomyces mirabilis* NPDC057905
NZ_JBHVPR010000007.1	*Streptomyces mirabilis* NPDC057952
NZ_JBHVWS010000084.1	*Streptomyces* sp. NPDC058239
NZ_JBHVWZ010000012.1	*Streptomyces* sp. NPDC058246

NZ_JBHVXE010000013.1	*Streptomyces* sp. NPDC058251
NZ_JBHWBX010000005.1	*Streptomyces mirabilis* NPDC059573
NZ_JBHWDB010000260.1	*Streptomyces mirabilis* NPDC059625
NZ_JBHWGG010000068.1	*Streptomyces mirabilis* NPDC059728
NZ_JBHWIX010000070.1	*Streptomyces mirabilis* NPDC059829
NZ_JBHWLN010000005.1	*Streptomyces mirabilis* NPDC059935
NZ_JBHXJC010000001.1	*Streptomyces* sp. NPDC058371
NZ_JBHXMC010000017.1	*Streptomyces* sp. NPDC058466
NZ_JBHXMJ010000010.1	*Streptomyces* sp. NPDC058475
NZ_JBHXSC010000018.1	*Streptomyces* sp. NPDC058674
NZ_JBHXTH010000014.1	*Streptomyces* sp. NPDC127112
NZ_JBHYOD010000022.1	*Streptomyces rochei* NPDC058810
NZ_JBHYPV010000011.1	*Streptomyces* sp. NPDC058755
NZ_JBHYUA010000082.1	*Streptomyces mirabilis* NPDC059487
NZ_JBHYVL010000014.1	*Streptomyces mirabilis* NPDC059444
NZ_JBHYWV010000117.1	*Streptomyces mirabilis* NPDC059388
NZ_JBIAIH010000002.1	*Streptomyces* sp. NPDC005930
NZ_JBIAKD010000007.1	*Streptomyces mirabilis* NPDC005327
NZ_JBIAMM010000016.1	*Streptomyces* sp. NPDC004266
NZ_JBIAPN010000004.1	*Streptomyces* sp. NPDC002952
NZ_JBIAUA010000001.1	*Microbispora rosea* NPDC001803
NZ_JBIAWA010000003.1	*Streptomyces mirabilis* NPDC001473
NZ_JBIAWE010000009.1	*Streptomyces* sp. NPDC001410
NZ_JBIBAD010000010.1	*Streptomyces leeuwenhoekii* NPDC000029
NZ_JBIBGS010000020.1	*Streptomyces* sp. NPDC008222
NZ_JBIBHW010000006.1	*Streptomyces mirabilis* NPDC008109
NZ_JBIBKY010000003.1	*Streptomyces mirabilis* NPDC007894
NZ_JBIBSX010000017.1	*Streptomyces leeuwenhoekii* NPDC015535
NZ_JBIBYH010000038.1	*Streptomyces* sp. NPDC014744
NZ_JBIBZV010000036.1	*Streptomyces* sp. NPDC014622
NZ_JBICVL010000005.1	*Streptomyces mirabilis* NPDC048651
NZ_JBICWA010000007.1	*Streptomyces* sp. NPDC048595
NZ_JBICXT010000009.1	*Streptomyces* sp. NPDC048419
NZ_JBICYN010000011.1	*Streptomyces mirabilis* NPDC048345
NZ_JBIROD010000042.1	*Streptomyces mirabilis* NPDC017141
NZ_JBIRSA010000009.1	*Streptomyces sioyaensis* NPDC020874
NZ_JBIRVC010000012.1	*Streptomyces* sp. NPDC020472
NZ_JBIRWT010000007.1	*Streptomyces mirabilis* NPDC020261
NZ_JBIRZM010000011.1	*Streptomyces* sp. NPDC018833
NZ_JBITHI010000040.1	*Streptomyces* sp. NPDC050523
NZ_JBIUAR010000012.1	*Streptomyces leeuwenhoekii* NPDC052441
NZ_JBIUCE010000020.1	*Streptomyces* sp. NPDC051956
NZ_JBIUSA010000007.1	*Streptomyces mirabilis* NPDC088225
NZ_JBIVAU010000010.1	*Streptomyces* sp. NPDC086835
NZ_JBIVXV010000028.1	*Streptomyces* sp. NPDC090445
NZ_JBIWAU010000019.1	*Streptomyces mirabilis* NPDC098086
NZ_JBIWCN010000002.1	*Streptomyces* sp. NPDC097595
NZ_JBIWFV010000001.1	*Streptomyces* sp. NPDC096176
NZ_JBIWMT010000051.1	*Streptomyces mirabilis* NPDC101778
NZ_JBIWPL010000019.1	*Kitasatospora* sp. NPDC101157
NZ_JBJKTW010000001.1	*Streptomyces* sp. MMS24-I29
NZ_JBJVNO010000040.1	*Streptomyces europaeiscabiei* ZRIMU1328
NZ_JBJWOU010000007.1	*Streptomyces* sp. MCC57
NZ_JOEV01000011.1	*Streptomyces cellulosae* NRRL B-2687
NZ_JUIG01000003.1	*Streptomyces* sp. MUSC 125
NZ_LIQX01000078.1	*Streptomyces ossamyceticus* NRRL B-3822
NZ_MPHV02000001.1	*Streptomyces* sp. WAC00263
NZ_MPHV02000001.1	*Streptomyces* sp. WAC00263
NZ_MUMD01000268.1	*Streptomyces rochei* NRRL B-2410
NZ_OCQN01000001.1	*Streptomyces* sp. OK228
NZ_PQSQ01000050.1	*Streptomyces* sp. Ru73
NZ_QBDT01000001.1	*Streptomyces* sp. L2
NZ_WBOF01000001.1	*Streptomyces kaniharaensis* SF-557

Table S2. Putative functions of proteins encoded in the *ros* gene cluster.

orf	Product size (aa)	Conserved domain	Putative function
rosA1	55	LxmA leader domain family RiPP	Precursor peptide
orf1	81	Hypothetical protein	Unknown function
rosP1	447	Pitrilysin family protein	M16B metallopeptidase structural subunit
rosP2	430	Pitrilysin family protein	M16B metallopeptidase catalytic subunit
rosA2	63	LxmA leader domain family RiPP	Precursor peptide
rosT1	588	ABC transporter ATP-binding protein	Transporter
rosA3	60	LxmA leader domain family RiPP	Precursor peptide
rosX	317	Hypothetical protein	Involving cyclization
rosD	219	Flavoprotein	Cysteine decarboxylase
rosJ	322	LLM class flavin-dependent oxidoreductase	$F_{420}H_2$-dependent oxidoreductase
rosT2	279	ABC transporter permease	Transporter
rosT3	293	ABC transporter ATP-binding protein	Transporter
Orf2	106	Transposase	Transposase
rosY	320	T3SS effector HopA1 family protein	Lyase
rosK	367	Class V lanthionine synthetase subunit LxmK	Ser/Thr kinase

Table S3. Chemical shifts of rosin A1 in CD_3OH or CD_3OD.

Residue	N	H^N	Cα	Hα	Cβ	Hβ	Other
2KT-1			198.6		31.1	2.88, 2.82	6.8(Cγ), 1.06(Hγ*)
Dbu-2	-	-	131.4		124.2	5.90	169.0(C'), 12.0(Cγ), 1.79(Hγ*)
Pro-3	-		63.1	4.43	30.5	2.37, 1.98	174.3(C'), 50.6(Cδ), 3.72(Hδ2), 3.64(Hδ3), 25.9(Cγ), 2.04(Hγ2), 1.96(Hγ3)
Dbu-4	-	8.62	130.8		131.9	6.58	167.3(C'), 12.7(Cγ), 1.78(Hγ*)
Leu-5	116.0	8.01	54.4	4.35	40.4	1.77, 1.62	174.9(C'), 30.3(Cγ), 1.28(Hγ), 23.07(Cδ1), 0.90(Hδ1*), 20.9(Cδ2), 0.87(Hδ2*)
Ser-6	112.2	7.86	54.9	4.58	32.6	2.89, 3.47	172.6(C')
Dbu-7	122.4	9.18	131.8		124.9	6.00	169.7(C'), 12.2(Cγ), 1.84(Hγ*)
Ala-8	124.4	8.99	53.6	4.08	16.6	1.50	177.7(C')
Val-9	116.3	7.68	64.5	3.76	29.9	2.37	175.8(C'), 19.1(Cγ1), 1.04(Hγ1*), 20.3(Cγ2), 1.13(Hγ2*)
Cys-10	119.9	7.69	60.2	4.02	32.0	2.99, 2.95	174.1(C')
Ala-11	120.3	8.22	53.9	3.93	16.1	1.44	176.9(C')
Ala-12	117.2	8.42	50.4	3.97	14.7	1.46	174.2(C')
Ala-13	121.9	8.09	54.0	4.07	16.4	1.52	177.4(C')
Val-14	117.3	7.91	64.8	3.51	30.1	2.18	174.6(C'), 19.2(Cγ1), 0.92(Hγ1*), 20.7(Cγ2), 1.05(Hγ2*)
Ala-15	119.2	8.56	50.2	3.92	14.7	1.42	175.2(C')
Val-16	119.8	8.59	65.7	3.74	30.0	2.38	175.9(C'), 19.2(Cγ1), 1.05(Hγ1*), 20.9(Cγ2), 1.17(Hγ2*)
Dbu-17	121.9	9.48	132.5		127.4	6.24	170.1(C'), 12.3(Cγ), 1.85(Hγ*)
Val-18	120.6	8.54	65.3	3.90	30.0	2.35	175.1(C'), 19.6(Cγ1), 1.06(Hγ1*), 21.1(Cγ2), 1.12(Hγ2*)
Ala-19	122.6	8.31	54.2	4.18	15.8	1.70	177.1(C')
DBB-20	113.6	9.31	57.1	3.47	42.9	3.64	173.1(C'), 20.0(Cγ), 1.29(Hγ*)
Trp-21	123.8	8.19	59.8	4.37	27.7	3.42, 3.51	176.2(C'), 127.8(Nε1), 10.27(Hε1), 124.7(Cδ1), 6.93(Hδ1), 128.4(Cδ2), 137.7(Cε2), 118.6(Cε3), 7.57(Hε3), 109.6(Cγ), 121.8(Cη2), 7.06(Hη2), 111.9(Cξ2), 7.30(Hξ2), 119.3(Cξ3), 6.97(Hξ3)
Tyr-22	117.4	8.77	60.5	3.75	37.4	2.88, 2.63	175.0(C'), 128.5(Cγ), 130.8(Cδ*), 6.61(Hδ*), 115.8(Cε*), 6.56(Hε*), 156.8(Cξ)
Tyr-23	111.1	8.39	56.7	4.50	36.4	2.70, 3.21	174.4(C'), 130.0(Cγ), 131.0(Cδ*), 7.28(Hδ*), 115.7(Cε*), 6.75(Hε*), 157.1(Cξ)
Gly-24	108.0	7.78	45.8	3.91			170.2(C')
TEE-25	134.2	10.70	135.3	7.23	98.8	5.45	

2KT, ketobutyrate; Dbu, Dhb; Ala-6, Ala(S)-6; Cys-10, Ala(S)-10; DBB-20, AviMeCys-20; TEE-25, AviMeCys-25.
Pro-3 exhibits a $\Delta\delta(C\beta–C\gamma)$ of 4.6 ppm, indicating a trans conformation.[16]

Table S4. NMR and refinement statistics for rosin A1 structures

	rosin A1
**NMR distance and dihedral constraints**	
Distance constraints	
Total NOE	489
Intra-residue	140
Inter-residue	
Sequential ($\|i - j\| = 1$)	154
Medium-range ($\|i - j\| < 4$)	187
Long-range ($\|i - j\| > 5$)	8
Hydrogen bonds	26
**Structure statistics**	
Violations (mean and s.d.)	
Distance constraints (Å)	0.033±0.001
Max. distance constraint violation (Å)	0.279
Deviations from idealized geometry	
Bond lengths (Å)	0.003±0.000
Bond angles (º)	0.645±0.011
Impropers (º)	0.279±0.004
Average pairwise r.m.s. deviation** (Å)	
Heavy	0.47±0.15
Backbone	0.14±0.08

Pairwise r.m.s. deviation was calculated for residues 6-25 among 20 refined structures.

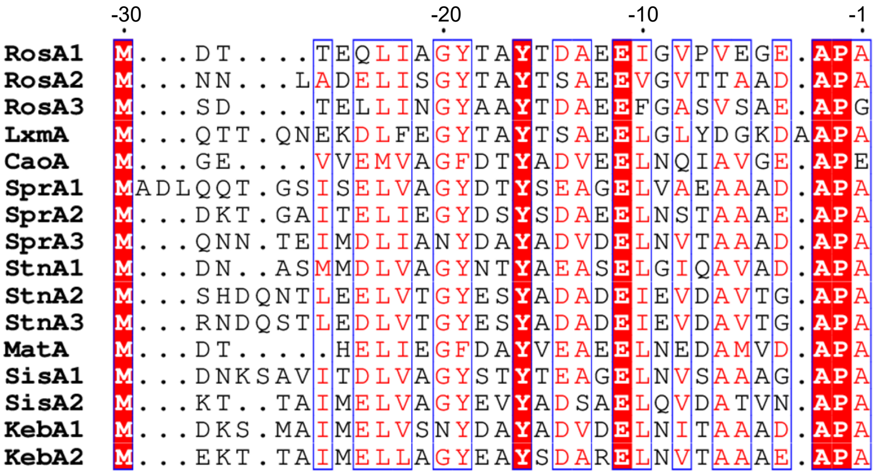

Figure S1. Sequence alignment of class V lanthipeptide leader peptides.

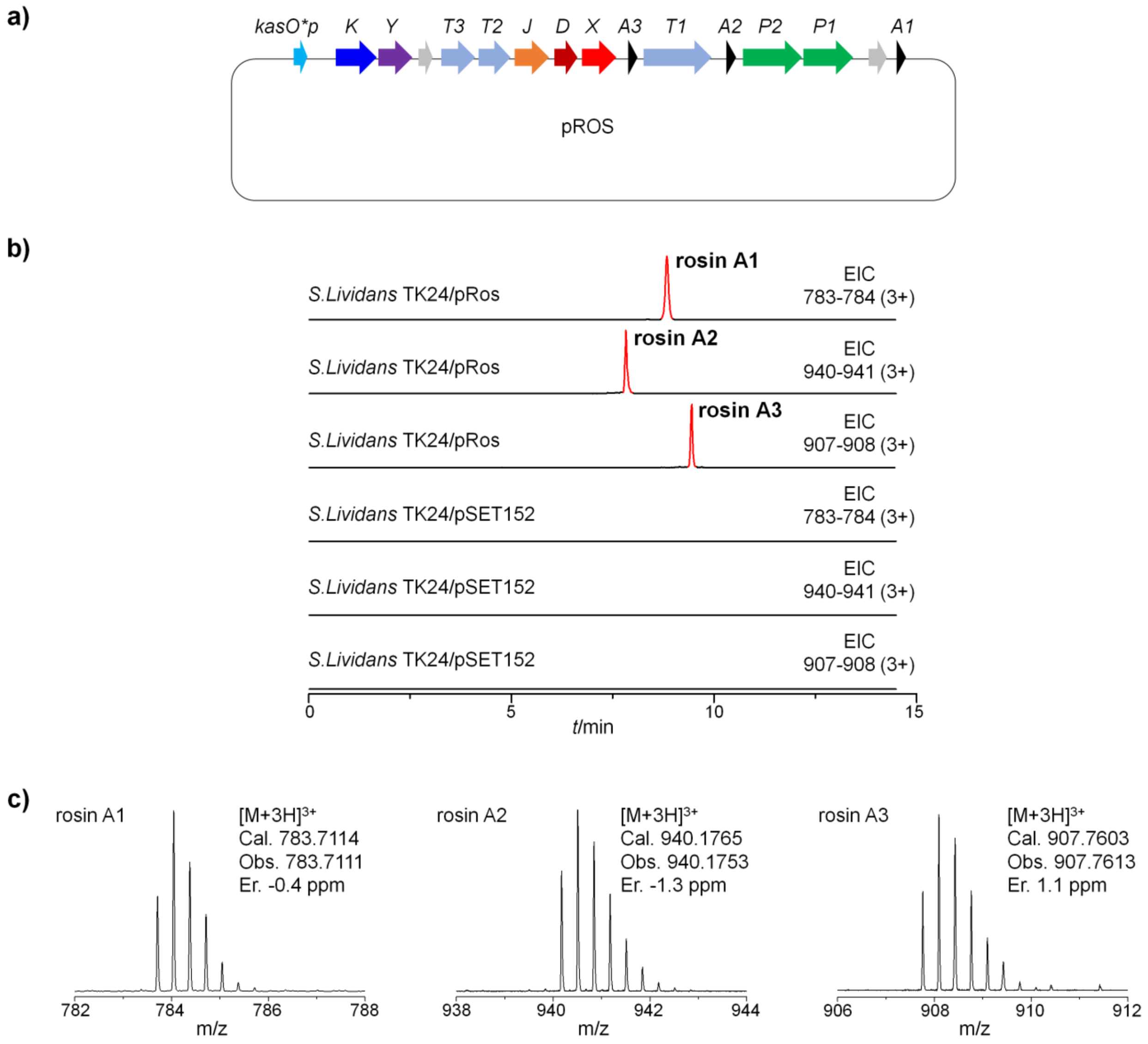

Figure S2. Heterologous expression of the *ros* BGC resulted in the production of rosin A1/A2/A3. (a). The pROS plasmid containing the complete *ros* BGC. (b). LC-MS analysis of the extracts of *S. lividans* TK24/pROS showing the production of rosin A1/A2/A3. *S. lividans* TK24/pSET-152 was employed as a control. (c). Mass spectra of rosin A1/A2/A3.

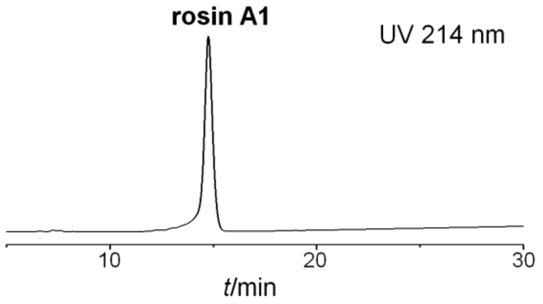

Figure S3. HPLC analysis of the purified rosin A1 sample. The HPLC analysis was performed on an Ultimate Polar RP column (250 × 4.6 mm, 5 μm, Welch Technology Co., Ltd., Shanghai) by gradient elution of solvent A (H_2O with 0.1% formic acid) and solvent B (acetonitrile with 0.1% formic acid) with a flow rate of 1.0 mL/min over a 35 min period as follows: T = 0 min, 50% B; T = 5 min, 50% B; T = 25 min, 90% B; T = 27 min, 99% B; T = 35 min, 99% B. The retention time of rosin A1 was 14.7 min. The spectra was monitored at 214 nm.

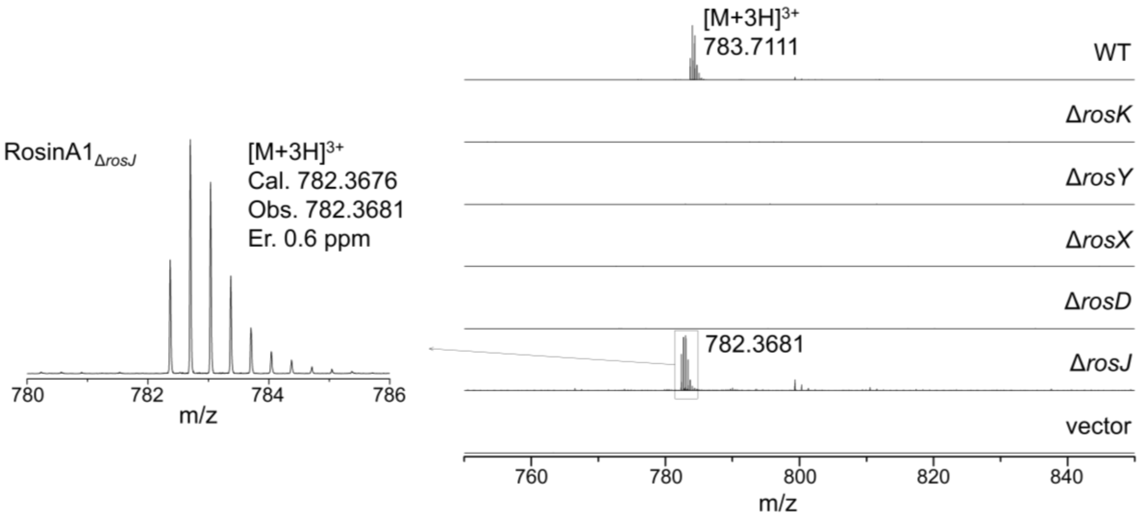

Figure S4. Metabolic analysis of gene disruption mutants. *S. lividans* TK24/pROS (WT) and *S. lividans* TK24/pSET-152 (empty vector) were used as controls.

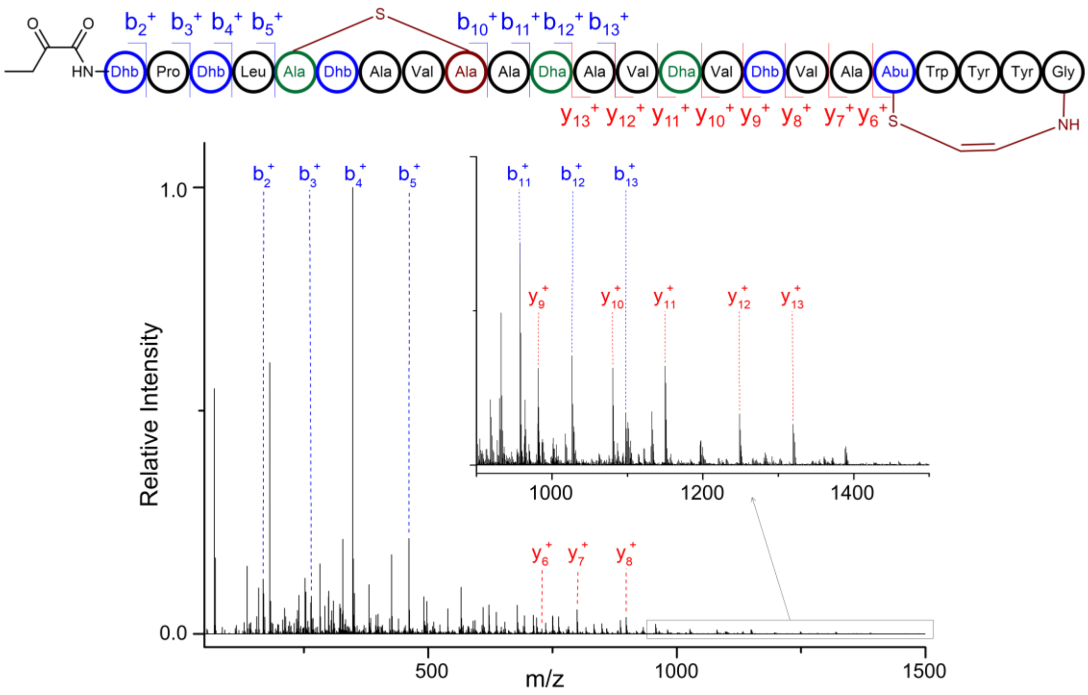

Ions	calc.	obs.	Er. (ppm)	Ions	calc.	obs.	Er. (ppm)
b_2^+	168.0656	168.0656	0	y_6^+	728.2861	728.2881	2.7
b_3^+	265.1183	265.1188	1.9	y_7^+	799.3233	799.3257	3.0
b_4^+	348.1555	348.1554	-0.3	y_8^+	898.3917	898.3950	3.7
b_5^+	461.2395	461.2400	1.1	y_9^+	981.4288	981.4320	3.3
b_{10}^+	886.4128	886.4161	3.7	y_{10}^+	1080.4972	1080.5020	4.4
b_{11}^+	957.4499	957.4531	3.3	y_{11}^+	1149.5187	1149.5230	3.7
b_{12}^+	1026.4714	1026.4755	4.0	y_{12}^+	1248.5871	1248.5904	2.6
b_{13}^+	1097.5085	1097.5132	4.3	y_{13}^+	1319.6242	1319.6307	4.9

Figure S5. LC-MS/MS analysis of rosin A1$_{\Delta rosJ}$. The *b* and *y* ions are listed in table and marked in the spectrum.

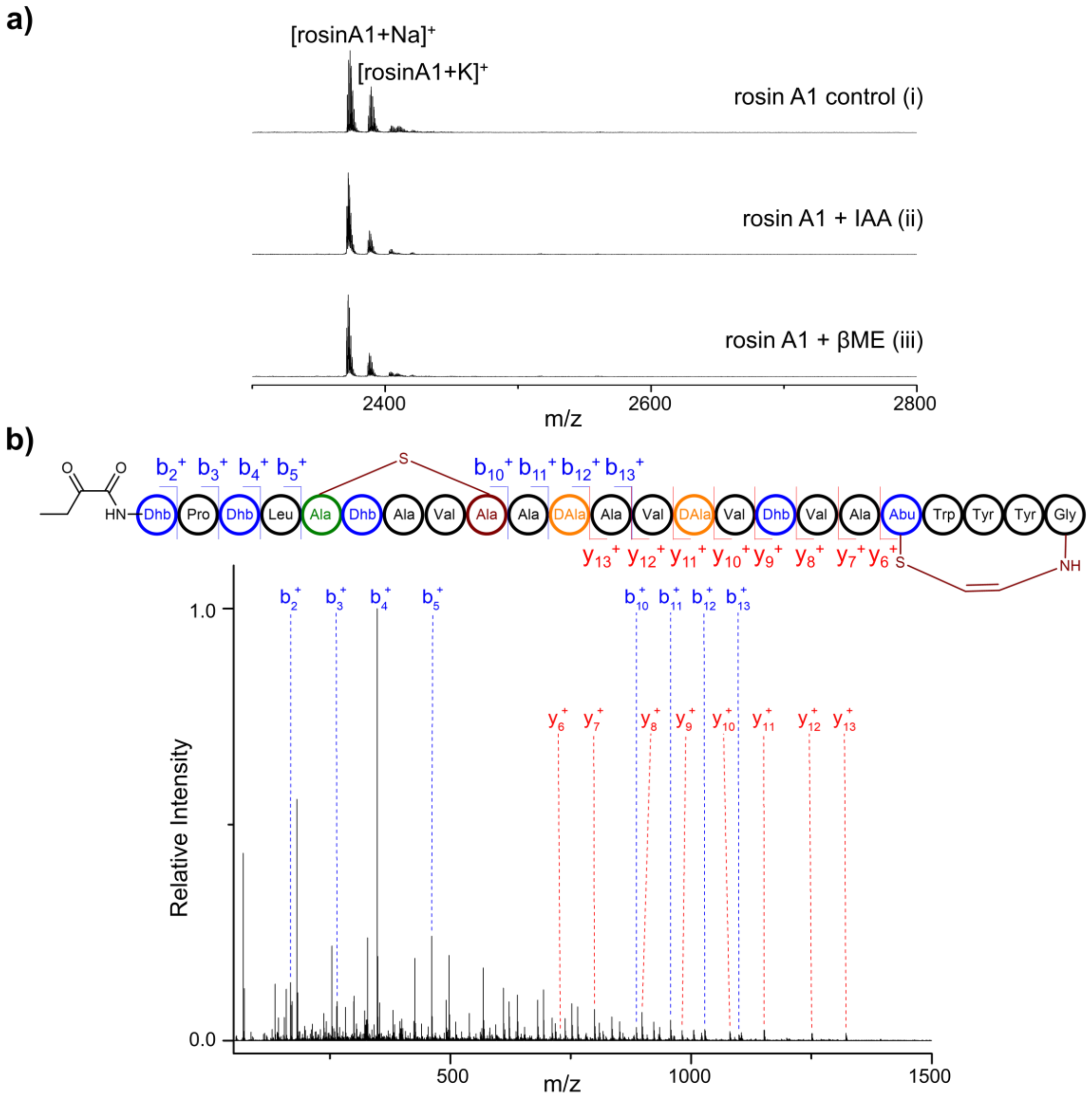

Ions	calc.	obs.	Er. (ppm)	Ions	calc.	obs.	Er. (ppm)
b_2^+	168.0656	168.0655	-0.6	y_6^+	728.2861	728.2873	1.6
b_3^+	265.1183	265.1183	0	y_7^+	799.3233	799.3230	-0.4
b_4^+	348.1555	348.1551	-1.1	y_8^+	898.3917	898.3904	-1.4
b_5^+	461.2395	461.2388	-1.5	y_9^+	981.4288	981.4291	0.3
b_{10}^+	886.4128	886.4141	1.5	y_{10}^+	1080.4972	1080.4976	0.4
b_{11}^+	957.4499	957.4514	1.6	y_{11}^+	1151.5343	1151.5368	2.2
b_{12}^+	1028.4870	1028.4865	-0.5	y_{12}^+	1250.6027	1250.6030	0.2
b_{13}^+	1099.5242	1099.5261	1.7	y_{13}^+	1321.6398	1321.6398	0

Figure S6. MS analysis of rosin A1. (a). MALDI-TOF-MS analysis of rosin A1 treated with IAA and β-ME. Assay conditions: (i) rosin A1 was incubated in 20 mM Tris-HCl, pH 8.0, for 1 hour at room temperature. $[rosinA1+Na]^+$: $M_{obs.}$ = 2371.53 Da, $M_{calc.}$ = 2371.10 Da. $[rosinA1+K]^+$: $M_{obs.}$ = 2387.52 Da, $M_{calc.}$ = 2387.08 Da. (ii) rosin A1 was incubated in 20 mM Tris-HCl, pH 8.0, 0.5 mM TCEP, with 1 mM IAA for 30 min at room temperature. (iii) rosin A1 was incubated in 20 mM Tris-HCl, pH 8.0, with 0.5 mM βME for 1 hour at 37°C; (b). LC-MS/MS analysis of rosin A1. The *b* and *y* ions are listed in table and marked in the spectrum.

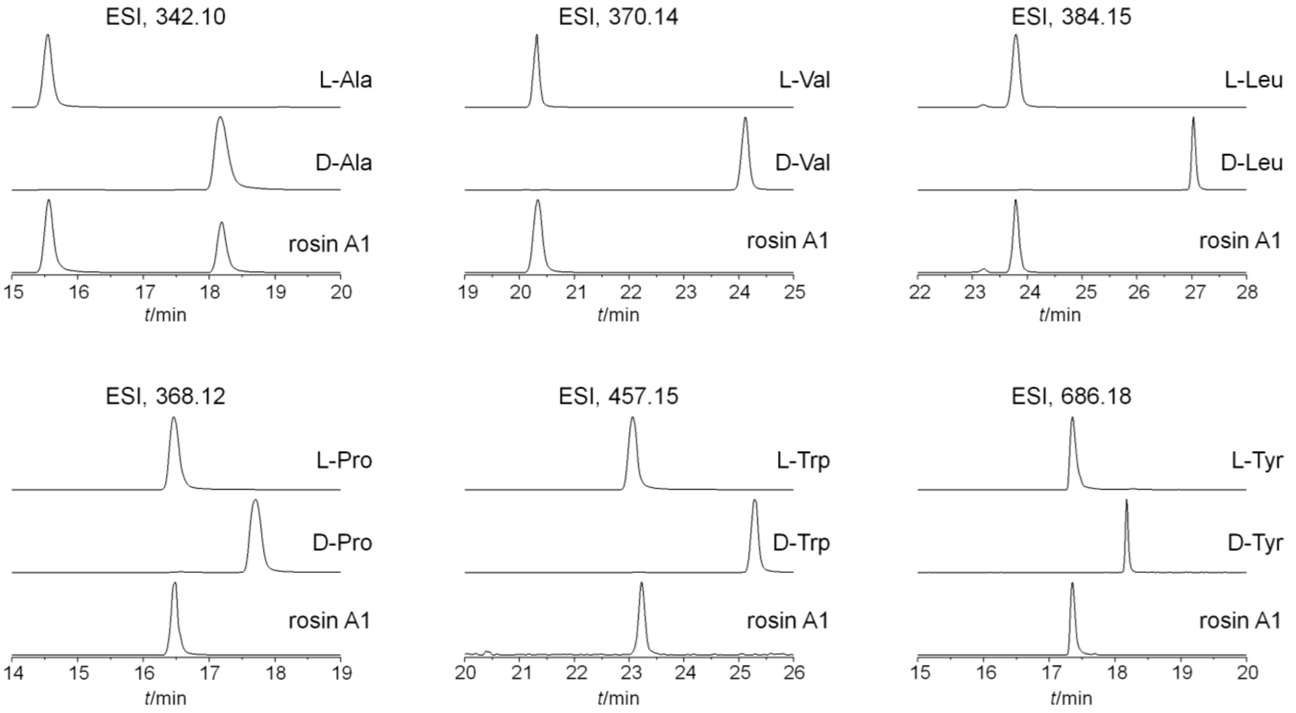

Figure S7. Marfey's analysis of rosin A1. The peak area ratio of L-FDAA-L-Ala / L-FDAA-D-Ala (65/35) is close to the ratio of 2 L-Ala / 1 D-Ala. Tyrosine derivatives are reported as bis-FDAA adducts.

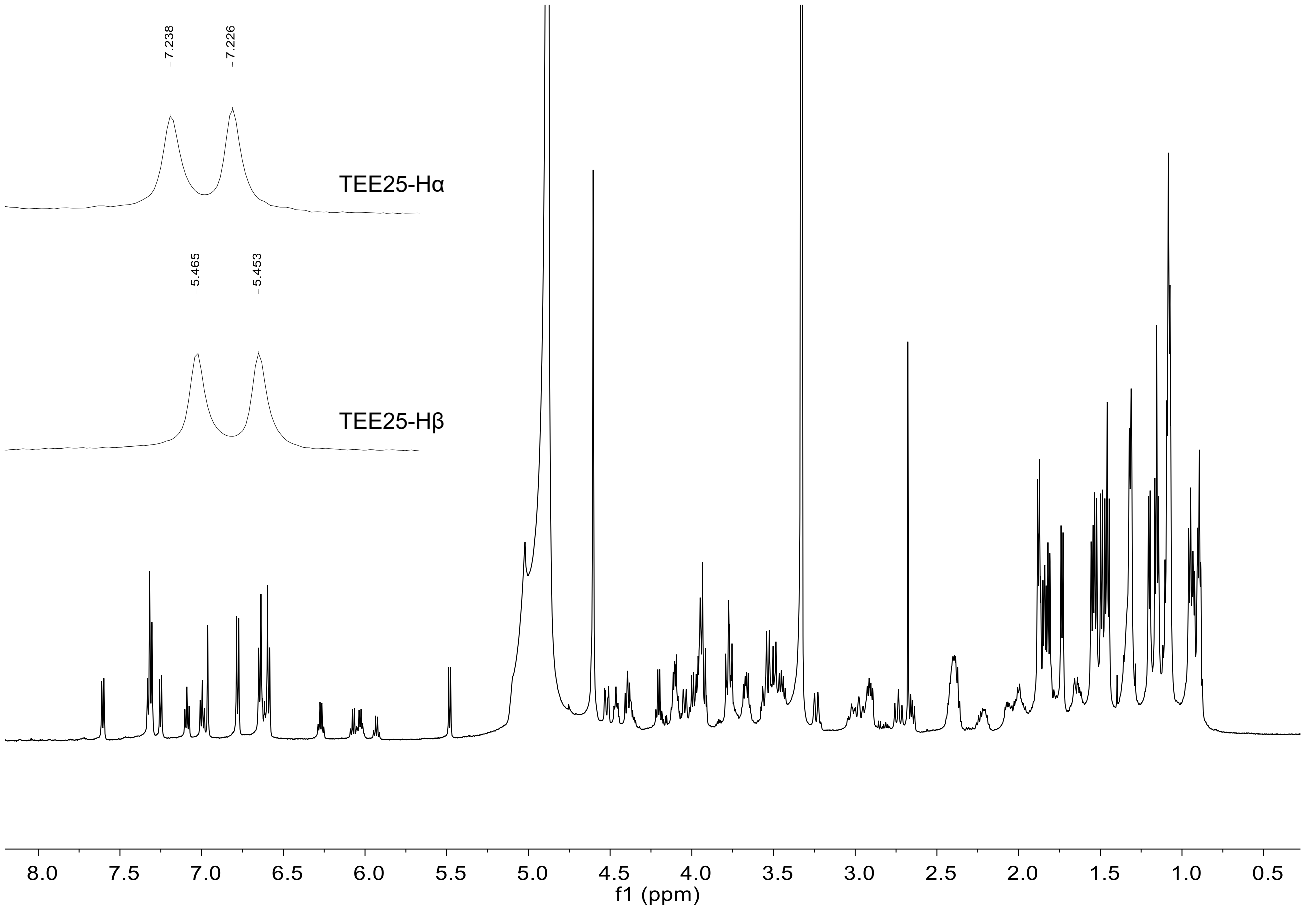

Figure S8. ^{1}H NMR spectrum of rosin A1 in CD_3OD measured at 298 K on Bruker AVANCE III 600 MHz spectrometer.

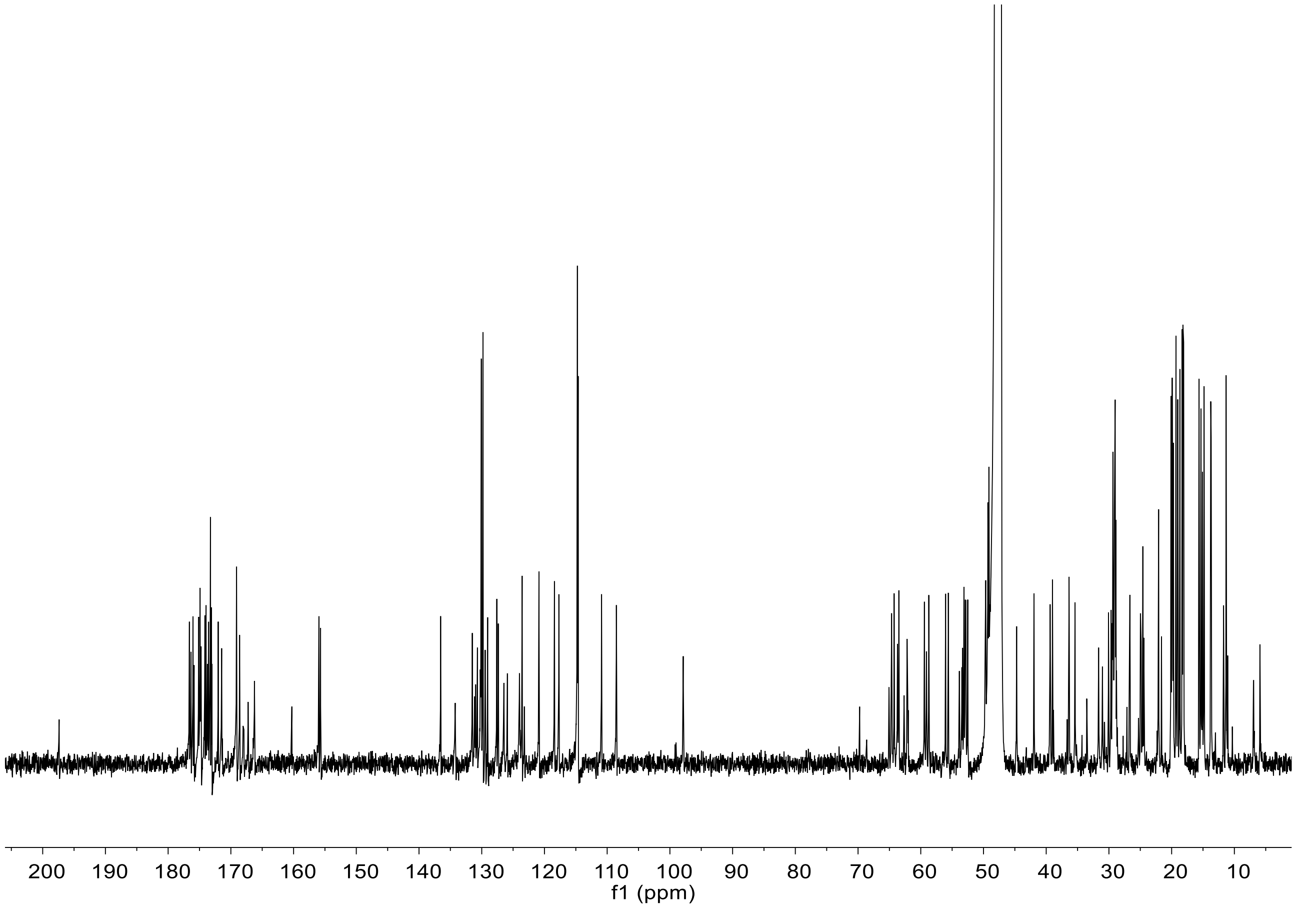

Figure S9. ^{13}C NMR spectrum of rosin A1 in CD_3OD measured at 298 K on Bruker AVANCE III 600 MHz spectrometer.

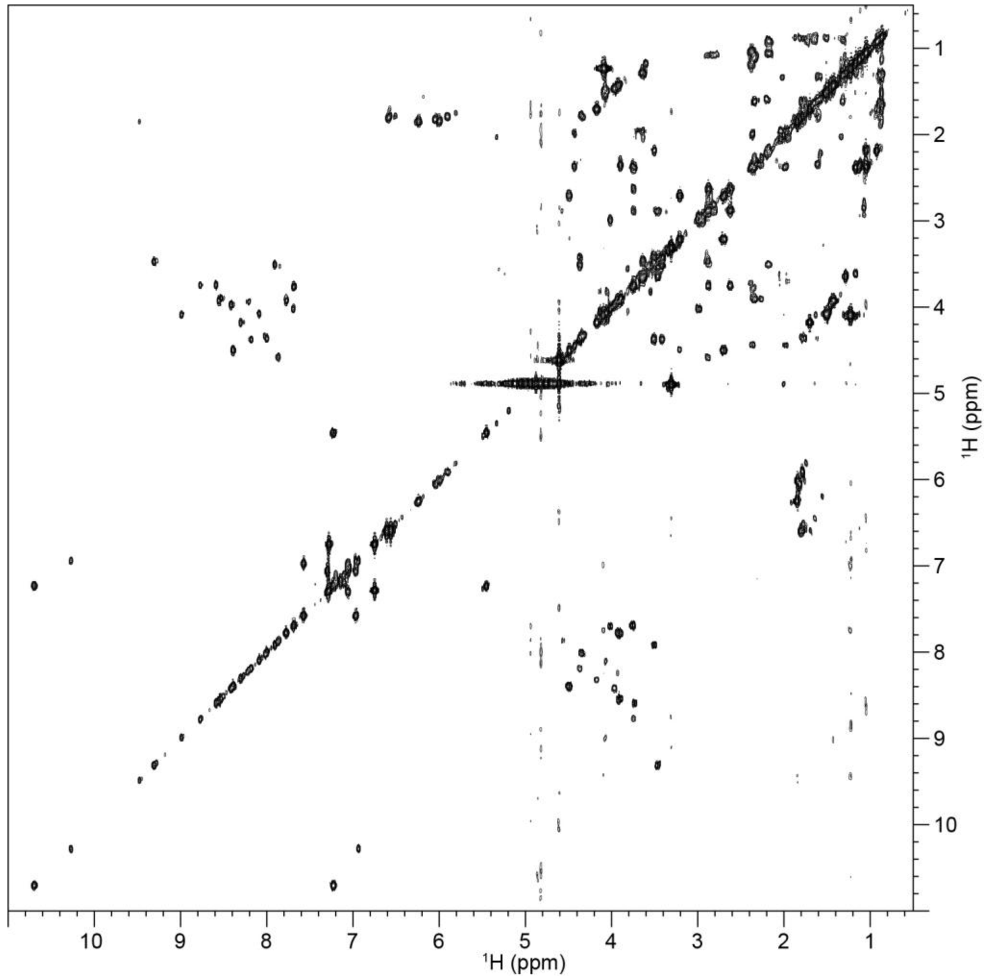

Figure S10. 2D ^1H-^1H COSY spectrum of rosin A1 in CD_3OH measured at 298 K on Bruker AVANCE NEO 800 MHz spectrometer.

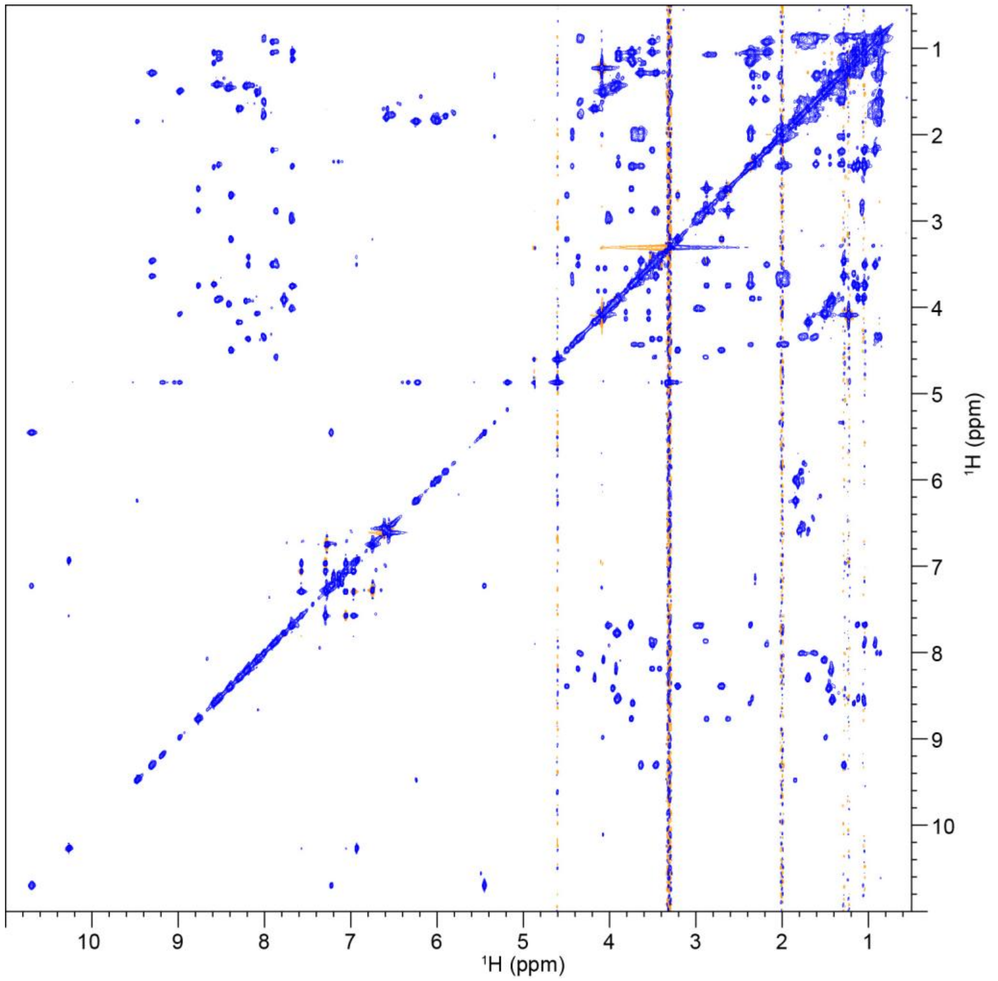

Figure S11. 2D ^1H-^1H TOCSY spectrum (mixing time 80 ms) of rosin A1 in CD_3OH measured at 298 K on Bruker AVANCE NEO 800 MHz spectrometer.

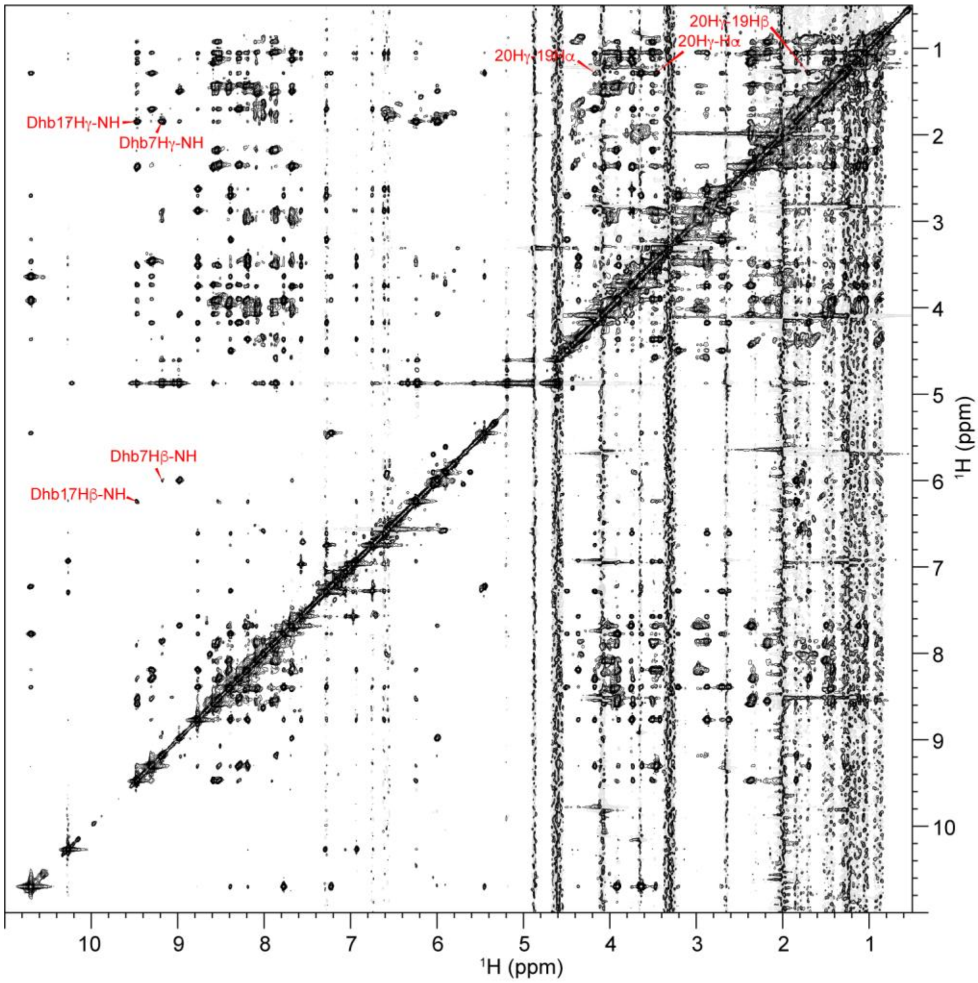

Figure S12. 2D ^1H-^1H NOESY spectrum (mixing time 300 ms) of rosin A1 in CD_3OH measured at 298 K on Bruker AVANCE NEO 800 MHz spectrometer. The *Z*-geometry of Dhb residues was assigned based on the significantly stronger NOE cross peak between NH and Hγ compared to that between NH and Hβ.

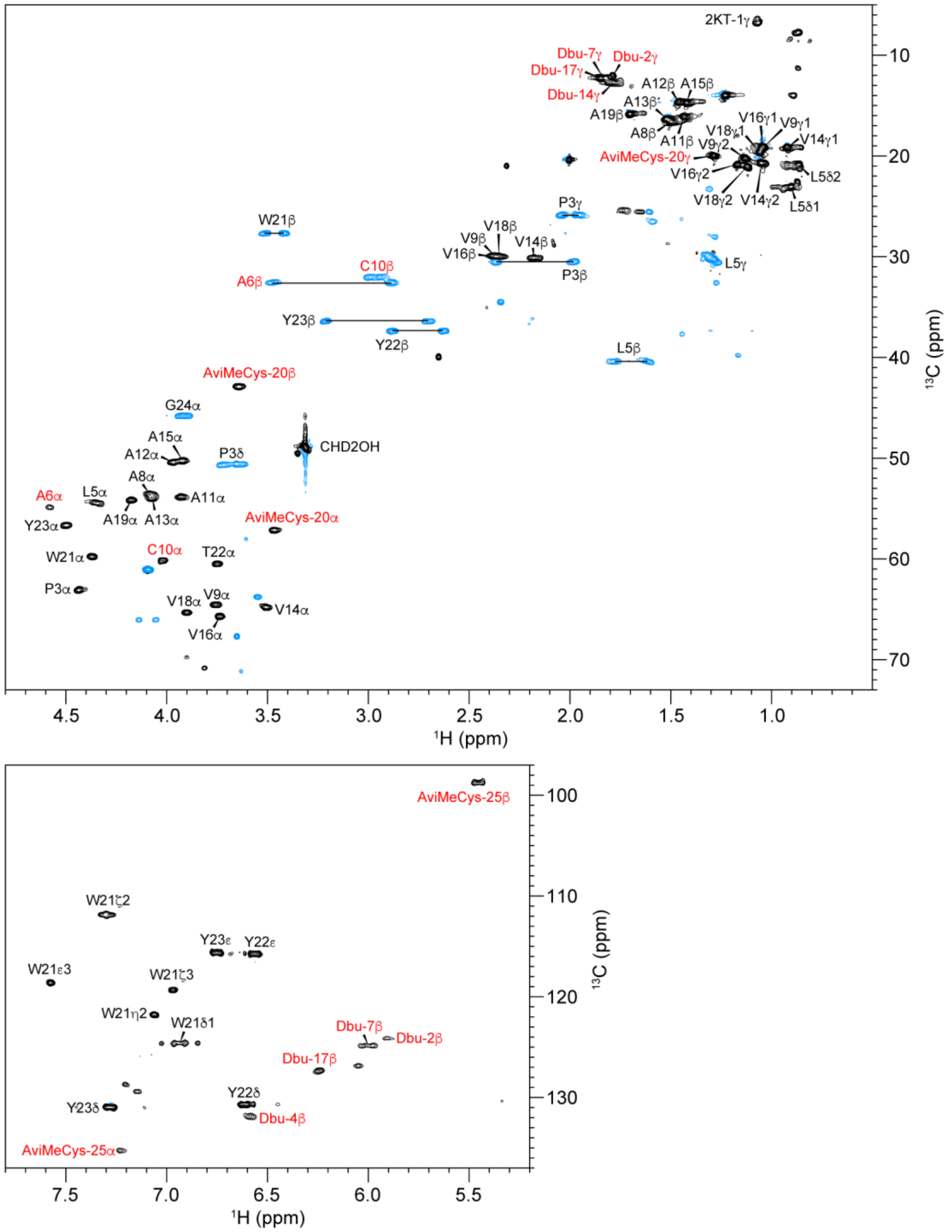

Figure S13. 2D ^{1}H-^{13}C edited HSQC spectrum of rosin A1 in CD_3OH, with opposite phases for CH_2 (black) and CH/CH_3 (blue) signals, measured at 298 K on Bruker AVANCE NEO 800 MHz spectrometer. The Lan and AviMeCys crosslink residues, as well as the Dhb (Dbu) residues, were assigned and labeled in red.

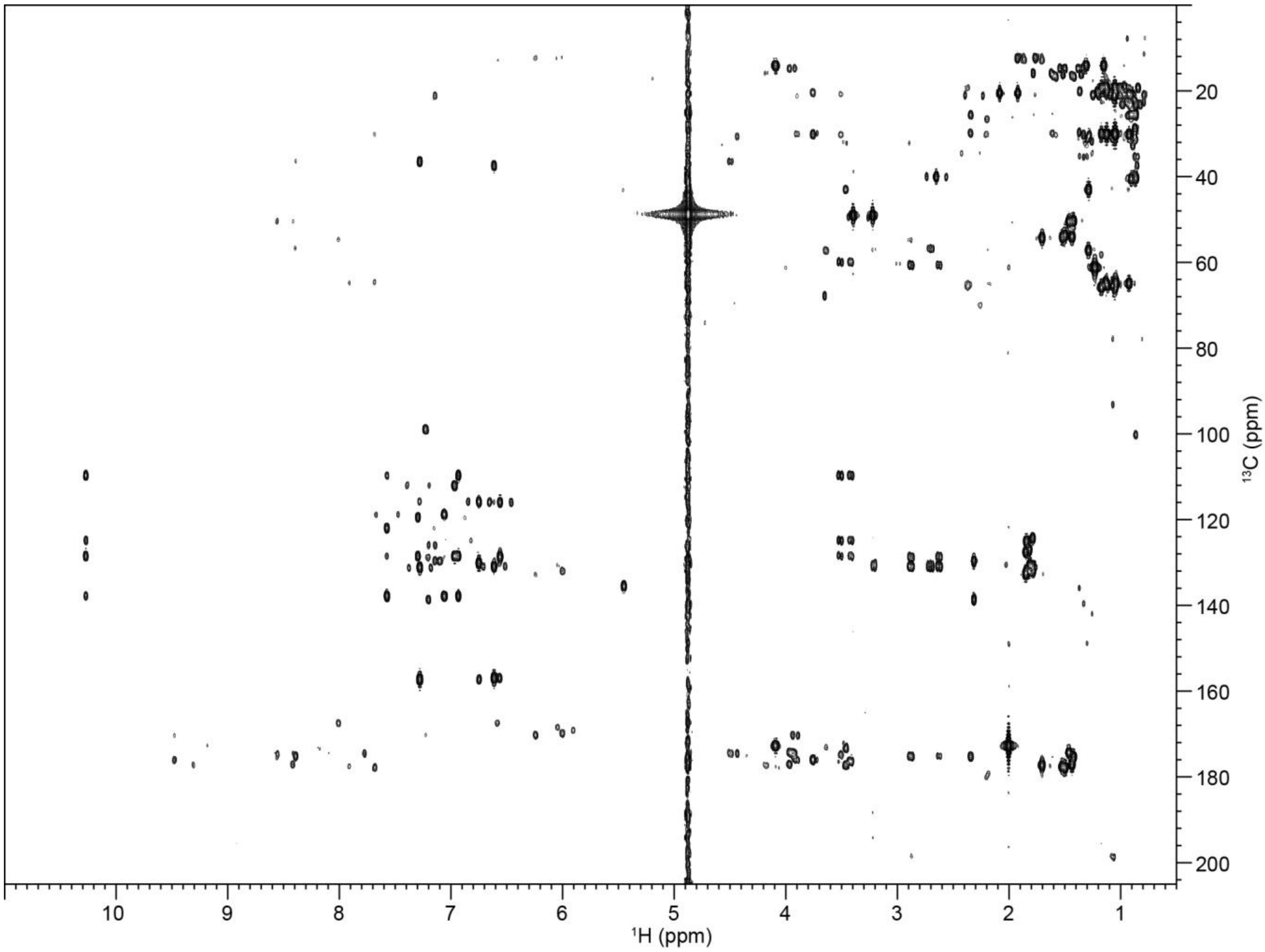

Figure S14. 2D ^{1}H-^{13}C HMBC spectrum of rosin A1 in CD_3OH measured at 298 K on Bruker AVANCE NEO 800 MHz spectrometer.

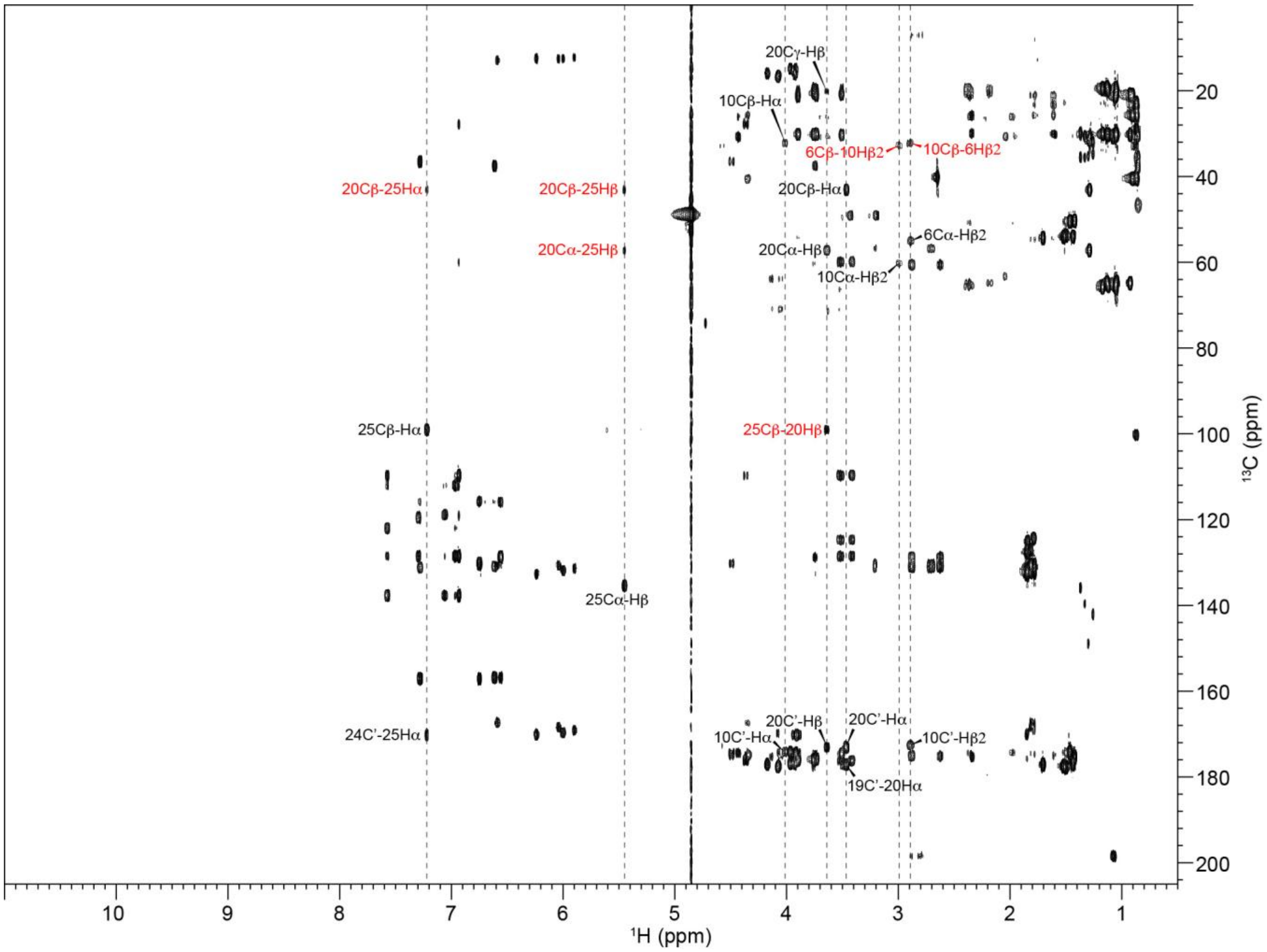

Figure S15. 2D ^{1}H-^{13}C HMBC spectrum of rosin A1 in CD_3OD measured at 298 K on Bruker AVANCE III 600 spectrometer. The heteronuclear cross-peaks were observed between the 6th and 10th residues, and between the 20th and 25th residues, and were labeled in red.

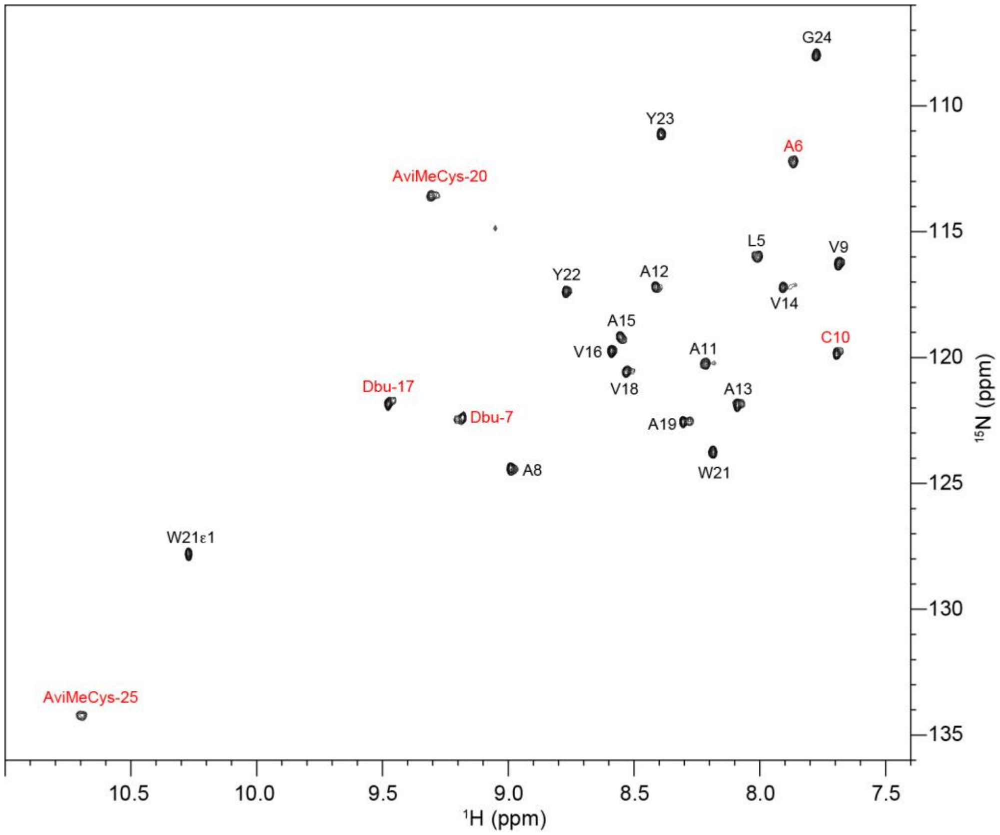

Figure S16. 2D ^{1}H-^{15}N HSQC spectrum of rosin A1 in CD_3OD measured at 298 K on Bruker AVANCE NEO 800 MHz spectrometer.

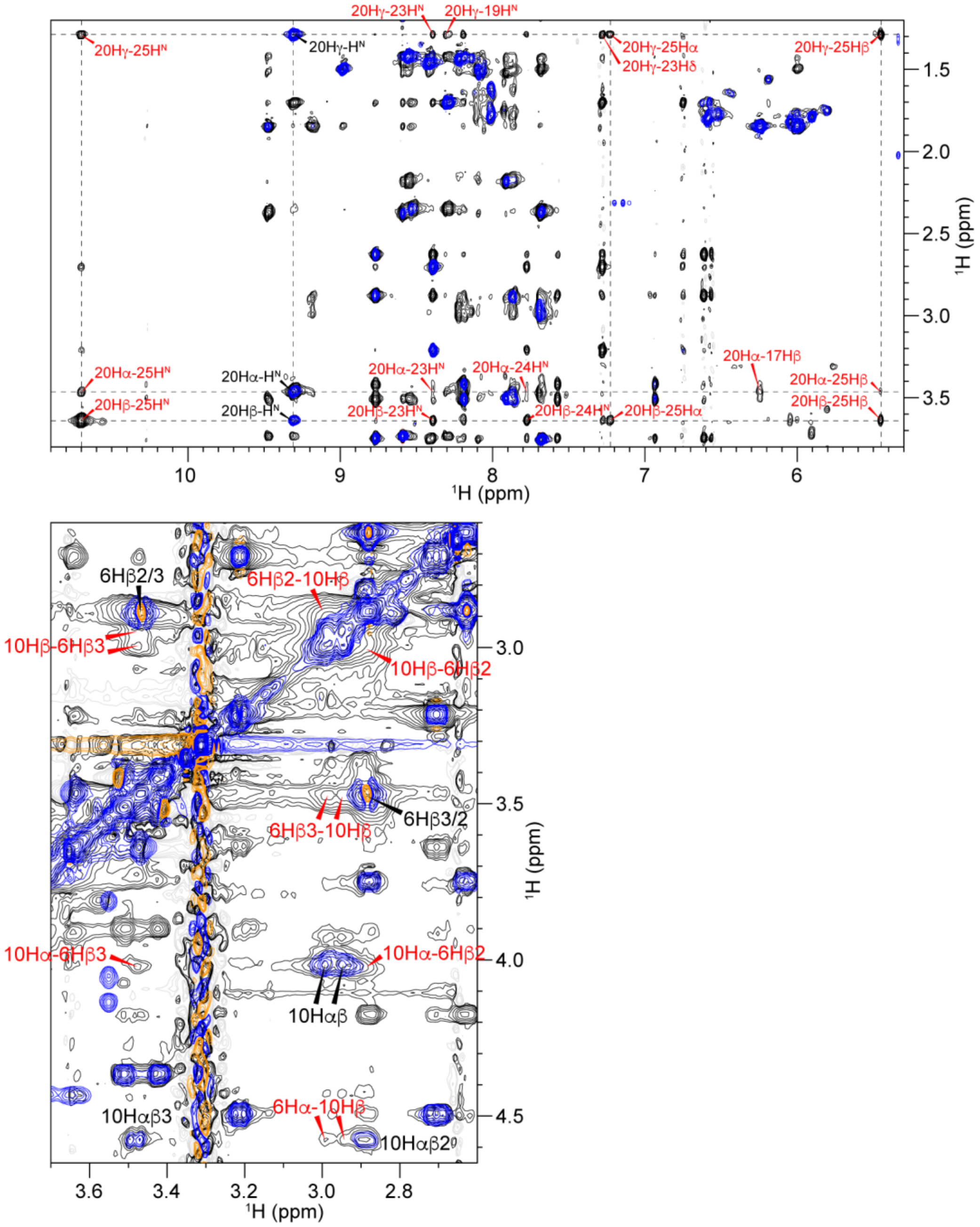

Figure S17. Overlay of 2D ^1H-^1H NOESY (black) and TOCSY (blue) spectra of rosin A1 in CD_3OH at 298 K. Two regions of the spectra were enlarged for clearer comparison. Abundant cross-peaks were observed between the 6th and 10th residues, and between the 20th and 25th residues, in the NOESY spectrum.

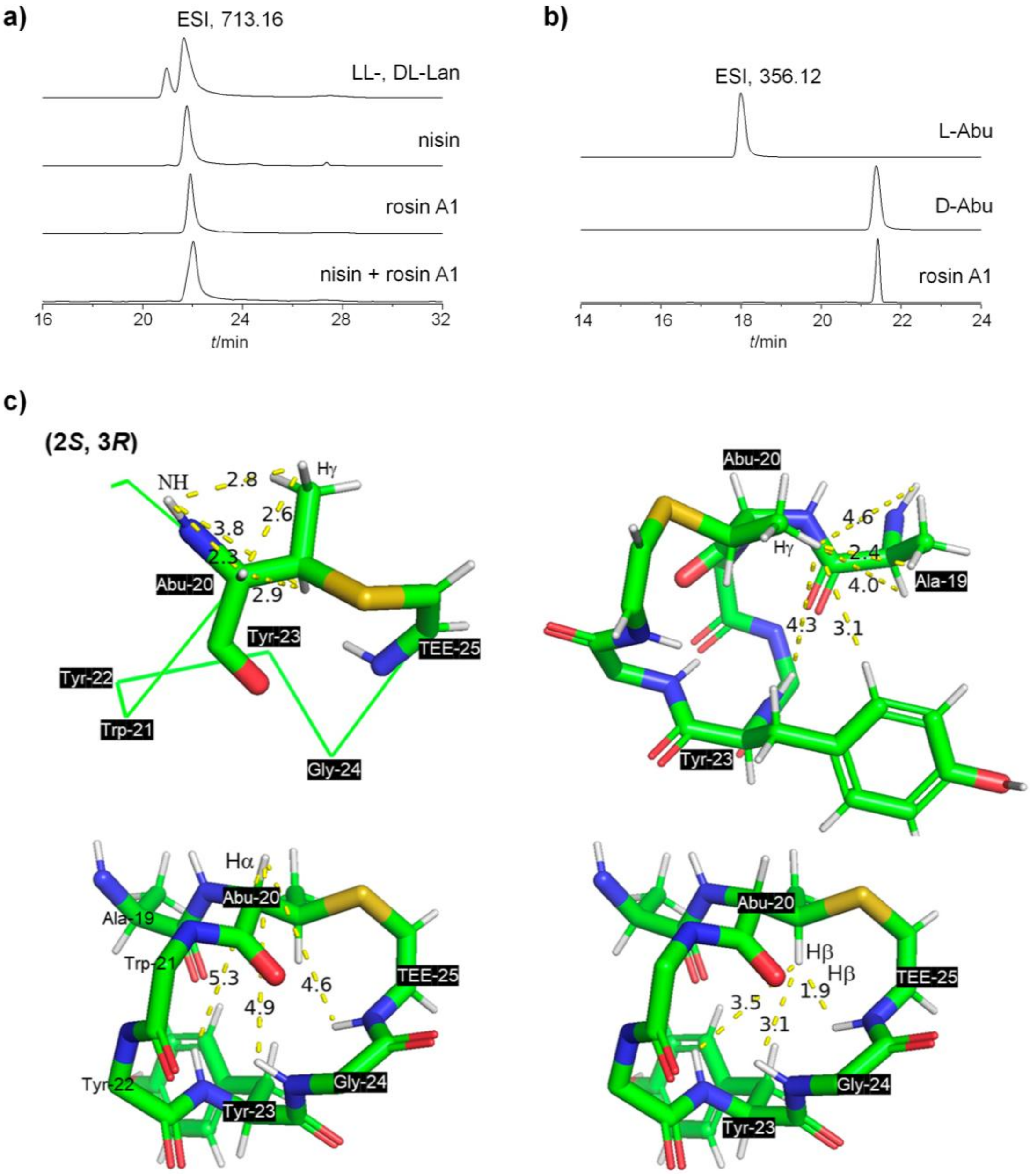

Figure S18. (a). Marfey's analysis of rosin A1 to confirm the configuration of the (2*S*, 6*R*)-Lan; (b). Marfey's analysis of rosin A1 to confirm the '*S*' configuration of the α-C of the AviMeCys. (c). Schematic representation of the (2*S*,3*R*) configuration of Abu-20, highlighting characteristic intra- and inter-residue ^1H–^1H distances.

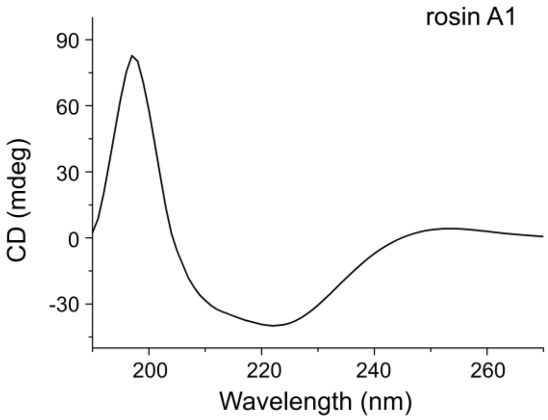

Figure S19. The circular dichroism spectrum of rosin A1 in MeOH at 298 K.

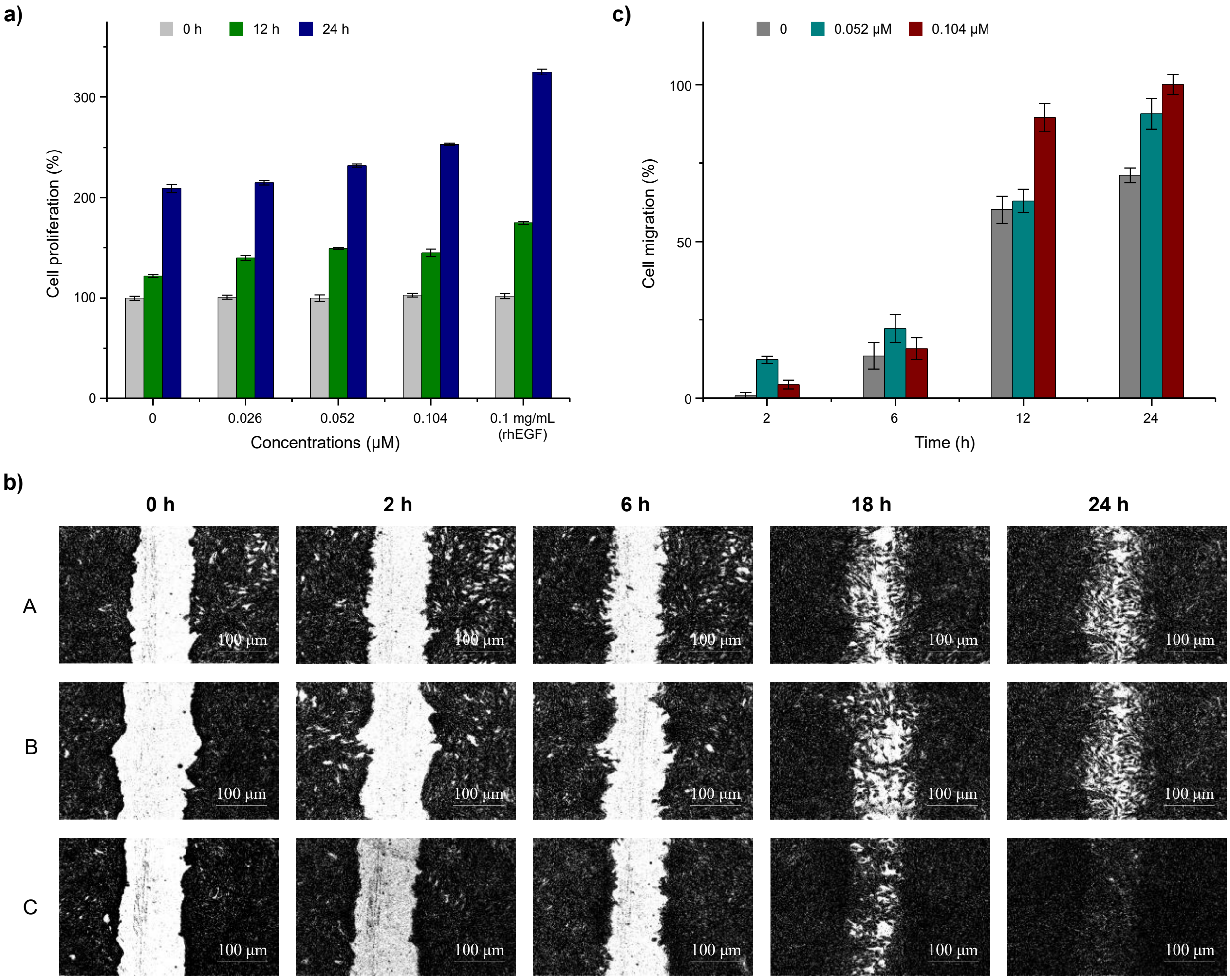

Figure S20. Rosin A1 promotes cell proliferation of HSF cells.
(a). Proliferation trend of HSF induced by rosin A1. rhEGF was used as a positive control.
(b). Wound-healing assay of HSF induced by rosin A1. (A. blank, B. 0.052 μM rosin A1, C. 0.104 μM rosin A1).
(c). Migration of HSF induced by rosin A1.

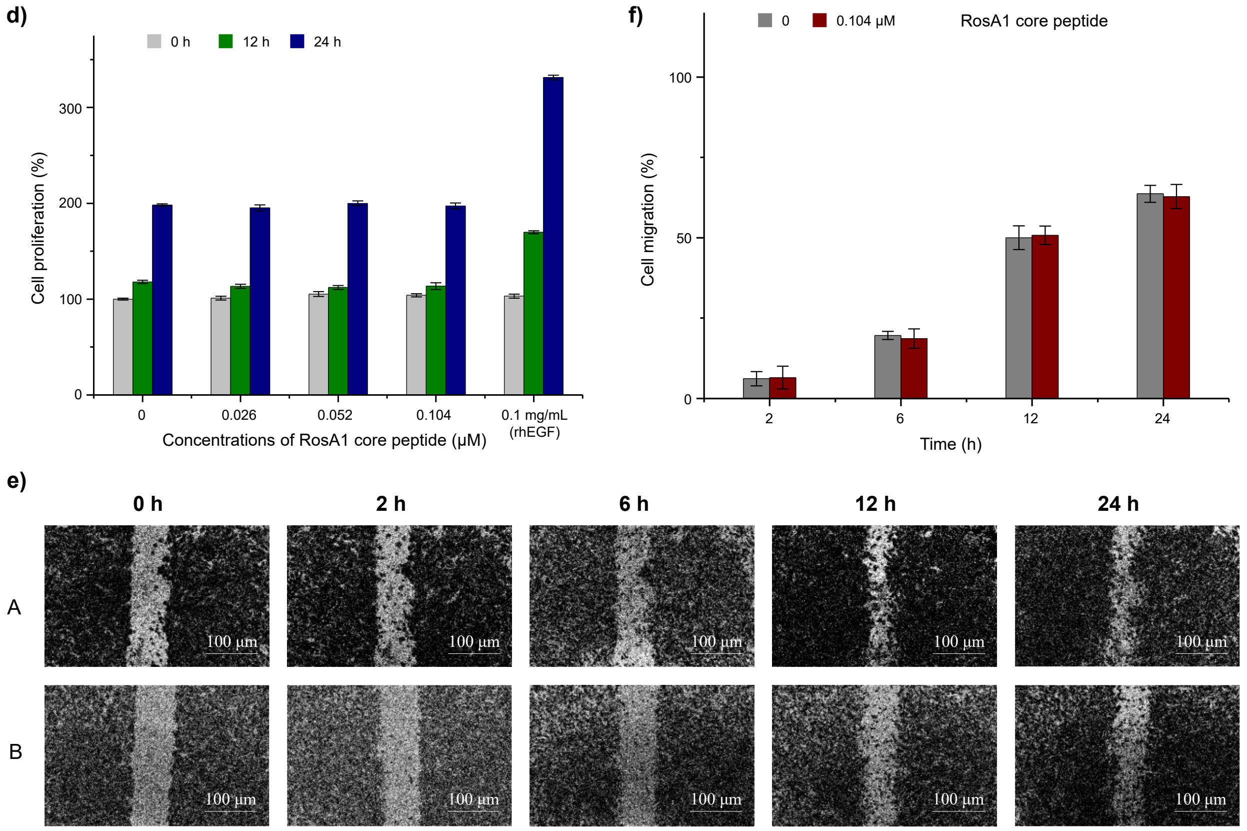

Figure S20.
(d). Proliferation trend of HSF induced by RosA1 core peptide. rhEGF was used as a positive control.
(e). Wound-healing assay of HSF induced by RosA1 core peptide. (A. blank, B. 0.104 μM RosA1 core peptide).
(f). Migration of HSF induced by RosA1 core peptide.

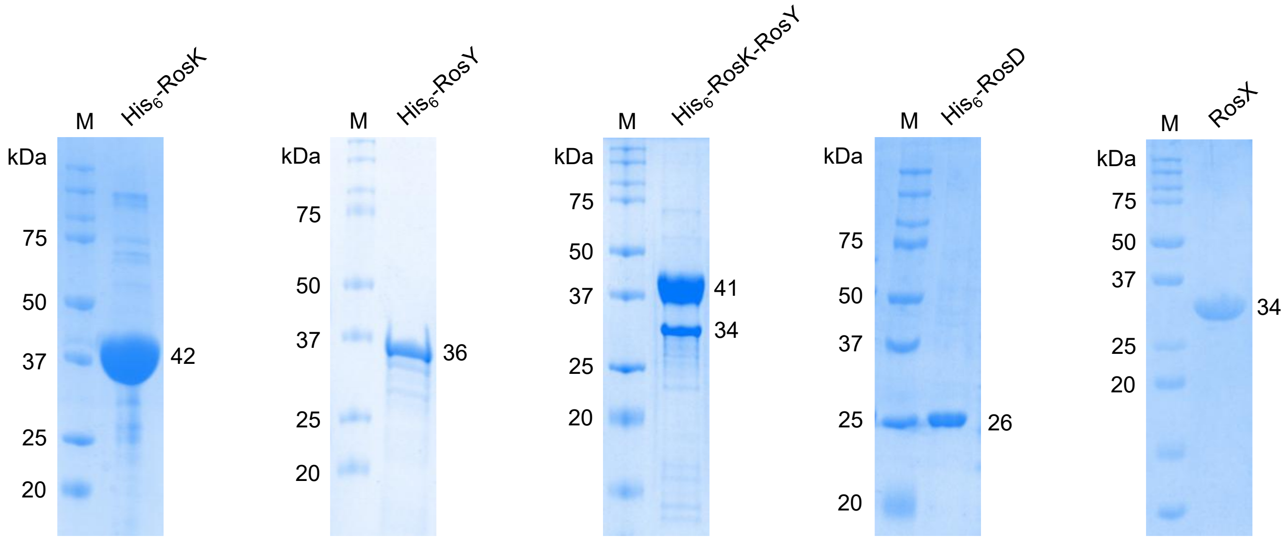

Figure S21. SDS-PAGE analysis of His$_6$-RosK, His$_6$-RosY, His$_6$-RosK-RosY, His$_6$-RosD, and RosX. Protein purity was assessed visually using Coomassie staining.

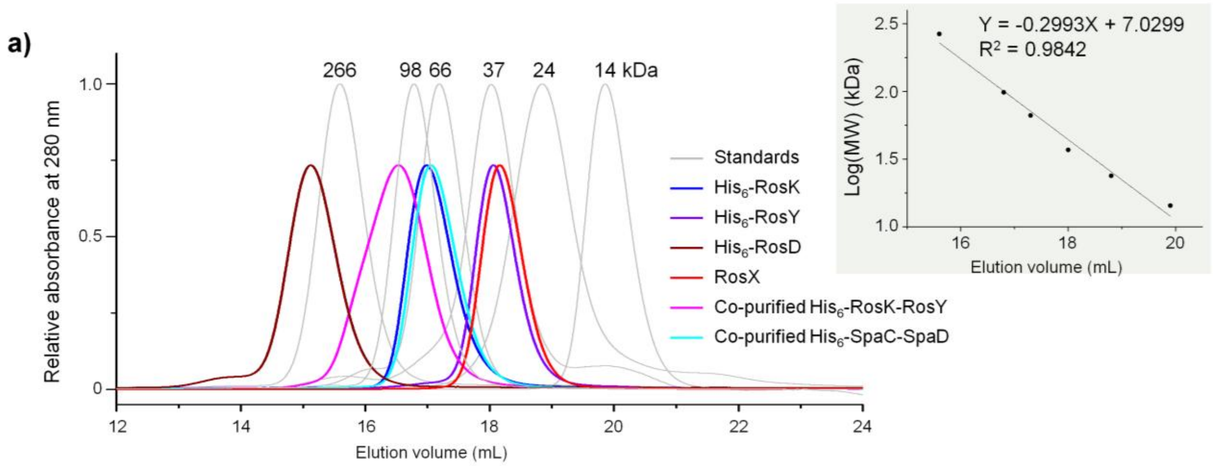

Proteins	Calculated MW (kDa)	Observed MW (kDa)	Putative forms
His_6-RosK	42	87	Dimer
His_6-RosY	36	40	Monomer
Co-purified His_6-RosK-RosY	41 + 34	121	Trimeric RosK-RosY in 2:1 ratio
Co-purified His_6-SpaC-SpaD	41 + 37	81	Heterodimers
His_6-RosD	26	312	Dodecamer
RosX	34	38	Monomer

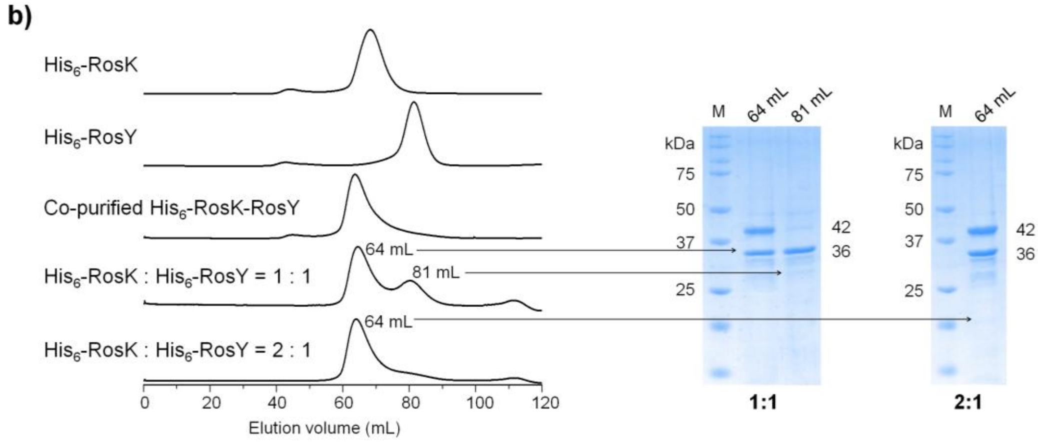

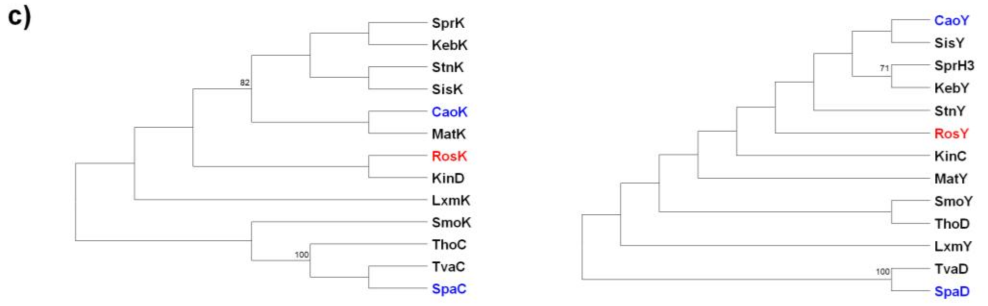

Figure S22. (a). SEC analysis using a Superose™ 6 Increase 10/300 GL column with the following proteins: His_6-RosK (42 kDa), His_6-RosY (36 kDa), co-purified His_6-RosK-RosY (41 + 34 kDa), co-purified His_6-SpaC-SpaD (41 + 37 kDa), His_6-RosD (26 kDa) and RosX (34 kDa). The following standards were used for molecular weight calibration: TvaF (266 kDa),[17] His_6-EryP (98 kDa),[18] bovine serum albumin (BSA, 66 kDa), thrombin (37 kDa), trypsin (24 kDa) and lysozyme (14 kDa). (b). The SEC profiles using a HiLoad™ 16/600 column packed with SuperDex 200 resin to analyze mixed samples of His_6-RosK and His_6-RosY at different ratios and the SDS-PAGE grayscale analysis of the corresponding signals. (c). The maximum likelihood phylogenetic analysis of RosK/RosY in comparison with other LanK/LanY involved in the biosynthesis of class V lanthipeptides and thioamitides. Only bootstrap values greater than 70 are shown on the trees.

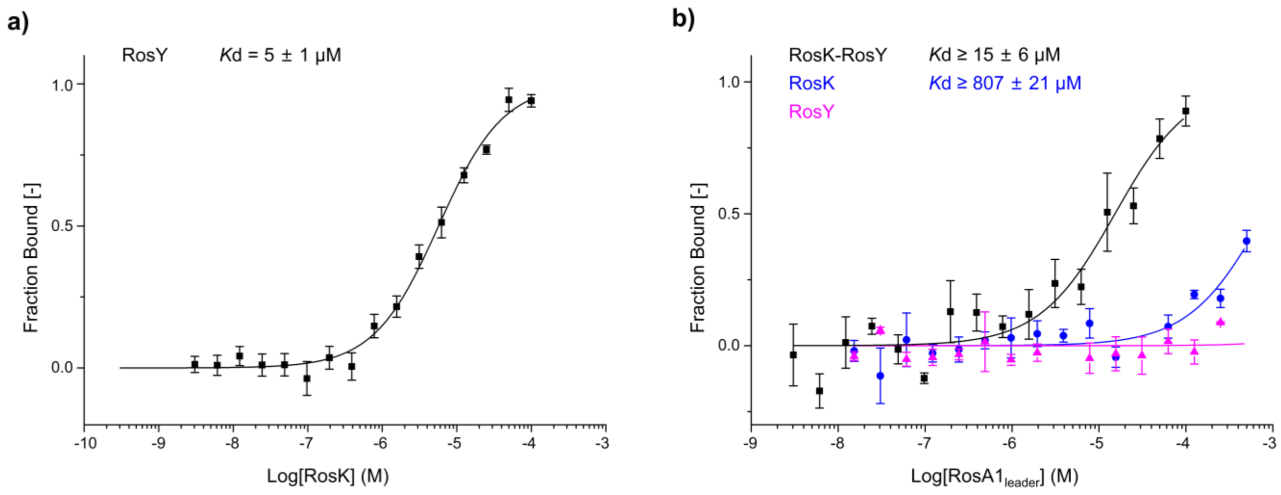

Figure S23. (a). Binding affinity between RosK and His_6-RosY measured by MST. (b). Binding affinities between RosA1$_{leader}$ and His_6-RosK-RosY, His_6-RosK, His_6-RosY measured by MST. Data represent the mean ± s.d. from three replicates.

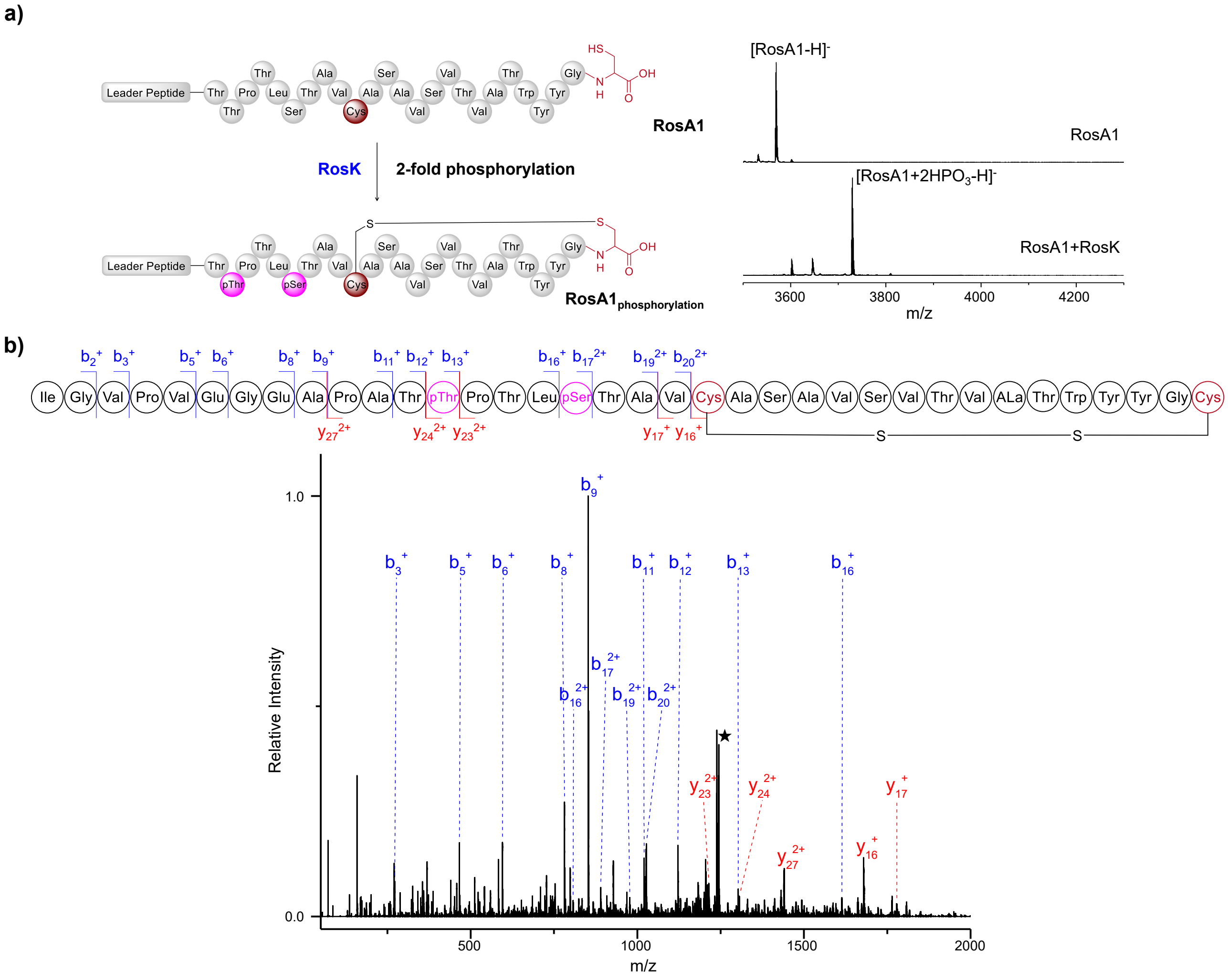

Ions	calc.	obs.	Er. (ppm)	Ions	calc.	obs.	Er. (ppm)
b_2^+	171.1129	171.1123	-.3.5	y_{16}^+	1678.7240	1678.7247	0.4
b_3^+	270.1813	270.1805	-3.0	y_{17}^+	1777.7924	1777.7931	0.4
b_5^+	466.3024	466.3015	-1.9	y_{23}^{2+}	1214.5337	1214.5334	-0.2
b_6^+	595.3450	595.3442	-1.3	y_{24}^{2+}	1305.0407	1305.0450	3.3
b_8^+	781.4091	781.4093	0.3	y_{27}^{2+}	1439.6095	1439.6116	1.5
b_9^+	852.4462	852.4430	-3.8				
b_{11}^+	1020.5361	1020.5360	-0.1				
b_{12}^+	1121.5838	1121.5850	1.1				
b_{13}^+	1302.5978	1302.5948	-2.3				
b_{16}^+	1613.7823	1613.7831	0.5				
b_{16}^{2+}	807.3948	807.3925	-2.8				
b_{17}^{2+}	890.8940	890.8959	2.1				
b_{19}^{2+}	976.9364	976.9378	1.4				
b_{20}^{2+}	1026.4706	1026.4756	4.9				

Figure S24. Phosphorylation of RosA1 by RosK. (a). RosK performs 2-fold phosphorylation of residues Thr2 and Ser6 of RosA1, as determined by MALDI-TOF-MS analysis. $[RosA1\text{-}H]^-$: $M_{calc.}$ = 3567.70 Da, $M_{obs.}$ = 3567.65 Da; $[RosA1+2HPO_3\text{-}H]^-$: $M_{calc.}$ = 3727.64 Da, $M_{obs.}$ = 3727.95 Da. The disulfide bond was formed by the air oxidation of Cys residues during sample preparation. (b). MS/MS analysis of RosA1$_{phosphorylation}$. The *b* and *y* ions are listed in table and marked in the spectrum. The asterisk indicates $[M+3H]^{3+}$.

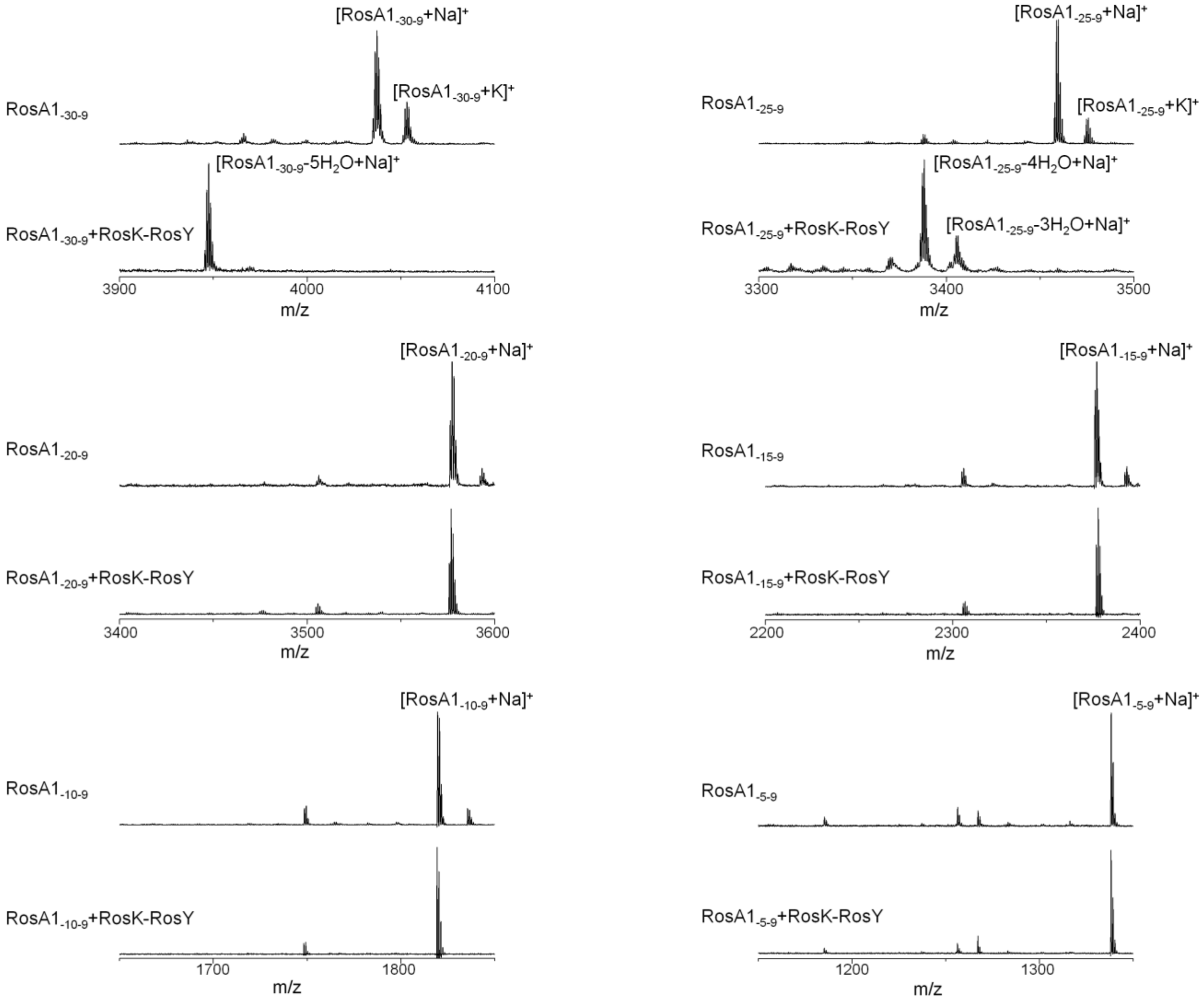

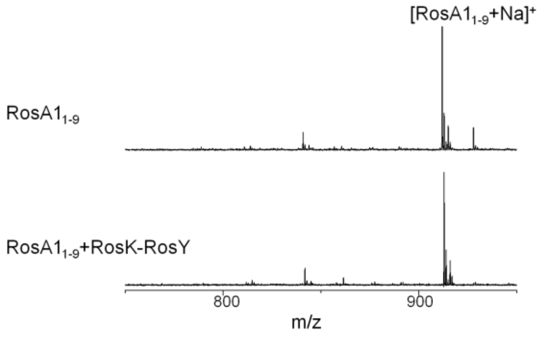

Substrate	Sequence	Dehydration
RosA1$_{-30-9}$	MDTTEQLIAGYTAYTDAEEIGVPVEGEAPATTPTLSTAV	-5 H_2O
RosA1$_{-25-9}$	QLIAGYTAYTDAEEIGVPVEGEAPATTPTLSTAV	-4 H_2O
RosA1$_{-20-9}$	YTAYTDAEEIGVPVEGEAPATTPTLSTAV	×
RosA1$_{-15-9}$	DAEEIGVPVEGEAPATTPTLSTAV	×
RosA1$_{-10-9}$	GVPVEGEAPATTPTLSTAV	×
RosA1$_{-5-9}$	GEAPATTPTLSTAV	×
RosA1$_{1-9}$	TTPTLSTAV	×

Figure S25. MALDI-TOF-MS analysis of the *in vitro* enzymatic modification of RosA1 variants by the RosK-RosY complex.

[RosA1$_{-30-9}$+Na]$^+$: $M_{calc.}$ = 4034.89 Da, $M_{obs.}$ = 4035.22 Da; [RosA1$_{-30-9}$-5H_2O+Na]$^+$: $M_{calc.}$ = 3944.84 Da, $M_{obs.}$ = 3945.37 Da; [RosA1$_{-25-9}$+Na]$^+$: $M_{calc.}$ = 3457.69 Da, $M_{obs.}$ = 3457.71 Da; [RosA1$_{-25-9}$-4H_2O+Na]$^+$: $M_{calc.}$ = 3385.65 Da, $M_{obs.}$ = 3386.07 Da; [RosA1$_{-25-9}$-3H_2O+Na]$^+$: $M_{calc.}$ = 3403.66 Da, $M_{obs.}$ = 3404.07 Da; [RosA1$_{-20-9}$+Na]$^+$: $M_{calc.}$ = 2975.40 Da, $M_{obs.}$ = 2975.81 Da; [RosA1$_{-15-9}$+Na]$^+$: $M_{calc.}$ = 2376.14 Da, $M_{obs.}$ = 2376.61 Da; [RosA1$_{-10-9}$+Na]$^+$: $M_{calc.}$ = 1818.91 Da, $M_{obs.}$ = 1819.32 Da; [RosA1$_{-5-9}$+Na]$^+$: $M_{calc.}$ = 1337.66 Da, $M_{obs.}$ = 1338.02 Da; [RosA1$_{1-9}$+Na]$^+$: $M_{calc.}$ = 912.46 Da, $M_{obs.}$ = 912.88 Da;

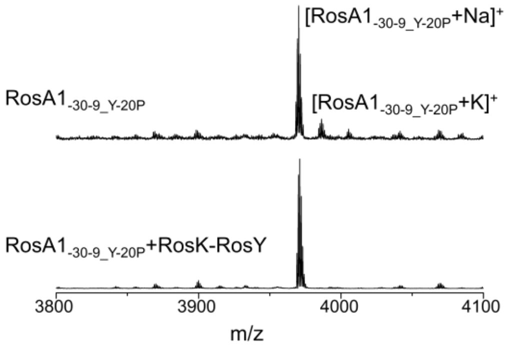

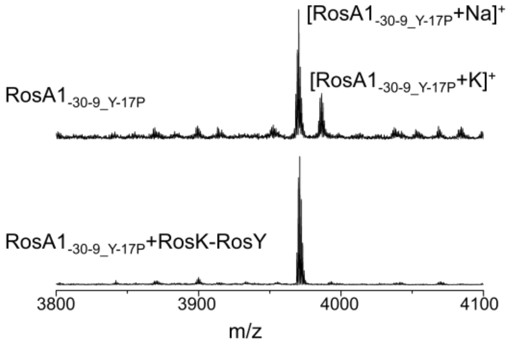

Figure S26. Helix-breaking mutation in the leader peptide abolished the modification of RosA1$_{-30-9}$ by RosK-RosY, as determined by MALDI-TOF-MS analysis.
[RosA1$_{-30-9_Y-20P}$+Na]$^+$: $M_{calc.}$=3968.88 Da, $M_{obs.}$=3969.31 Da; [RosA1$_{-30-9_Y-20P}$+K]$^+$: $M_{calc.}$=3984.86 Da, $M_{obs.}$=3985.26 Da;
[RosA1$_{-30-9_Y-17P}$+Na]$^+$: $M_{calc.}$=3968.88 Da, $M_{obs.}$=4969.23 Da; [RosA1$_{-30-9_Y-17P}$+K]$^+$: $M_{calc.}$=3984.86 Da, $M_{obs.}$=3985.24 Da.

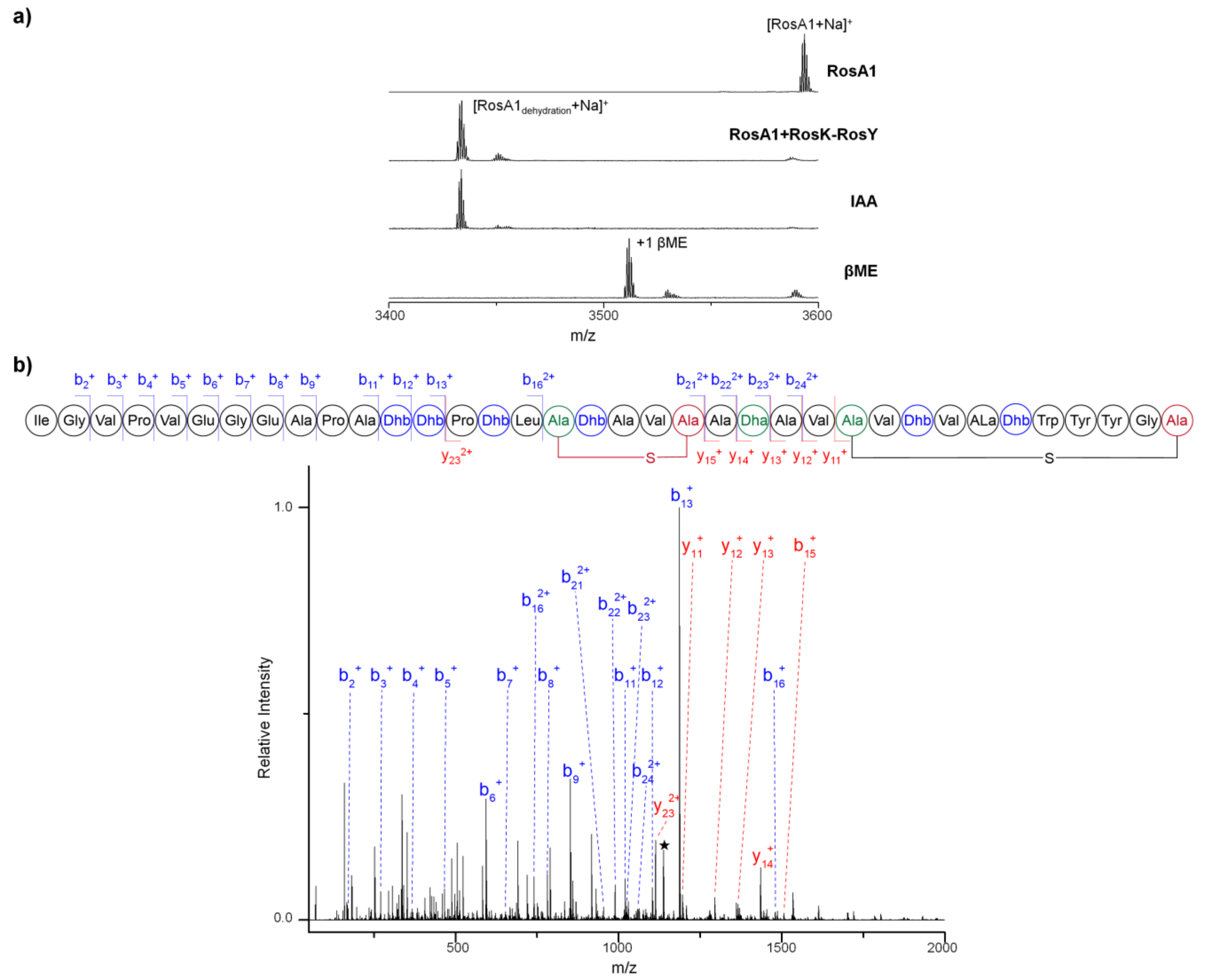

Ions	calc.	obs.	Er. (ppm)	Ions	calc.	obs.	Er. (ppm)
b_2^+	171.1129	171.1127	-1.2	y_{11}^+	1195.5242	1195.5222	-1.7
b_3^+	270.1813	270.1814	0.4	y_{12}^+	1294.5926	1294.5903	-1.8
b_4^+	367.2340	367.2343	0.8	y_{13}^+	1365.6297	1365.6273	-1.8
b_5^+	466.3024	466.3019	-1.1	y_{14}^+	1434.6511	1434.6461	-3.5
b_6^+	595.3450	595.3441	-1.5	y_{15}^+	1505.6883	1505.6864	-1.3
b_7^+	652.3665	652.3654	-1.7	y_{23}^{2+}	1112.5214	1112.5224	0.9
b_8^+	781.4091	781.4087	-0.5				
b_9^+	852.4462	852.4448	-1.6				
b_{11}^+	1020.5361	1020.5344	-1.7				
b_{12}^+	1103.5732	1103.5720	-1.1				
b_{13}^+	1186.6103	1186.6075	-2.4				
b_{16}^+	1479.7843	1479.7851	0.5				
b_{16}^{2+}	740.3958	740.3964	0.8				
b_{21}^{2+}	952.9824	952.9843	2.0				
b_{22}^{2+}	988.5010	988.5010	0				
b_{23}^{2+}	1023.0117	1023.0107	-1.0				
b_{24}^{2+}	1058.5303	1058.5335	3.0				

Figure S27. (a). MALDI-TOF-MS analysis of the (RosK-RosY)-modified RosA1 after GluC digestion and derivatization. (b). MS/MS analysis of $RosA1_{dehydration}$ after GluC digestion. The *b* and *y* ions are listed in table and marked in the spectrum. The asterisk indicates $[M+3H]^{3+}$.

$[RosA1+Na]^+$: $M_{calc.}$ = 3593.72 Da, $M_{obs.}$ = 3593.58 Da;

$[RosA1_{dehydration}+Na]^+$: $M_{calc.}$ = 3431.62 Da, $M_{obs.}$ = 3431.57 Da;

$[RosA1_{dehydration}+\beta ME+Na]^+$: $M_{calc.}$=3509.63 Da, $M_{obs.}$=3509.86 Da.

c)

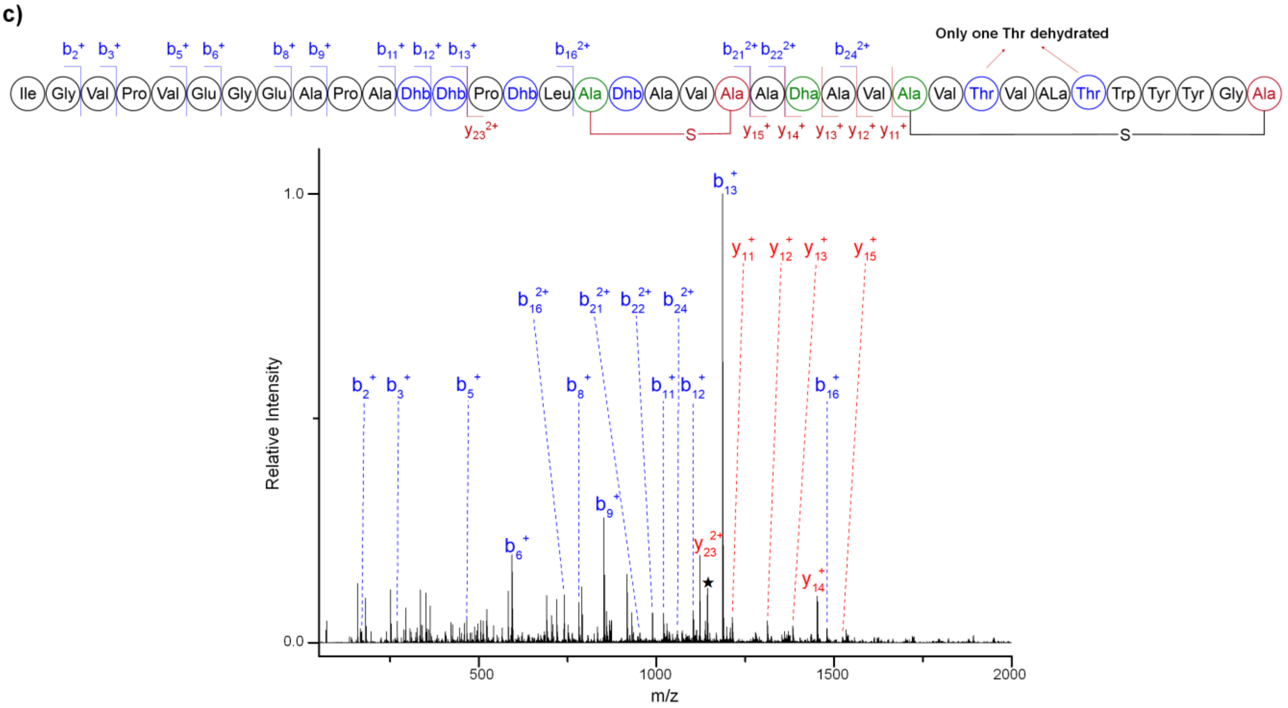

Ions	calc.	obs.	Er. (ppm)	Ions	calc.	obs.	Er. (ppm)
b_2^+	171.1129	171.1124	-2.9	y_{11}^+	1213.5347	1213.5349	0.2
b_3^+	270.1813	270.1811	-0.7	y_{12}^+	1312.6031	1312.6025	-0.5
b_5^+	466.3024	466.3019	-1.1	y_{13}^+	1383.6402	1383.6415	0.9
b_6^+	595.3450	595.3440	-1.7	y_{14}^+	1452.6617	1452.6611	-0.4
b_8^+	781.4091	781.4074	-2.2	y_{15}^+	1523.6988	1523.6993	0.3
b_9^+	852.4462	852.4461	-0.1	y_{23}^{2+}	1121.5267	1121.5294	2.4
b_{11}^+	1020.5361	1020.5322	-3.8				
b_{12}^+	1103.5732	1103.5717	-1.4				
b_{13}^+	1186.6103	1186.6070	-2.8				
b_{16}^+	1479.7843	1479.7848	0.3				
b_{16}^{2+}	740.3958	740.3974	2.2				
b_{21}^{2+}	952.9824	952.9860	3.8				
b_{22}^{2+}	988.5010	988.5020	1.0				
b_{24}^{2+}	1058.5303	1058.5324	2.0				

Figure S27. (c). MS/MS analysis of RosA1-8 H_2O after GluC digestion. The *b* and *y* ions are listed in table and marked in the spectrum. The asterisk indicates $[M+3H]^{3+}$.

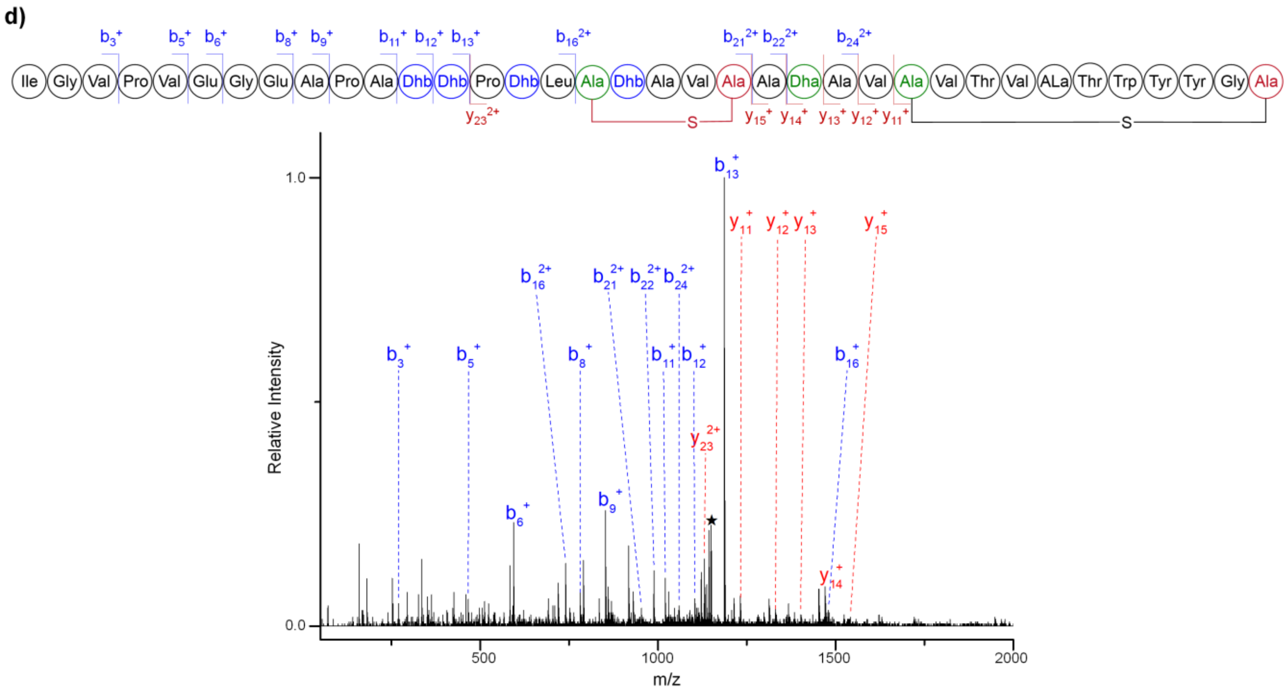

Ions	calc.	obs.	Er. (ppm)	Ions	calc.	obs.	Er. (ppm)
b_3^+	270.1813	270.1818	1.9	y_{11}^+	1231.5453	1231.5453	0
b_5^+	466.3024	466.3010	-3.0	y_{12}^+	1330.6137	1330.6142	0.4
b_6^+	595.3450	595.3430	-3.4	y_{13}^+	1401.6508	1401.6472	-2.6
b_8^+	781.4091	781.4081	-1.3	y_{14}^+	1470.6723	1470.6674	-3.3
b_9^+	852.4462	852.4438	-2.8	y_{15}^+	1541.7094	1541.7034	-3.9
b_{11}^+	1020.5361	1020.5334	-2.6	y_{23}^{2+}	1130.5320	1130.5338	1.6
b_{12}^+	1103.5732	1103.5759	2.4				
b_{13}^+	1186.6103	1186.6089	-1.2				
b_{16}^+	1479.7843	1479.7848	0.3				
b_{16}^{2+}	740.3958	740.3965	0.9				
b_{21}^{2+}	952.9824	952.9820	-0.4				
b_{22}^{2+}	988.5010	988.4975	-3.5				
b_{24}^{2+}	1058.5303	1058.5336	3.1				

Figure S27. (d). MS/MS analysis of RosA1-7 H_2O after GluC digestion. The *b* and *y* ions are listed in table and marked in the spectrum. The asterisk indicates $[M+3H]^{3+}$.

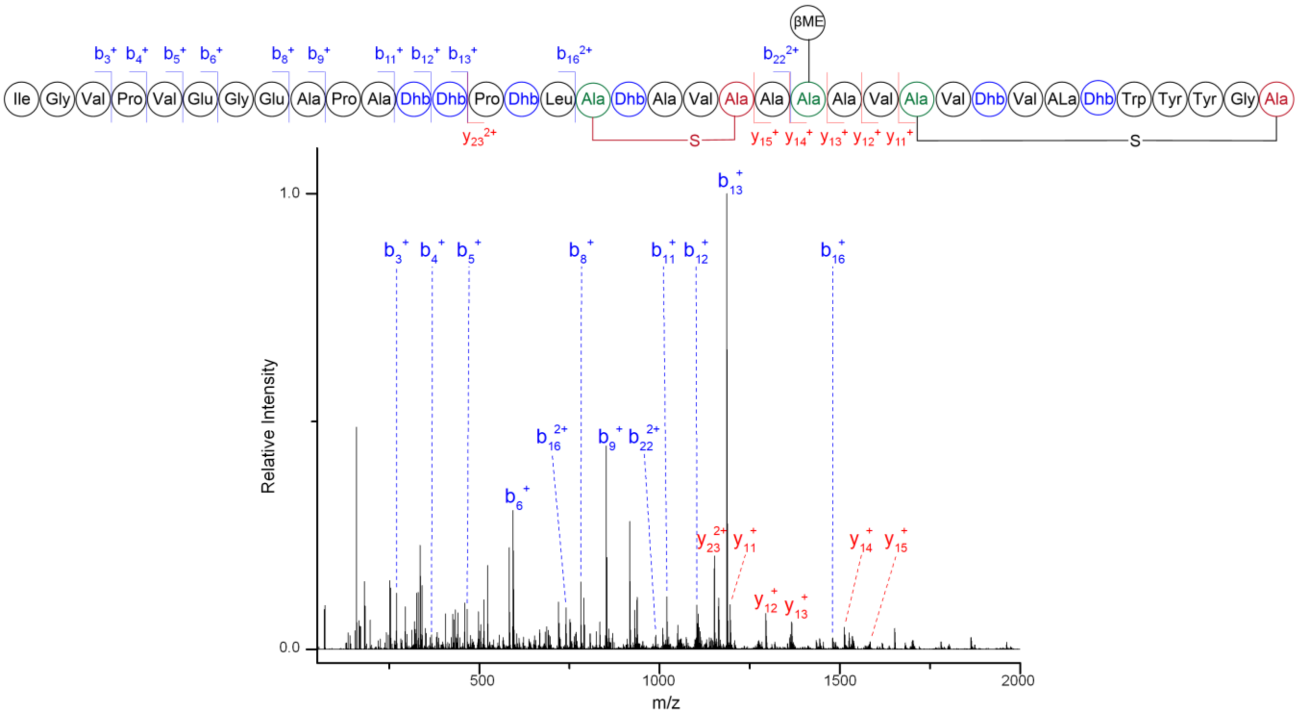

Ions	calc.	obs.	Er. (ppm)	Ions	calc.	obs.	Er. (ppm)
b_3^+	270.1813	270.1814	0.4	y_{11}^+	1195.5242	1195.5193	-4.1
b_4^+	367.2340	367.2325	-4.1	y_{12}^+	1294.5926	1294.5908	-1.4
b_5^+	466.3024	466.3015	-1.9	y_{13}^+	1365.6297	1365.6273	-1.8
b_6^+	595.3450	595.3431	-3.2	y_{14}^+	1512.6651	1512.6679	1.9
b_8^+	781.4091	781.4079	-1.5	y_{15}^+	1583.7022	1583.6953	-4.4
b_9^+	852.4462	852.4444	-2.1	y_{23}^{2+}	1151.5284	1151.5299	1.3
b_{11}^+	1020.5361	1020.5343	-1.8				
b_{12}^+	1103.5732	1103.5713	-1.7				
b_{13}^+	1186.6103	1186.6089	-1.2				
b_{16}^+	1479.7843	1479.7801	-2.8				
b_{16}^{2+}	740.3958	740.3936	-3.0				
b_{22}^{2+}	988.5010	988.4998	-1.2				

Figure S28. LC-MS/MS analysis of RosA1$_{\text{dehydration}}$-βME adduct after GluC digestion. The *b* and *y* ions are listed in table and marked in the spectrum.

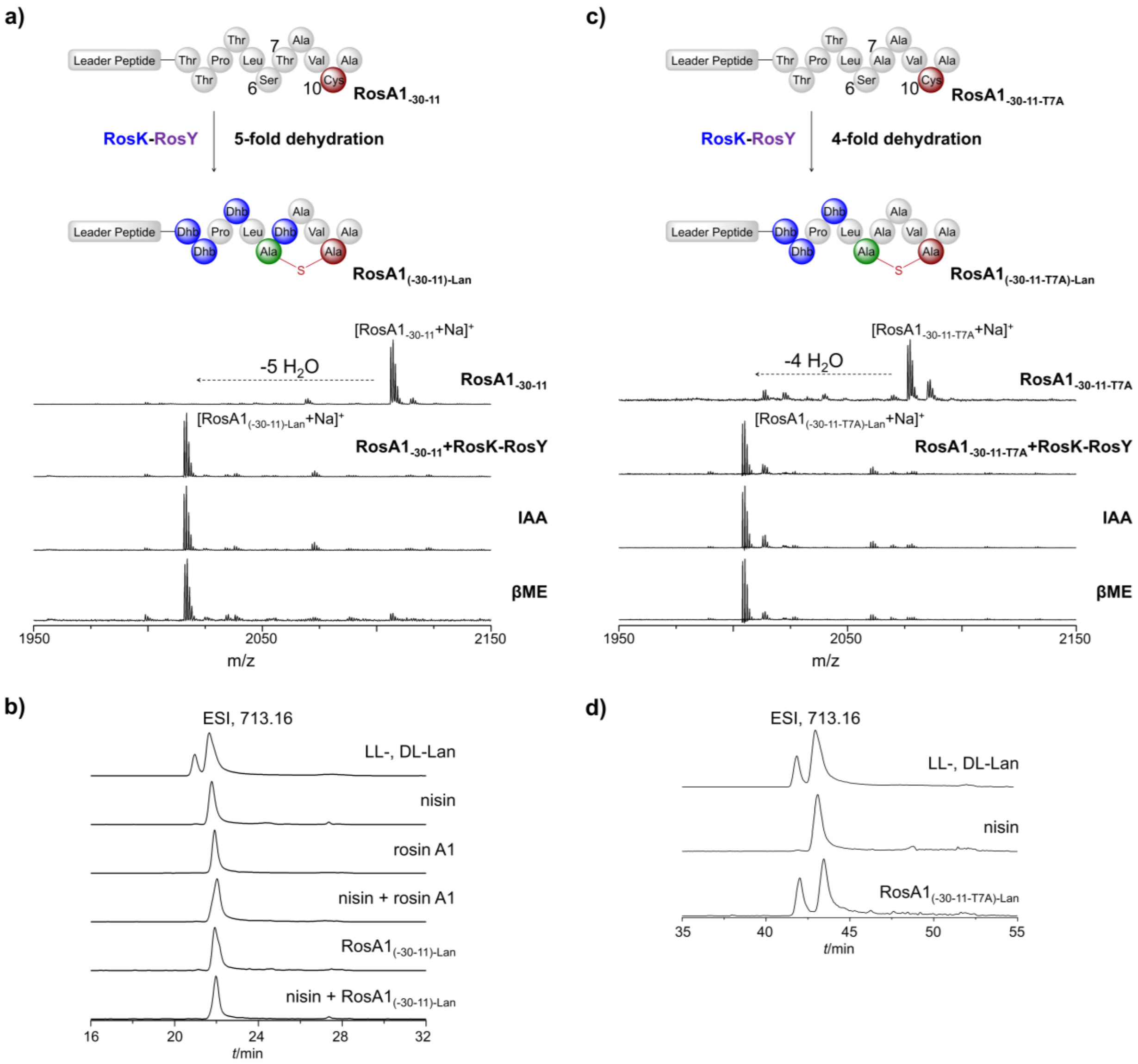

Figure S29. (a). MALDI-TOF-MS analysis of the *in vitro* enzymatic modification of RosA1$_{-30-11}$ by RosK-RosY after GluC digestion and derivatization. [RosA1$_{-30-11}$+Na]$^+$: $M_{calc.}$ = 2106.04 Da, $M_{obs.}$ = 2106.18 Da; [RosA1$_{(-30-11)\text{-}Lan}$+Na]$^+$: $M_{calc.}$ = 2015.99 Da, $M_{obs.}$ = 2015.85 Da; (b). Marfey's analysis of the RosA1$_{(-30-11)\text{-}Lan}$, using rosinA1 as a control. (c). MALDI-TOF-MS analysis of the *in vitro* enzymatic modification of RosA1$_{-30-11\text{-}T7A}$ by RosK-RosY after GluC digestion. [RosA1$_{-30-11\text{-}T7A}$+Na]$^+$: $M_{calc.}$ = 2076.03 Da, $M_{obs.}$ = 2076.35 Da; [RosA1$_{(-30-11\text{-}T7A)\text{-}Lan}$+Na]$^+$: $M_{calc.}$ = 2003.99 Da, $M_{obs.}$ = 2004.10 Da; (d). Marfey's analysis of the RosA1$_{(-30-11\text{-}T7A)\text{-}Lan}$.

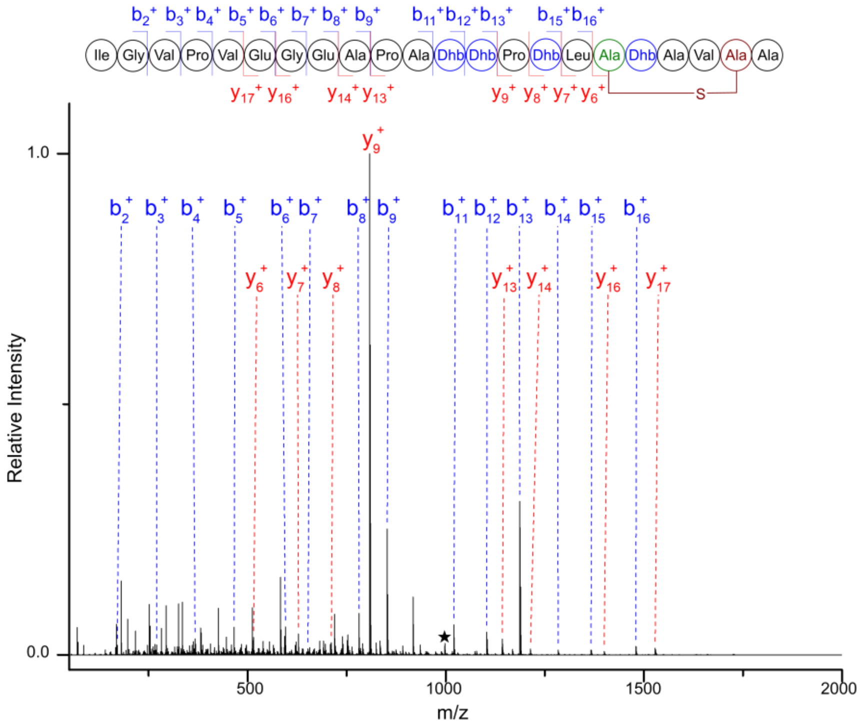

Ions	calc.	obs.	Er. (ppm)	Ions	calc.	obs.	Er. (ppm)
b_2^+	171.1129	171.1130	0.6	y_6^+	515.2283	515.2285	0.4
b_3^+	270.1813	270.1809	-1.5	y_7^+	628.3124	628.3127	0.5
b_4^+	367.2340	367.2335	-1.4	y_8^+	711.3495	711.3506	1.5
b_5^+	466.3024	466.3022	-0.4	y_9^+	808.4022	808.4003	-2.4
b_6^+	595.3450	595.3443	-1.2	y_{13}^+	1142.5664	1142.5673	0.8
b_7^+	652.3665	652.3663	-0.3	y_{14}^+	1213.6035	1213.6042	0.6
b_8^+	781.4091	781.4086	-0.6	y_{16}^+	1399.6675	1399.6675	0
b_9^+	852.4462	852.4451	-1.3	y_{17}^+	1528.7101	1528.7095	-0.4
b_{11}^+	1020.5361	1020.5354	-0.7				
b_{12}^+	1103.5732	1103.5731	-0.1				
b_{13}^+	1186.6103	1186.6088	-1.3				
b_{14}^+	1283.6631	1283.6639	0.6				
b_{15}^+	1366.7002	1366.7003	0.1				
b_{16}^+	1479.7843	1479.7860	1.1				

Figure S30. LC-MS/MS analysis of RosA1$_{(-30-11)\text{-Lan}}$ after GluC digestion. The *b* and *y* ions are listed in table and marked in the spectrum. The asterisk indicates $[M+2H]^{2+}$.

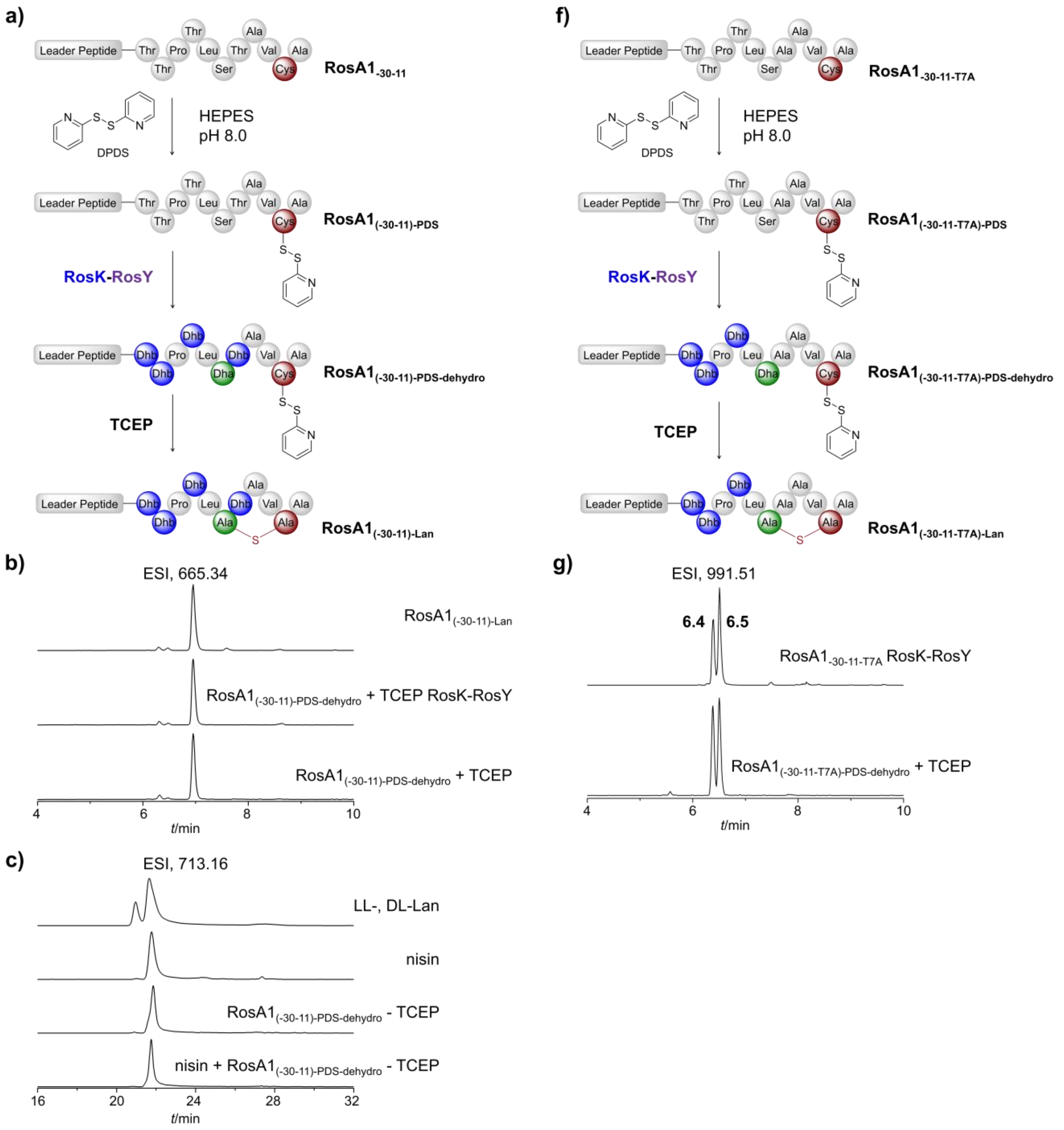

Figure S31. (a). PDS protection strategy of cysteine in RosA1$_{-30-11}$. (b). EIC profiles of RosA1$_{(-30-11)\text{-PDS-dehydro}}$ treated with TCEP and RosK-RosY together, and with TCEP alone, using RosA1$_{(-30-11)\text{-Lan}}$ as a control. (c). Marfey's analysis of the products obtained after treating RosA1$_{(-30-11)\text{-PDS-dehydro}}$ with TCEP alone. (f). PDS protection strategy of cysteine in RosA1$_{-30-11\text{-T7A}}$. (g). EIC profiles of the *in vitro* enzymatic modification of RosA1$_{-30-11\text{-T7A}}$ by RosK-RosY and RosA1$_{(-30-11\text{-T7A})\text{-PDS-dehydro}}$ treated with TCEP after GluC digestion.

d)

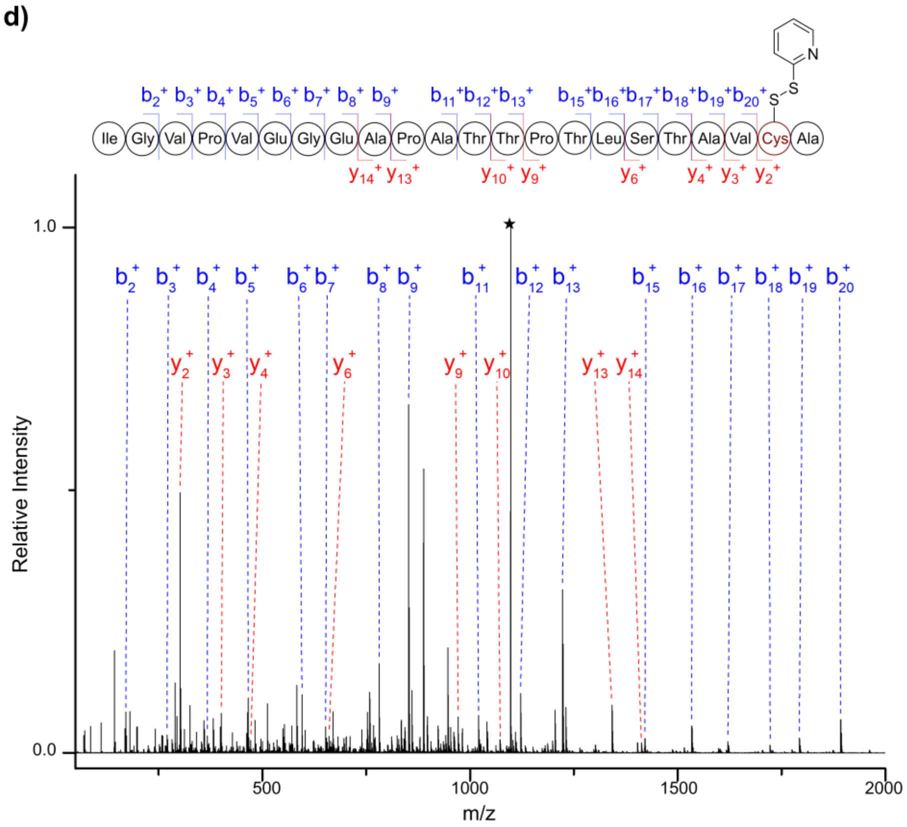

Ions	calc.	obs.	Er. (ppm)	Ions	calc.	obs.	Er. (ppm)
b_2^+	171.1129	171.1125	-2.3	y_2^+	302.0628	302.0621	-2.3
b_3^+	270.1813	270.1807	-2.2	y_3^+	401.1312	401.1300	-3.0
b_4^+	367.2340	367.2330	-2.7	y_4^+	472.1683	472.1672	-2.3
b_5^+	466.3024	466.3010	-3.0	y_6^+	660.2480	660.2475	-0.8
b_6^+	595.3450	595.3438	-2.0	y_9^+	971.4325	971.4324	-0.1
b_7^+	652.3665	652.3650	-2.3	y_{10}^+	1072.4802	1072.4792	-0.9
b_8^+	781.4091	781.4081	-1.3	y_{13}^+	1341.6178	1341.6170	-0.6
b_9^+	852.4462	852.4445	-2.0	y_{14}^+	1412.6549	1412.6525	-1.7
b_{11}^+	1020.5361	1020.5357	-0.4				
b_{12}^+	1121.5838	1121.5834	-0.4				
b_{13}^+	1222.6314	1222.6307	-0.6				
b_{15}^+	1420.7319	1420.7306	-0.9				
b_{16}^+	1533.8159	1533.8148	-0.7				
b_{17}^+	1620.8480	1620.8462	-1.1				
b_{18}^+	1721.8957	1721.8941	-0.9				
b_{19}^+	1792.9328	1792.9307	-1.2				
b_{20}^+	1892.0012	1892.0013	0.1				

Figure S31. (d). LC-MS/MS analysis of RosA1$_{(-30-11)\text{-PDS}}$ after GluC digestion. The *b* and *y* ions are listed in table and marked in the spectrum. The asterisk indicates $[M+2H]^{2+}$.

e)

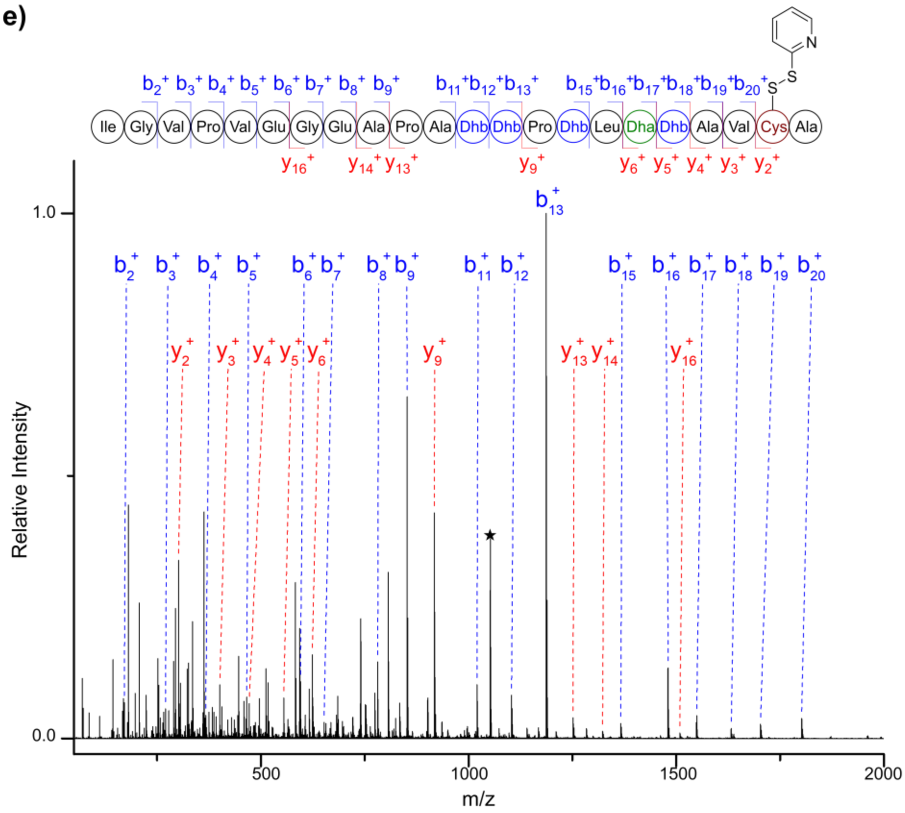

Ions	calc.	obs.	Er. (ppm)	Ions	calc.	obs.	Er. (ppm)
b_2^+	171.1129	171.1125	-2.3	y_2^+	302.0628	302.0619	-3.0
b_3^+	270.1813	270.1810	-1.1	y_3^+	401.1312	401.1303	-2.2
b_4^+	367.2340	367.2325	-4.1	y_4^+	472.1683	472.1670	-2.8
b_5^+	466.3024	466.3014	-2.1	y_5^+	555.2055	555.2046	-1.6
b_6^+	595.3450	595.3440	-1.7	y_6^+	624.2269	624.2262	-1.1
b_7^+	652.3665	652.3669	0.6	y_9^+	917.4009	917.4050	4.5
b_8^+	781.4091	781.4095	0.5	y_{13}^+	1251.5650	1251.5664	1.1
b_9^+	852.4462	852.4456	-0.7	y_{14}^+	1322.6021	1322.6057	2.7
b_{11}^+	1020.5361	1020.5359	-0.2	y_{16}^+	1508.6662	1508.6675	0.9
b_{12}^+	1103.5732	1103.5739	0.6				
b_{13}^+	1186.6103	1186.6102	-0.1				
b_{15}^+	1366.7002	1366.7012	0.7				
b_{16}^+	1479.7843	1479.7859	1.1				
b_{17}^+	1548.8057	1548.8102	2.9				
b_{18}^+	1631.8429	1631.8470	2.5				
b_{19}^+	1702.8800	1702.8786	-0.8				
b_{20}^+	1801.9484	1801.9486	0.1				

Figure S31. (e). LC-MS/MS analysis of RosA1$_{(-30-11)\text{-PDS-dehydro}}$ after GluC digestion. The *b* and *y* ions are listed in table and marked in the spectrum. The asterisk indicates [M+2H]$^{2+}$.

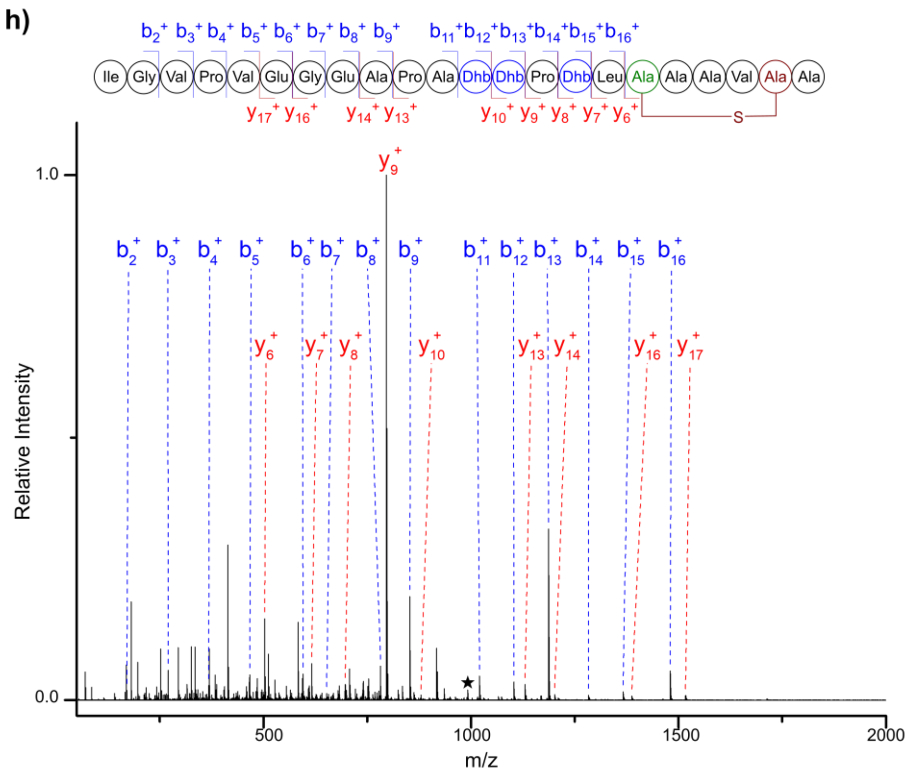

Ions	calc.	obs.	Er. (ppm)	Ions	calc.	obs.	Er. (ppm)
b_2^+	171.1129	171.1129	0	y_6^+	503.2283	503.2279	-0.8
b_3^+	270.1813	270.1823	3.7	y_7^+	616.3124	616.3124	0
b_4^+	367.2340	367.2342	0.5	y_8^+	699.3495	699.3475	-2.9
b_5^+	466.3024	466.3021	-0.6	y_9^+	796.4022	796.4013	-1.1
b_6^+	595.3450	595.3454	0.7	y_{10}^+	879.4394	879.4396	0.2
b_7^+	652.3665	652.3663	-0.3	y_{13}^+	1130.5664	1130.5659	-0.4
b_8^+	781.4091	781.4096	0.6	y_{14}^+	1201.6035	1201.6039	0.3
b_9^+	852.4462	852.4459	-0.4	y_{16}^+	1387.6675	1387.6626	-3.5
b_{11}^+	1020.5361	1020.5359	-0.2	y_{17}^+	1516.7101	1516.7074	-1.8
b_{12}^+	1103.5732	1103.5725	-0.6				
b_{13}^+	1186.6103	1186.6092	-0.9				
b_{14}^+	1283.6631	1283.6622	-0.7				
b_{15}^+	1366.7002	1366.6996	-0.4				
b_{16}^+	1479.7843	1479.7816	-1.8				

Figure S31. (h). LC-MS/MS analysis of product with a retention time of **6.4** minutes. The *b* and *y* ions are listed in table and marked in the spectrum. The asterisk indicates $[M+2H]^{2+}$.

i)

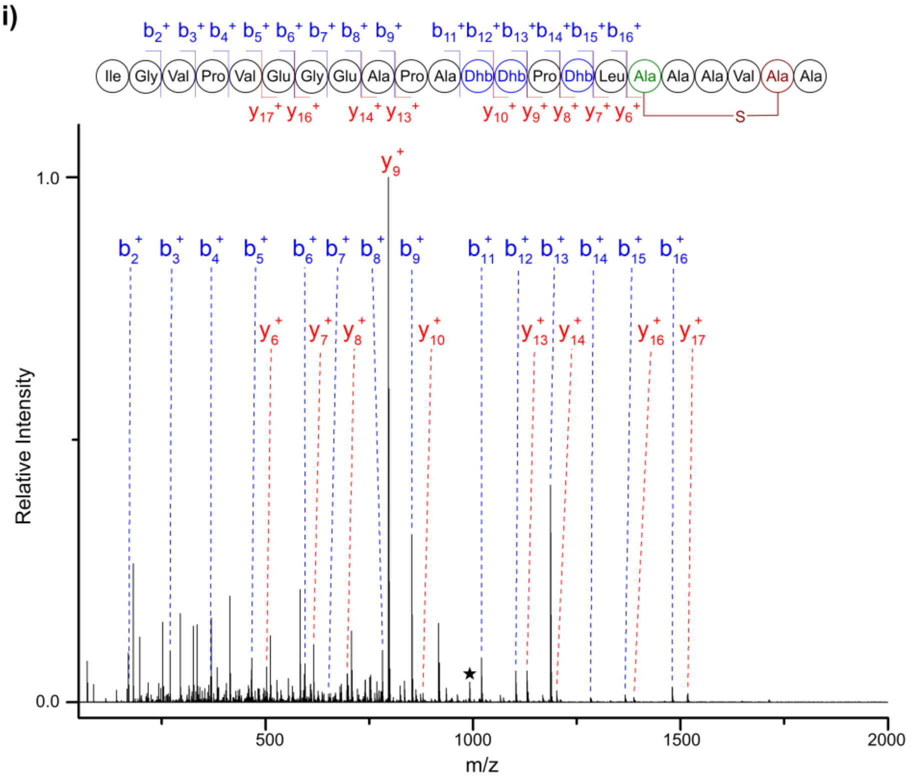

Ions	calc.	obs.	Er. (ppm)	Ions	calc.	obs.	Er. (ppm)
b_2^+	171.1129	171.1131	1.2	y_6^+	503.2283	503.2286	0.6
b_3^+	270.1813	270.1822	3.3	y_7^+	616.3124	616.3130	1.0
b_4^+	367.2340	367.2338	-0.5	y_8^+	699.3495	699.3483	-1.7
b_5^+	466.3024	466.3026	0.4	y_9^+	796.4022	796.4021	-0.1
b_6^+	595.3450	595.3453	0.5	y_{10}^+	879.4394	879.4408	1.6
b_7^+	652.3665	652.3659	-0.9	y_{13}^+	1130.5664	1130.5686	1.9
b_8^+	781.4091	781.4096	0.6	y_{14}^+	1201.6035	1201.6047	1.0
b_9^+	852.4462	852.4473	1.3	y_{16}^+	1387.6675	1387.6680	0.4
b_{11}^+	1020.5361	1020.5391	2.9	y_{17}^+	1516.7101	1516.7101	0
b_{12}^+	1103.5732	1103.5746	1.3				
b_{13}^+	1186.6103	1186.6106	0.3				
b_{14}^+	1283.6631	1283.6632	0.1				
b_{15}^+	1366.7002	1366.7000	-0.1				
b_{16}^+	1479.7843	1479.7884	2.8				

Figure S31. (i). LC-MS/MS analysis of the product with a retention time of **6.5** minutes. The *b* and *y* ions are listed in table and marked in the spectrum. The asterisk indicates $[M+2H]^{2+}$.

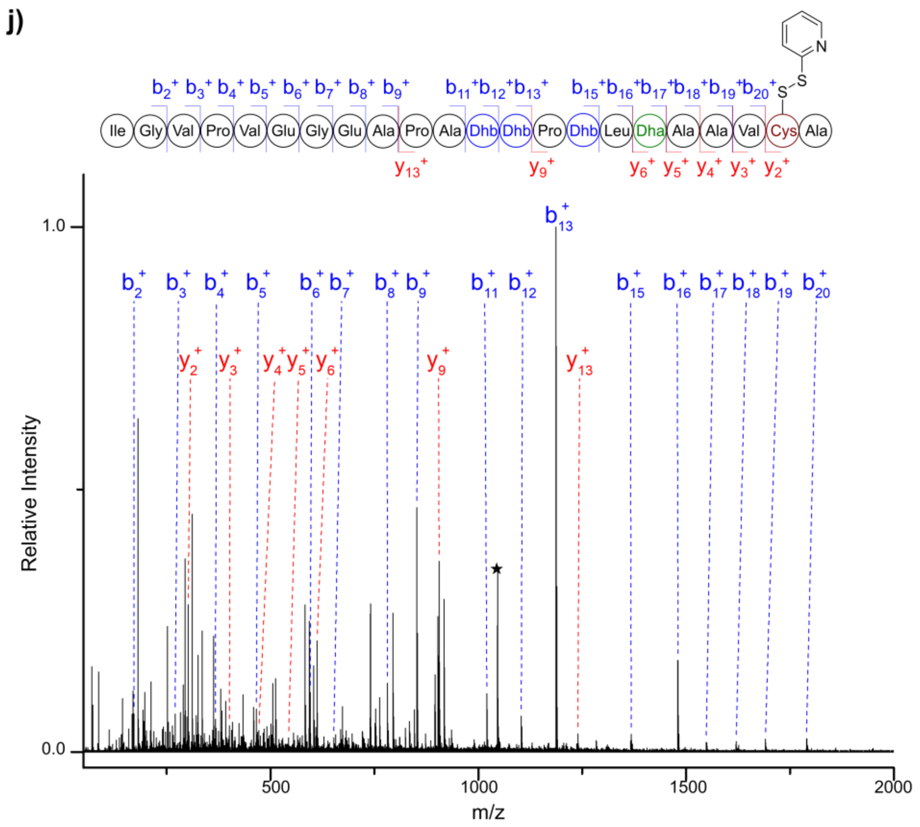

Ions	calc.	obs.	Er. (ppm)	Ions	calc.	obs.	Er. (ppm)
b_2^+	171.1129	171.1130	0.6	y_2^+	302.0628	302.0627	-0.3
b_3^+	270.1813	270.1815	0.7	y_3^+	401.1312	401.1299	-3.2
b_4^+	367.2340	367.2323	-4.6	y_4^+	472.1683	472.1662	-4.4
b_5^+	466.3024	466.3044	4.3	y_5^+	543.2055	543.2064	1.7
b_6^+	595.3450	595.3476	4.4	y_6^+	612.2269	612.2281	2.0
b_7^+	652.3665	652.3677	1.8	y_9^+	905.4009	905.4052	4.7
b_8^+	781.4091	781.4110	2.4	y_{13}^+	1239.5650	1239.5709	4.8
b_9^+	852.4462	852.4477	1.8	y_{14}^+	1310.6021	1310.6041	1.5
b_{11}^+	1020.5361	1020.5409	4.7				
b_{12}^+	1103.5732	1103.5778	4.2				
b_{13}^+	1186.6103	1186.6147	3.7				
b_{15}^+	1366.7002	1366.7066	4.7				
b_{16}^+	1479.7843	1479.7913	4.7				
b_{17}^+	1548.8057	1548.8007	-3.2				
b_{18}^+	1619.8428	1619.8499	4.4				
b_{19}^+	1690.8800	1690.8816	0.9				
b_{20}^+	1789.9484	1789.9478	-0.3				

Figure S31. (j). LC-MS/MS analysis of RosA1$_{(-30-11-T7A)-PDS-dehydro}$ after GluC digestion. The *b* and *y* ions are listed in table and marked in the spectrum. The asterisk indicates $[M+2H]^{2+}$.

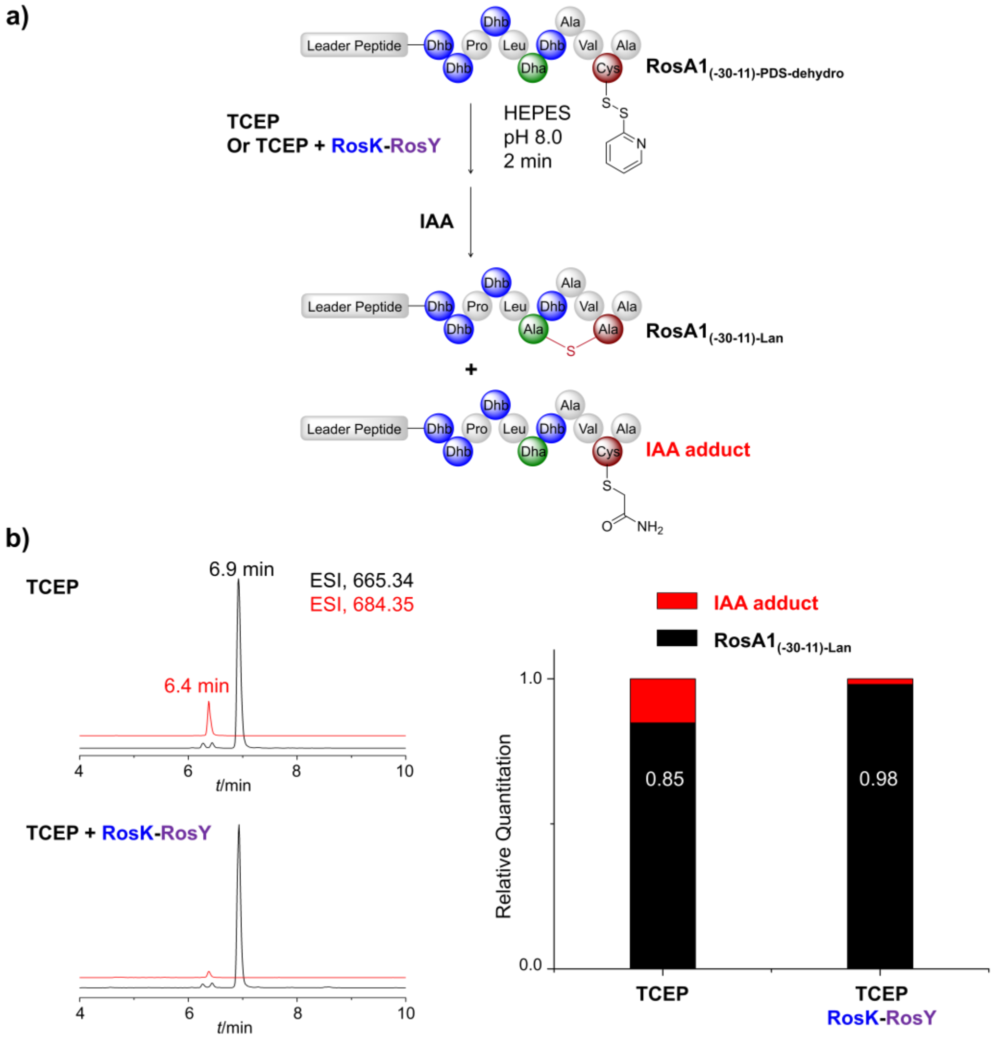

Figure S32. (a). An IAA-based method to analyze the efficiency of Lan macrocyclization in RosA1$_{(-30-11)\text{-PDS-dehydro}}$ in the presence and absence of the RosK-RosY complex. Condition: 100 µM peptide substrate, 1 mM TCEP, 50 mM HEPES, pH 8.0, 5 mM ATP and 1 mM $MgCl_2$ for 2 minutes with or without RosK-RosY (100 µM), followed by treatment with IAA. The protocol is adopted from reference.[19] (b). EICs and relative quantification chart of the two products, RosA1$_{(-30-11)\text{-Lan}}$ and IAA adduct, in the presence or absence of RosK-RosY.

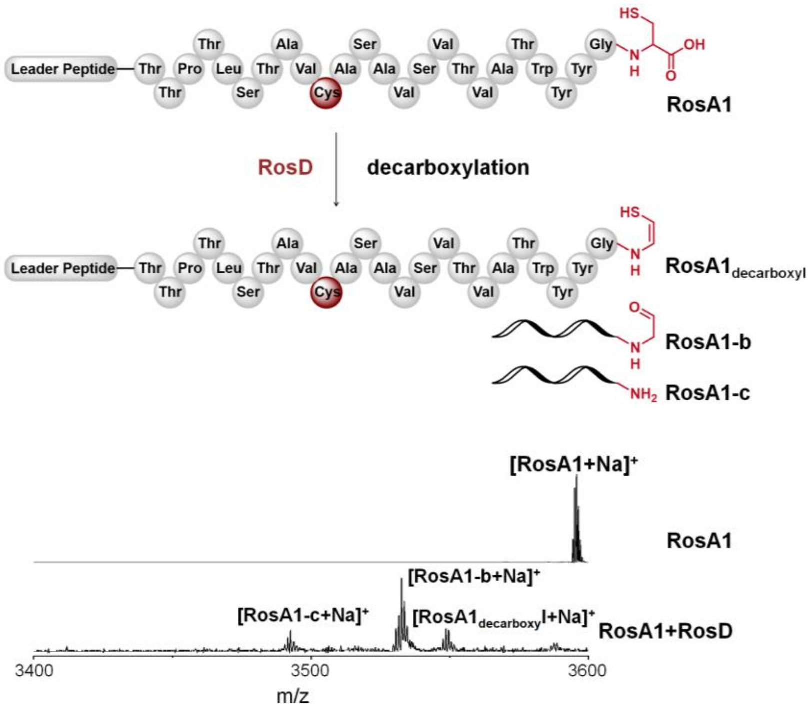

Figure S33. MALDI-TOF-MS analysis of decarboxylation of RosA1 by RosD *in vitro.*
$[RosA1+Na]^+$: M_{calc}. = 3593.72 Da, M_{obs}. = 3593.58 Da;
$[RosA1_{decarboxyl}+Na]^+$: M_{calc}. = 3547.71 Da, M_{obs}. = 3547.59 Da;
$[RosA1\text{-}b+Na]^+$: M_{calc}.=3531.73 Da, M_{obs}.=3531.62 Da;
$[RosA1\text{-}c+Na]^+$: M_{calc}.=3489.73 Da, M_{obs}.=3489.52 Da;

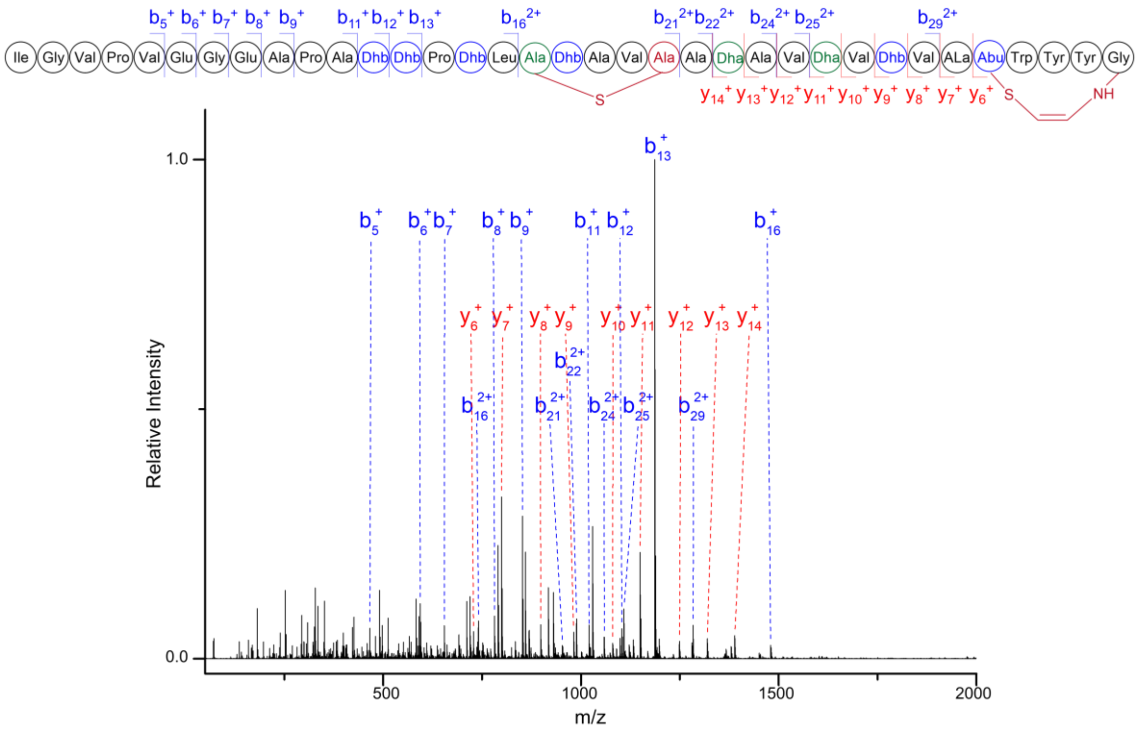

Ions	calc.	obs.	Er. (ppm)	Ions	calc.	obs.	Er. (ppm)
b_5^+	466.3024	466.3011	-2.8	y_6^+	728.2861	728.2848	-1.8
b_6^+	595.3450	595.3434	-2.7	y_7^+	799.3233	799.3217	-2.0
b_7^+	652.3665	652.3651	-2.1	y_8^+	898.3917	898.3893	-2.7
b_8^+	781.4091	781.4072	-2.4	y_9^+	981.4288	981.4267	-2.1
b_9^+	852.4462	852.4443	-2.2	y_{10}^+	1080.4972	1080.4987	1.4
b_{11}^+	1020.5361	1020.5360	-0.1	y_{11}^+	1149.5187	1149.5184	-0.3
b_{12}^+	1103.5732	1103.5721	-1.0	y_{12}^+	1248.5871	1248.5865	-0.5
b_{13}^+	1186.6103	1186.6089	-1.1	y_{13}^+	1319.6242	1319.6245	0.2
b_{16}^+	1479.7843	1479.7834	-0.6	y_{14}^+	1388.6457	1388.6452	-0.4
b_{16}^{2+}	740.3958	740.3944	-1.9				
b_{21}^{2+}	952.9824	952.9811	-1.4				
b_{22}^{2+}	988.5010	988.5009	-0.1				
b_{24}^{2+}	1058.5303	1058.5322	1.8				
b_{25}^{2+}	1108.0645	1108.0639	-0.5				
b_{29}^{2+}	1283.1622	1283.1644	1.7				

Figure S34. LC-MS/MS analysis of RosA1$_{\text{Lan-AviMeCys}}$ after GluC digestion. The *b* and *y* ions are listed in table and marked in the spectrum.

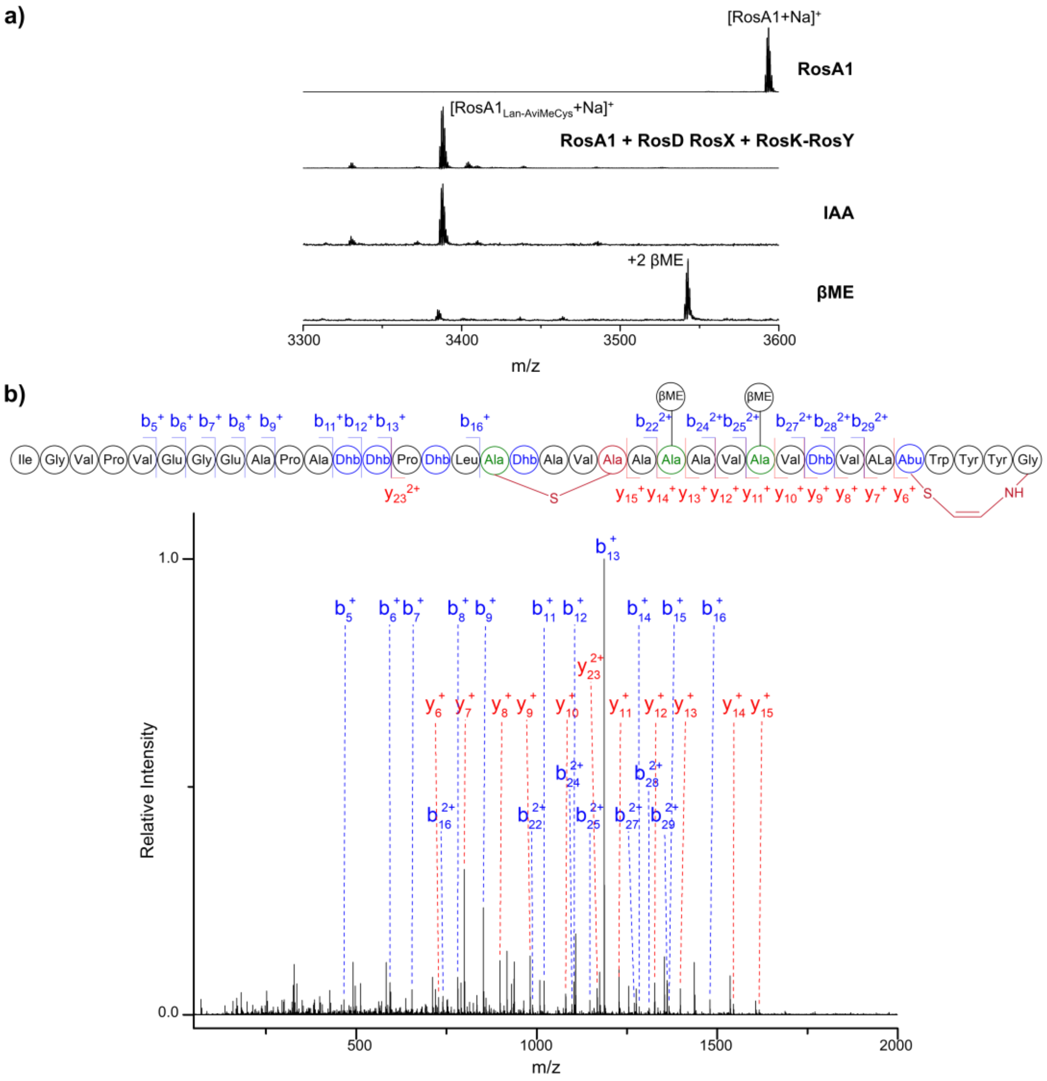

Ions	calc.	obs.	Er. (ppm)	Ions	calc.	obs.	Er. (ppm)
b_5^+	466.3024	466.3022	-0.4	y_6^+	728.2861	728.2866	0.7
b_6^+	595.3450	595.3441	-1.5	y_7^+	799.3233	799.3238	0.6
b_7^+	652.3665	652.3668	0.5	y_8^+	898.3917	898.3926	1.0
b_8^+	781.4091	781.4094	0.4	y_9^+	981.4288	981.4294	0.6
b_9^+	852.4462	852.4474	1.4	y_{10}^+	1080.4972	1080.5001	2.7
b_{11}^+	1020.5361	1020.5374	1.3	y_{11}^+	1227.5326	1227.5333	0.6
b_{12}^+	1103.5732	1103.5732	0	y_{12}^+	1326.6010	1326.6026	1.2
b_{13}^+	1186.6103	1186.6118	1.3	y_{13}^+	1397.6381	1397.6389	0.6
b_{14}^+	1283.6631	1283.6651	1.6	y_{14}^+	1544.6735	1544.6758	1.5
b_{15}^+	1366.7002	1366.7029	2.0	y_{15}^+	1615.7107	1615.7104	-1.9
b_{16}^+	1479.7843	1479.7853	0.7	y_{23}^{2+}	1167.5326	1167.5349	2.0
b_{16}^{2+}	740.3958	740.3955	-0.4				
b_{22}^{2+}	988.5010	988.5039	2.9				
b_{24}^{2+}	1097.5373	1097.5383	0.9				
b_{25}^{2+}	1147.0715	1147.0737	1.9				
b_{27}^{2+}	1270.1234	1270.1241	0.6				
b_{28}^{2+}	1311.6419	1311.6417	-1.5				
b_{29}^{2+}	1361.1761	1361.1800	2.9				

Figure S35. (a). RosD RosX + RosK-RosY (RosD and RosX were added prior to the addition of RosK-RosY) install an AviMeCys motif on RosA1 and MALDI-TOF-MS analysis of the *in vitro* enzymatic modification. [RosA1+Na]$^+$: $M_{calc.}$ = 3593.72 Da, $M_{obs.}$ = 3593.58 Da; [RosA1$_{Lan\text{-}AviMeCys}$+Na]$^+$: $M_{calc.}$ = 3385.61 Da, $M_{obs.}$ = 3386.15 Da; [RosA1$_{Lan\text{-}AviMeCys}$+2 βME+Na]$^+$: $M_{calc.}$=3541.64 Da, $M_{obs.}$=3540.89 Da; (b). LC-MS/MS analysis of RosA1$_{Lan\text{-}AviMeCys}$-βME adduct after GluC digestion. The *b* and *y* ions are listed in table and marked in the spectrum.

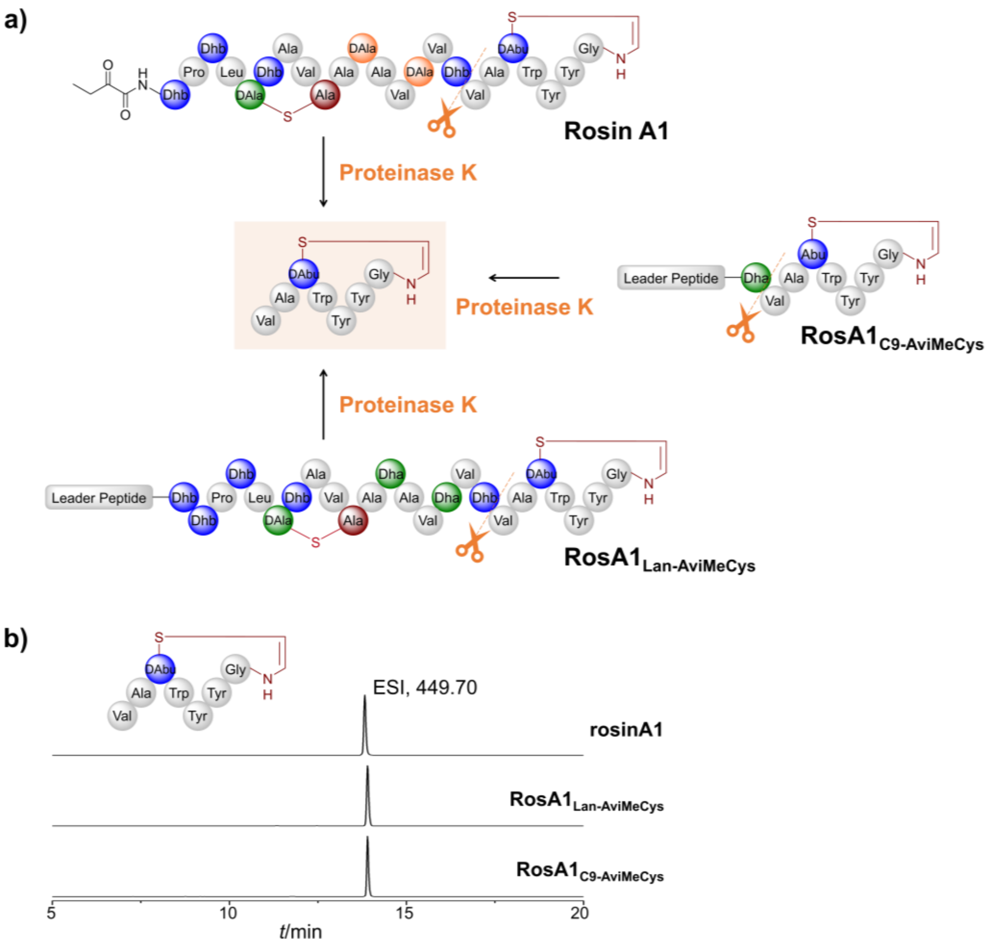

Figure S36. (a). Schematic diagram of proteinase K digestion of $RosA1_{Lan\text{-}AviMeCys}$, $RosA1_{C9\text{-}AviMeCys}$ and rosinA1. (b). EIC signals of AviMeCys rings derived from $RosA1_{Lan\text{-}AviMeCys}$, $RosA1_{C9\text{-}AviMeCys}$ and rosinA1 after proteinase K digestion.

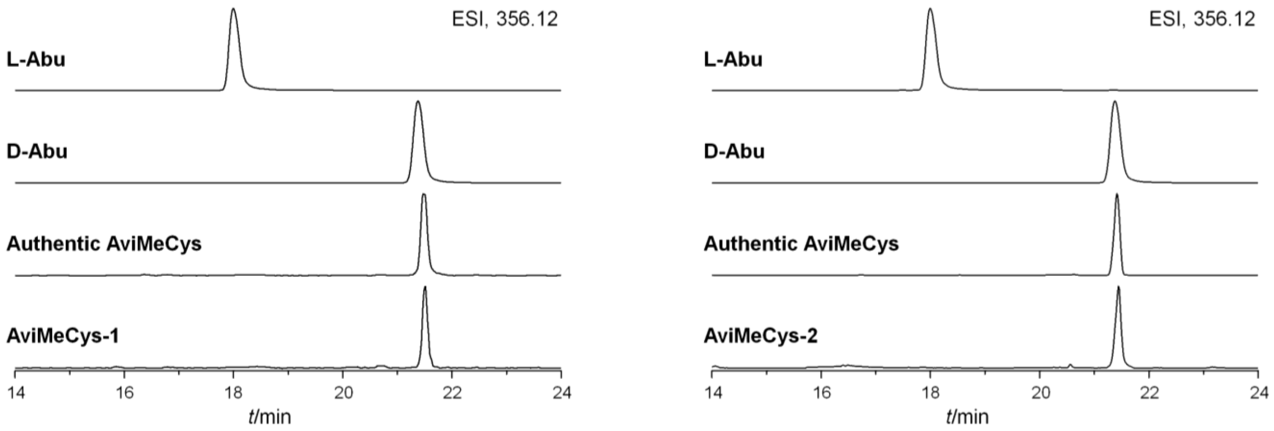

Figure S37. Marfey's analysis of the Abu residue derived from the hydrolysis of the AviMeCys motif in RosA1$_{\text{Lan-AviMeCys}}$ (designated AviMeCys-1) and RosA1$_{\text{C9-AviMeCys}}$ (AviMeCys-2), with the authentic AviMeCys motif from rosin A1 as a control.

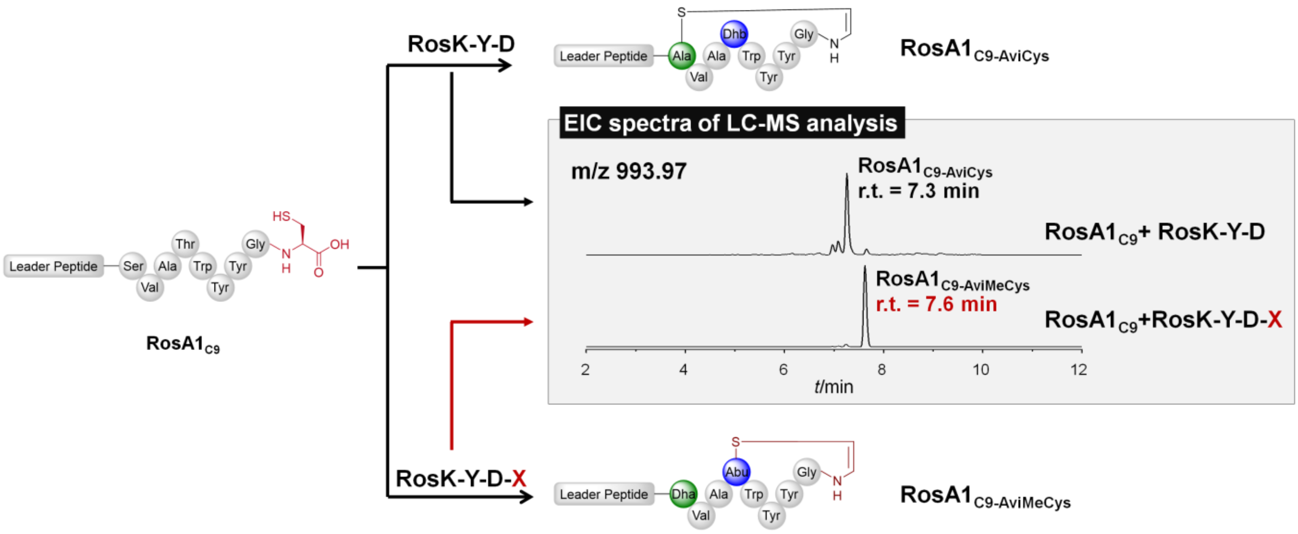

Figure S38. LC-MS analysis of modifications of RosA1C9 by RosK-Y-D and RosK-Y-D-X. EIC profiles of the fully modified products are shown ($[M+2H]^{2+}$ = 993.97).

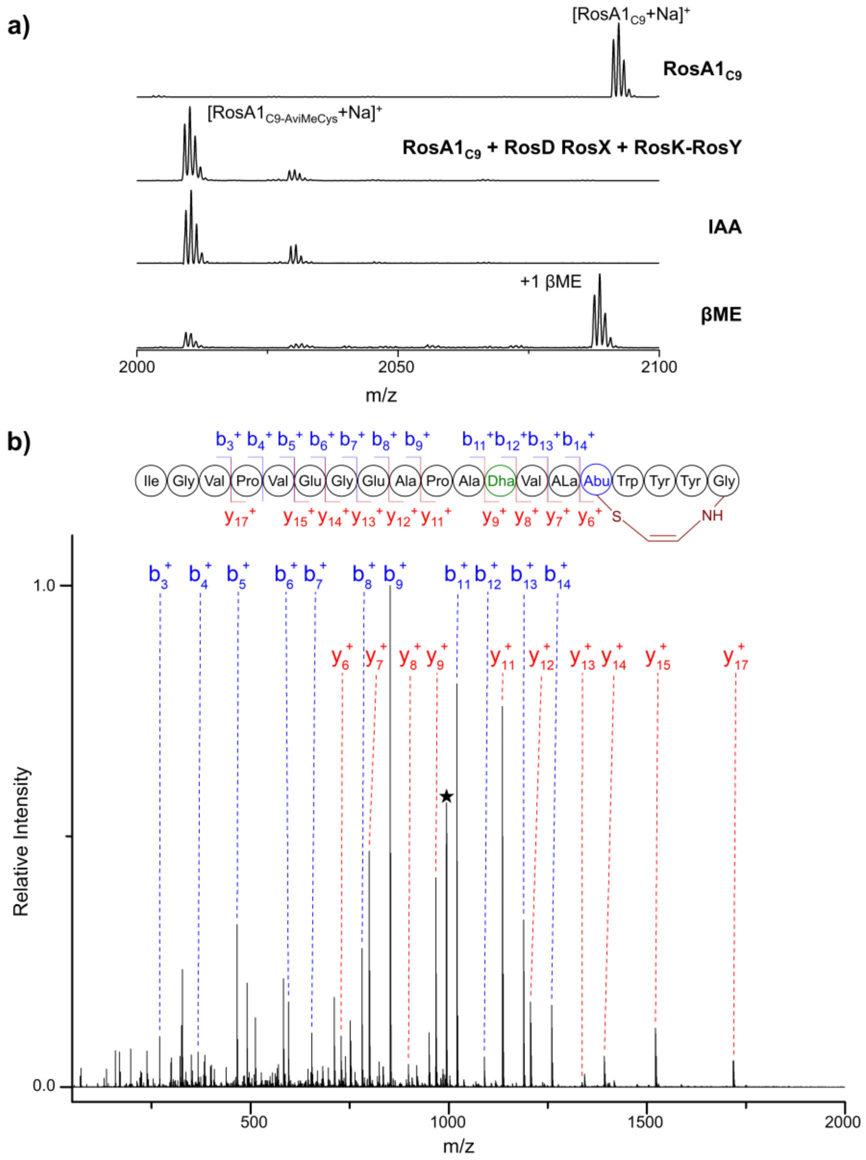

Ions	calc.	obs.	Er. (ppm)	Ions	calc.	obs.	Er. (ppm)
b_3^+	270.1813	270.1812	-0.4	y_6^+	728.2861	728.2854	-1.0
b_4^+	367.2340	367.2331	-2.5	y_7^+	799.3233	799.3223	-1.3
b_5^+	466.3024	466.3017	-1.5	y_8^+	898.3917	898.3927	1.1
b_6^+	595.3450	595.3440	-1.7	y_9^+	967.4131	967.4122	-0.9
b_7^+	652.3665	652.3650	-2.3	y_{11}^+	1135.5030	1135.5016	-1.2
b_8^+	781.4091	781.4079	-1.5	y_{12}^+	1206.5401	1206.5393	-0.7
b_9^+	852.4462	852.4448	-1.6	y_{13}^+	1335.5827	1335.5812	-1.1
b_{11}^+	1020.5361	1020.5348	-1.3	y_{14}^+	1392.6042	1392.6020	-1.6
b_{12}^+	1089.5576	1089.5557	-1.7	y_{15}^+	1521.6468	1521.6457	-0.7
b_{13}^+	1188.6260	1188.6254	-0.5	y_{17}^+	1717.7680	1717.7650	-1.7
b_{14}^+	1259.6631	1259.6619	-1.0				

Figure S39. (a). MALDI-TOF-MS analysis of the *in vitro* enzymatic modification of RosA1$_{C9}$ by RosD RosX + RosK-RosY (RosD and RosX were added prior to the addition of RosK-RosY. (b). LC-MS/MS analysis of RosA1$_{C9\text{-}AviMeCys}$ after GluC digestion. The *b* and *y* ions are listed in table and marked in the spectrum. The asterisk indicates $[M+2H]^{2+}$.
[RosA1$_{C9}$+Na]$^+$: $M_{calc.}$ = 2090.95 Da, $M_{obs.}$ = 2091.24 Da;
[RosA1$_{C9\text{-}AviMeCys}$+Na]$^+$: $M_{calc.}$ = 2008.92 Da, $M_{obs.}$ = 2009.40 Da;
[RosA1$_{C9\text{-}AviMeCys}$+βME+Na]$^+$: $M_{calc.}$=2086.94 Da, $M_{obs.}$=2087.57 Da;

Substrate	Sequence	K_D (μM)
RosA1$_{leader}$	MDTTEQLIAGYTAYTDAEEIGVPVEGEAPA	≥ 6 ± 2
RosA1$_{-25--1}$	QLIAGYTAYTDAEEIGVPVEGEAPA	≥ 11 ± 5
RosA1$_{-20--1}$	YTAYTDAEEIGVPVEGEAPA	≥ 25 ± 7
RosA1$_{-15--1}$	DAEEIGVPVEGEAPA	≥ 70 ± 25

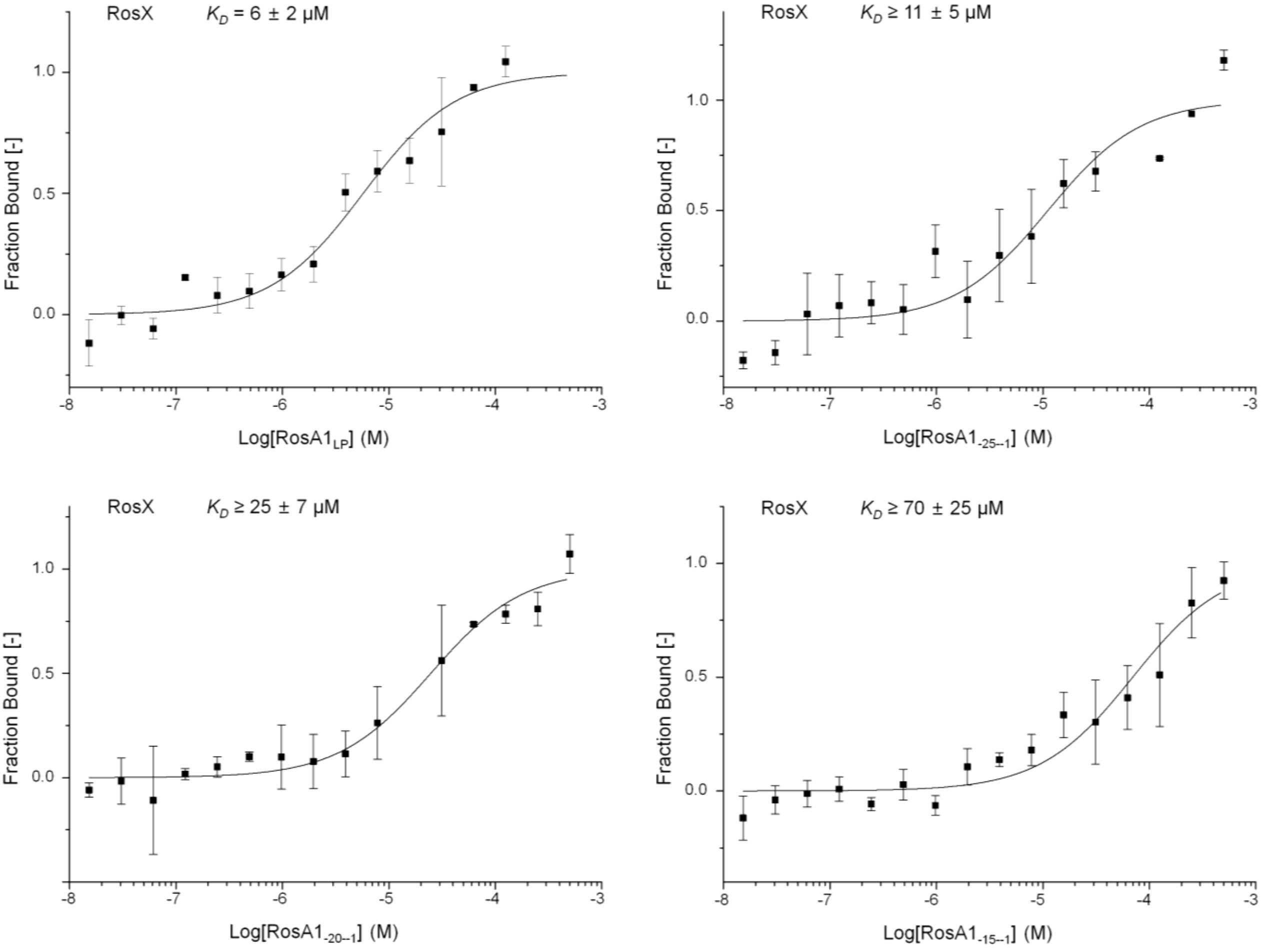

Figure S40. Binding affinities between RosX and RosA1$_{leader}$ variants measured by MST. Data represent the mean ± s.d. from three replicates.

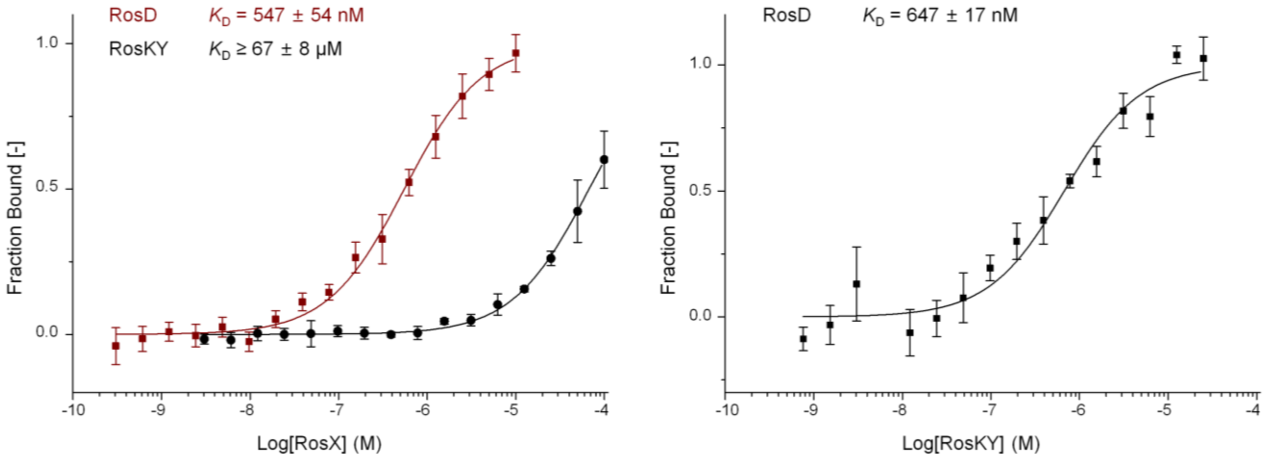

Figure S41. Binding affinities between RosX and RosD, RosX and RosKY, RosKY and RosD. Data represent the mean ± s.d. from three replicates.

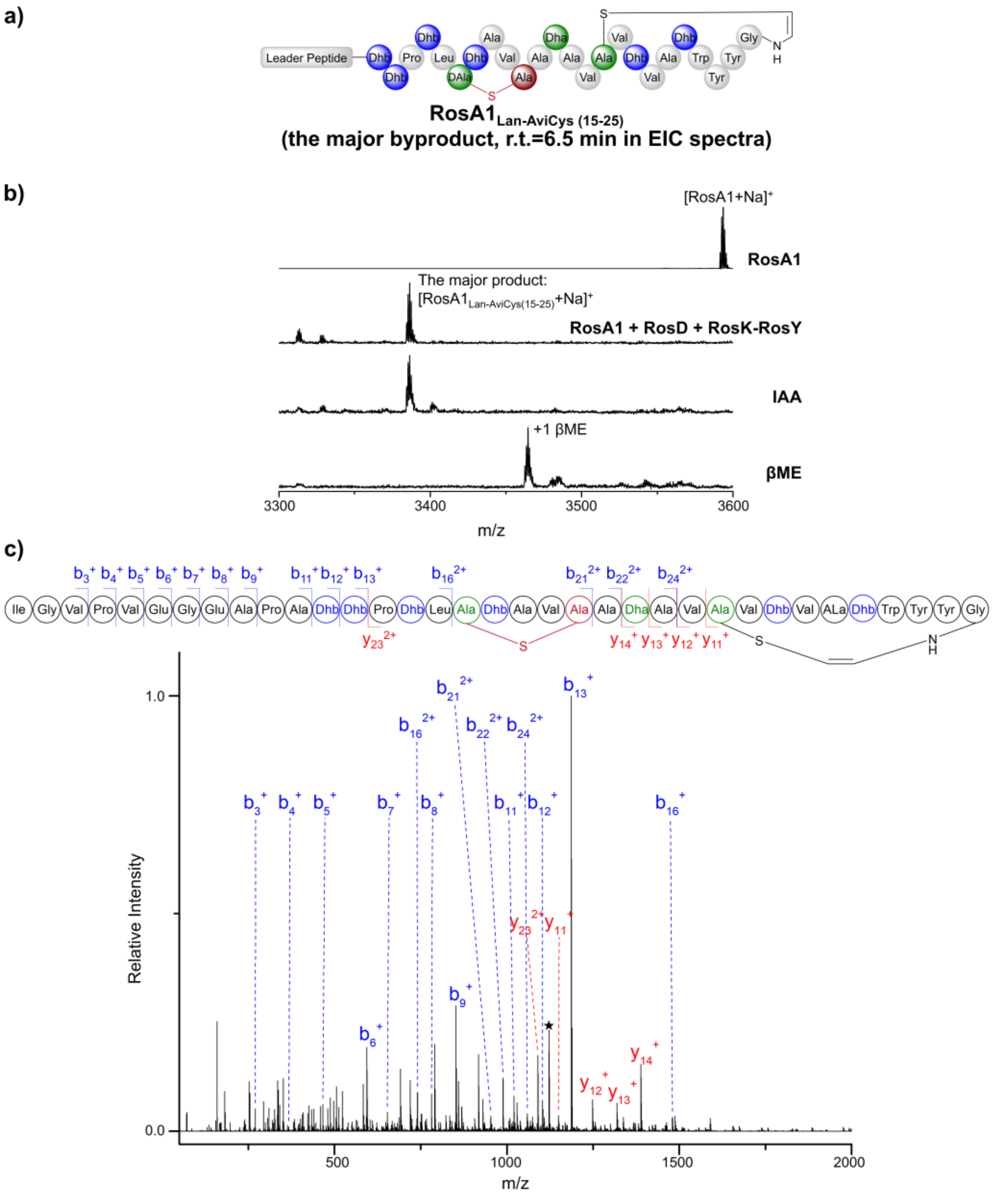

Ions	calc.	obs.	Er. (ppm)	Ions	calc.	obs.	Er. (ppm)
b_3^+	270.1813	270.1814	3.7	y_{11}^+	1149.5187	1149.5182	-0.4
b_4^+	367.2340	367.2338	-0.5	y_{12}^+	1248.5871	1248.5884	1.0
b_5^+	466.3024	466.3032	1.7	y_{13}^+	1319.6242	1319.6265	1.7
b_6^+	595.3450	595.3447	-0.5	y_{14}^+	1388.6457	1388.6446	-0.8
b_7^+	652.3665	652.3675	1.5	y_{23}^{2+}	1089.5187	1089.5212	2.3
b_8^+	781.4091	781.4103	1.5				
b_9^+	852.4462	852.4479	2.0				
b_{11}^+	1020.5361	1020.5377	1.6				
b_{12}^+	1103.5732	1103.5751	1.7				
b_{13}^+	1186.6103	1186.6111	0.7				
b_{16}^+	1479.7843	1479.7857	0.9				
b_{16}^{2+}	740.3958	740.3971	1.8				
b_{21}^{2+}	952.9824	952.9851	2.8				
b_{22}^{2+}	988.5010	988.5026	1.6				
b_{24}^{2+}	1058.5303	1058.5341	3.6				

Figure S42. MS analysis of byproduct RosA1$_{\text{Lan-AviCys(15-25)}}$. (a). The structure of RosA1$_{\text{Lan-AviCys(15-25)}}$. (b). MALDI-TOF-MS analysis of the modification of RosA1 by RosK-RosY-RosD and subsequent derivatization. [RosA1$_{\text{Lan-AviCys(15-25)}}$+Na]$^+$: $M_{\text{calc.}}$ = 3385.61 Da, $M_{\text{obs.}}$ = 3384.84 Da; [RosA1$_{\text{Lan-AviCys(15-25)}}$+1 βME+Na]$^+$: $M_{\text{calc.}}$=3463.63 Da, $M_{\text{obs.}}$=3462.78 Da; (c). LC-MS/MS analysis of RosA1$_{\text{Lan-AviCys(15-25)}}$ after GluC digestion. The *b* and *y* ions are listed in table and marked in the spectrum. The asterisk indicates [M+3H]$^{3+}$.

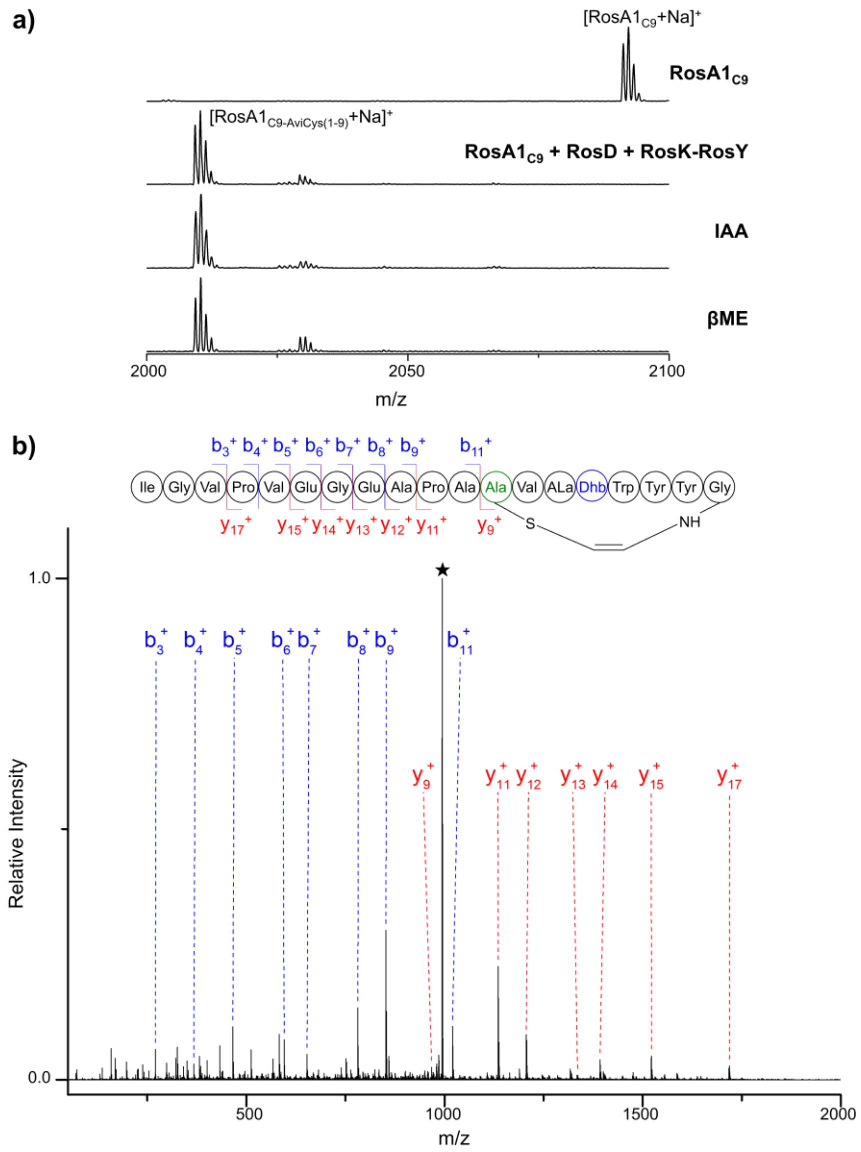

Ions	calc.	obs.	Er. (ppm)	Ions	calc.	obs.	Er. (ppm)
b_3^+	270.1813	270.1815	0.7	y_9^+	967.4131	967.4156	2.6
b_4^+	367.2340	367.2341	0.3	y_{11}^+	1135.5030	1135.5046	1.4
b_5^+	466.3024	466.3017	-1.5	y_{12}^+	1206.5401	1206.5432	2.6
b_6^+	595.3450	595.3441	-1.5	y_{13}^+	1335.5827	1335.5828	0.1
b_7^+	652.3665	652.3677	1.8	y_{14}^+	1392.6042	1392.6058	1.1
b_8^+	781.4091	781.4098	0.9	y_{15}^+	1521.6468	1521.6484	1.1
b_9^+	852.4462	852.4468	0.7	y_{17}^+	1717.7680	1717.7712	1.9
b_{11}^+	1020.5361	1020.5377	1.6				

Figure S43. (a). MALDI-TOF-MS analysis of the *in vitro* enzymatic modification of RosA1$_{C9}$ by RosD + RosK-RosY (RosD was added prior to the addition of RosK-RosY). [RosA1$_{C9}$+Na]$^+$: $M_{calc.}$ = 2090.95 Da, $M_{obs.}$ = 2091.24 Da; [RosA1$_{C9\text{-AviCys}(1\text{-}9)}$+Na]$^+$: $M_{calc.}$ = 2008.92 Da, $M_{obs.}$ = 2009.28 Da; (b). LC-MS/MS analysis of RosA1$_{C9\text{-AviCys}(1\text{-}9)}$ after GluC digestion. The *b* and *y* ions are listed in table and marked in the spectrum. The asterisk indicates [M+2H]$^{2+}$.

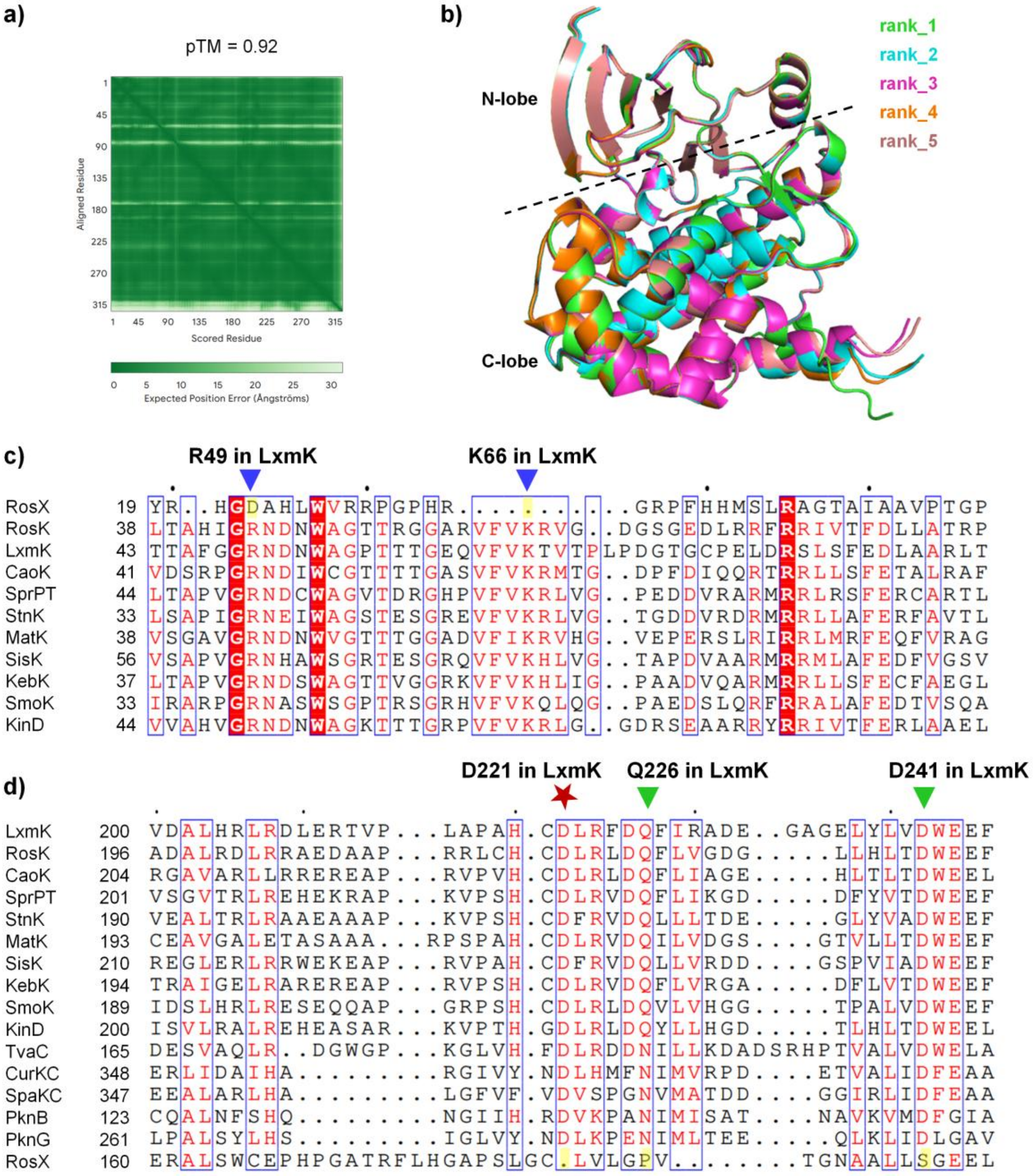

Figure S44. (a). The predicted template modeling (pTM) score of RosX predicted structures. (b). Overlay of the highest-ranking five models of RosX. (c). Sequence alignment of RosX with LanKs reveals the absence of putative ATP binding motifs in RosX. Numeric labels on the left indicate the position of the first amino acid in the respective protein sequence. Residues potentially involved in ATP binding are highlighted with blue triangles. (d). Sequence alignment of RosX with representative kinases (TvaC$_{S-87}$,[20] CurKC,[21] SpaKC,[22] PknB,[23] and PknG[24]) demonstrating the absence of conserved catalytic and Mg^{2+}-coordinating residues in RosX. The conserved catalytic residue Asp is indicated by red star. Mg^{2+}-chelating residues Asn and Asp are highlighted with green triangles.

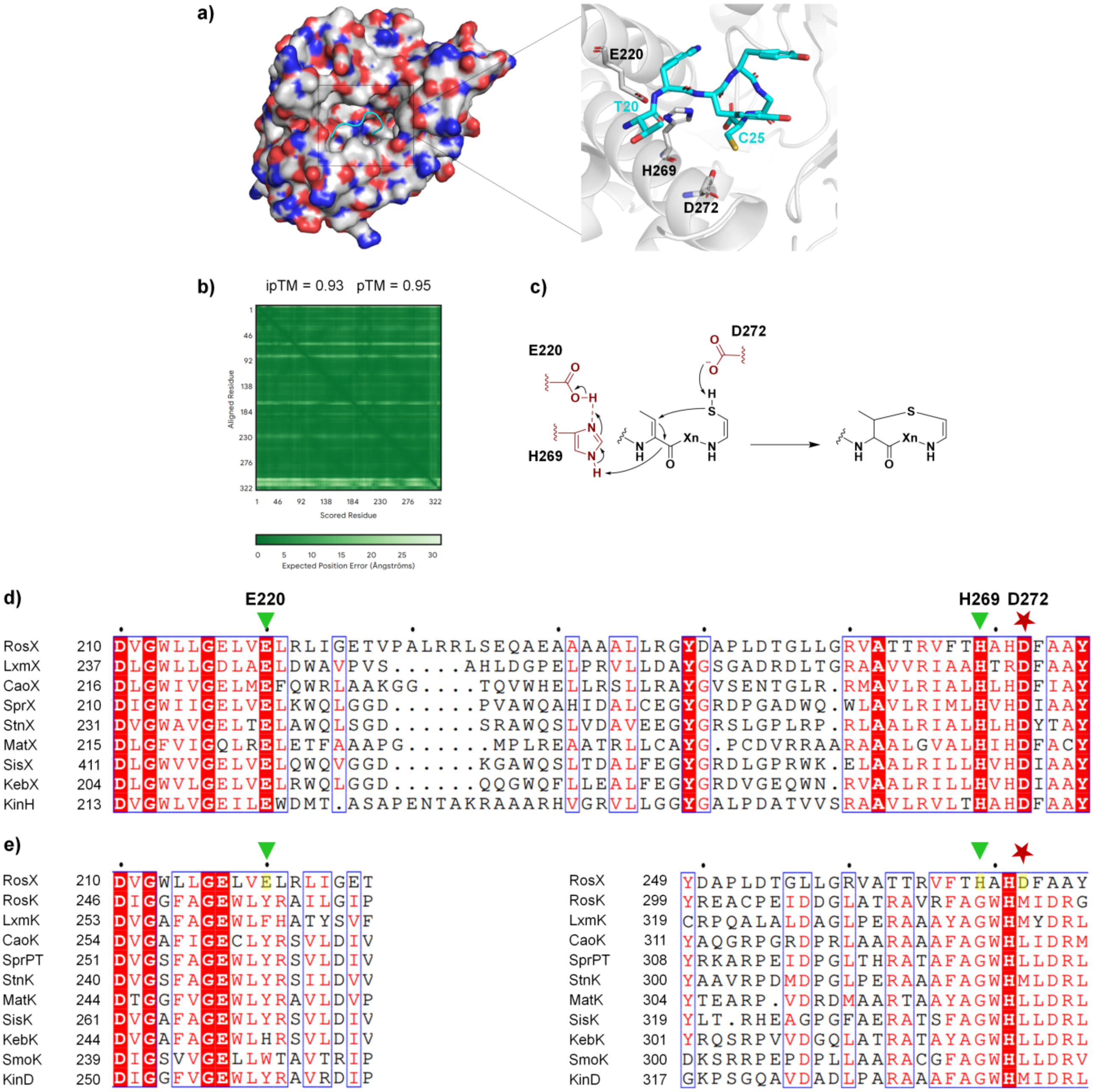

Figure S45. (a). Overall structure of the rank_1 model of RosX-RosA1$_{C6}$ and potential catalytic residues. (b). The predicted template modeling (pTM) score and the interface predicted template modeling (ipTM) score of the RosX-RosA1$_{C6}$ complex model. (c). A possible cyclization mechanism of the AviMeCys crosslinking. (d). Sequence alignment of RosX with LanXs reveals the high conservation of Glu220, His269 and Asp272. Numeric labels on the left indicate the position of the first amino acid in the respective protein sequence. (e). Sequence alignment analysis demonstrated that LanKs lack the conserved Glu220, His269, and Asp272 residues present in RosX.

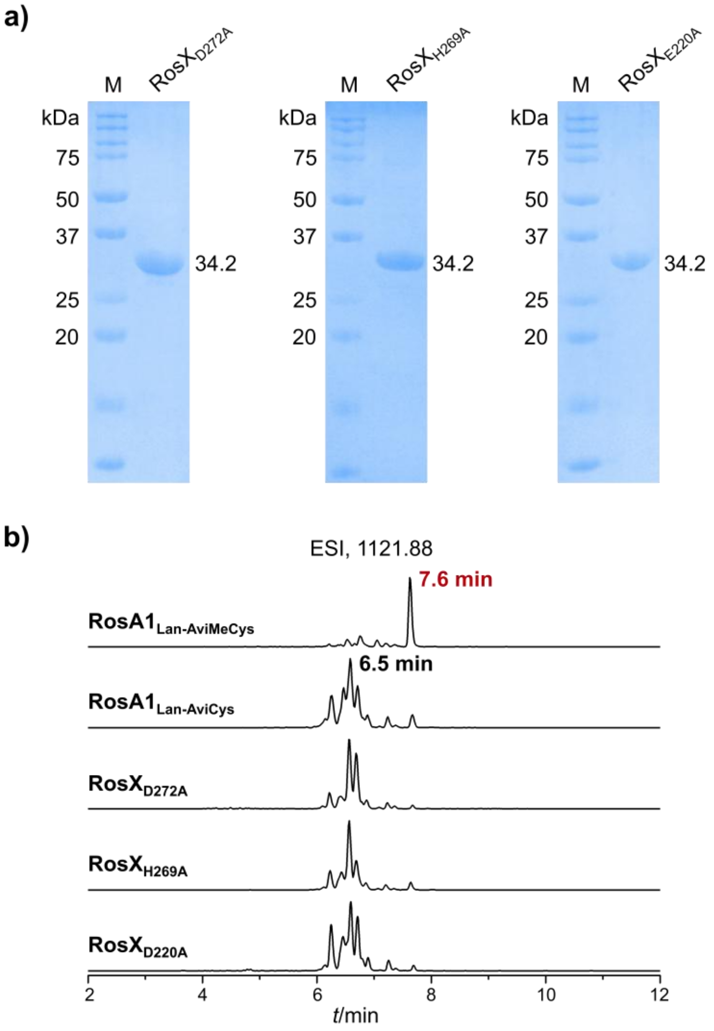

Figure S46. (a). SDS-PAGE analysis of RosX$_{H269A}$. (b). EIC spectra of the mass of fully dehydrated and decarboxylated RosA1 peptide ($[M+3H]^{3+}$ = 1121.88). Reaction condition: His$_6$-RosA1 (100 μM) and His$_6$-RosD (20 μM), RosX$_{mutants}$ (20 μM) were incubated with 5 mM ATP, 1 mM $MgCl_2$, 5 μM FMN and 1 mM DTT in 50 mM HEPES, pH 8.0, at 28°C for 10 min, His$_6$-RosK-RosY complex (20 μM) was then added to proceed for an additional 4 h.

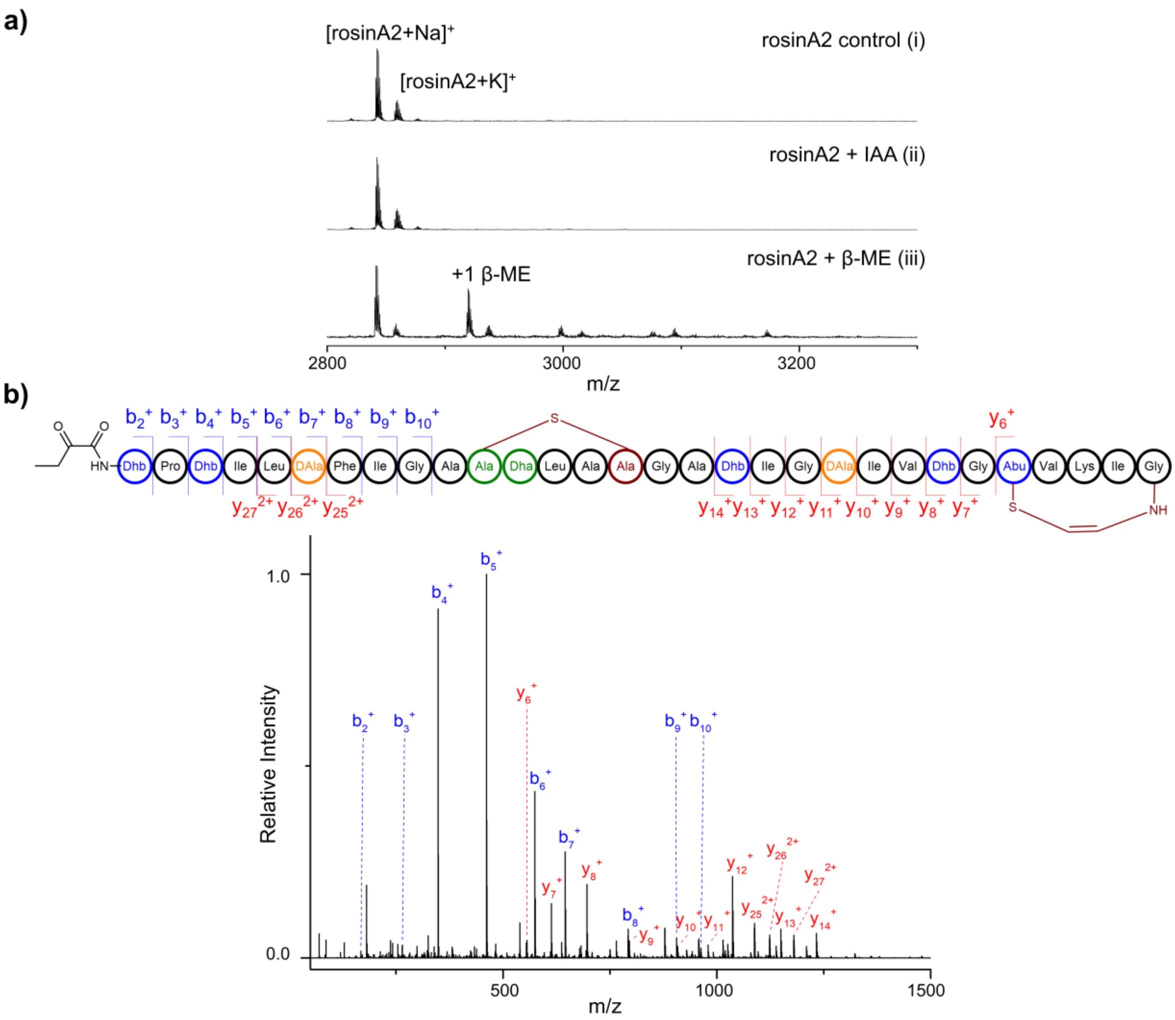

Ions	calc.	obs.	Er. (ppm)	Ions	calc.	obs.	Er. (ppm)
b_2^+	168.0655	168.0653	-1.2	y_6^+	556.3276	556.3258	-3.2
b_3^+	265.1183	265.1180	-1.1	y_7^+	613.3490	613.3498	1.3
b_4^+	348.1554	348.1551	-0.9	y_8^+	696.3867	696.3878	1.6
b_5^+	461.2395	461.2401	1.3	y_9^+	795.4546	795.4507	-4.9
b_6^+	574.3235	574.3240	0.9	y_{10}^+	908.5386	908.5358	-3.1
b_7^+	645.3606	645.3626	3.1	y_{11}^+	979.5757	979.5739	-1.8
b_8^+	792.4291	792.4298	0.9	y_{12}^+	1036.5972	1036.5977	0.5
b_9^+	905.5131	905.5105	-2.9	y_{13}^+	1149.6813	1149.6784	-2.5
b_{10}^+	962.5346	962.5378	3.3	y_{14}^+	1232.7184	1232.7149	-2.8
				y_{25}^{2+}	1087.5843	1087.5842	-0.1
				y_{26}^{2+}	1123.1028	1123.1034	0.5
				y_{27}^{2+}	1179.6449	1179.6460	0.9

Figure S47. MS and MS/MS analysis of rosin A2. (a). MALDI-TOF-MS analysis of rosin A2 treated with IAA and β-ME. Assay conditions: (i) Rosin A2 was incubated in 20 mM Tris-HCl, pH 8.0, for 1 hour at room temperature. [rosinA2+Na]$^+$: $M_{obs.}$ = 2840.49 Da, $M_{calc.}$ = 2840.50 Da. [M+K]$^+$: $M_{obs.}$ = 2856.44 Da, $M_{calc.}$ = 2856.47 Da. (ii) Rosin A2 was incubated in 20 mM Tris-HCl, pH 8.0, 0.5 mM TCEP, with 1 mM IAA for 30 min at room temperature. (iii) Rosin A2 was incubated in 20 mM Tris-HCl, pH 8.0, with 0.5 mM βME for 1 hour at 37°C. [rosinA2+βME+Na]$^+$: $M_{obs.}$ = 2918.52 Da, $M_{calc.}$ = 2918.51 Da. (b). LC-MS/MS analysis of rosin A2. The *b* and *y* ions are listed in table and marked in the spectrum.

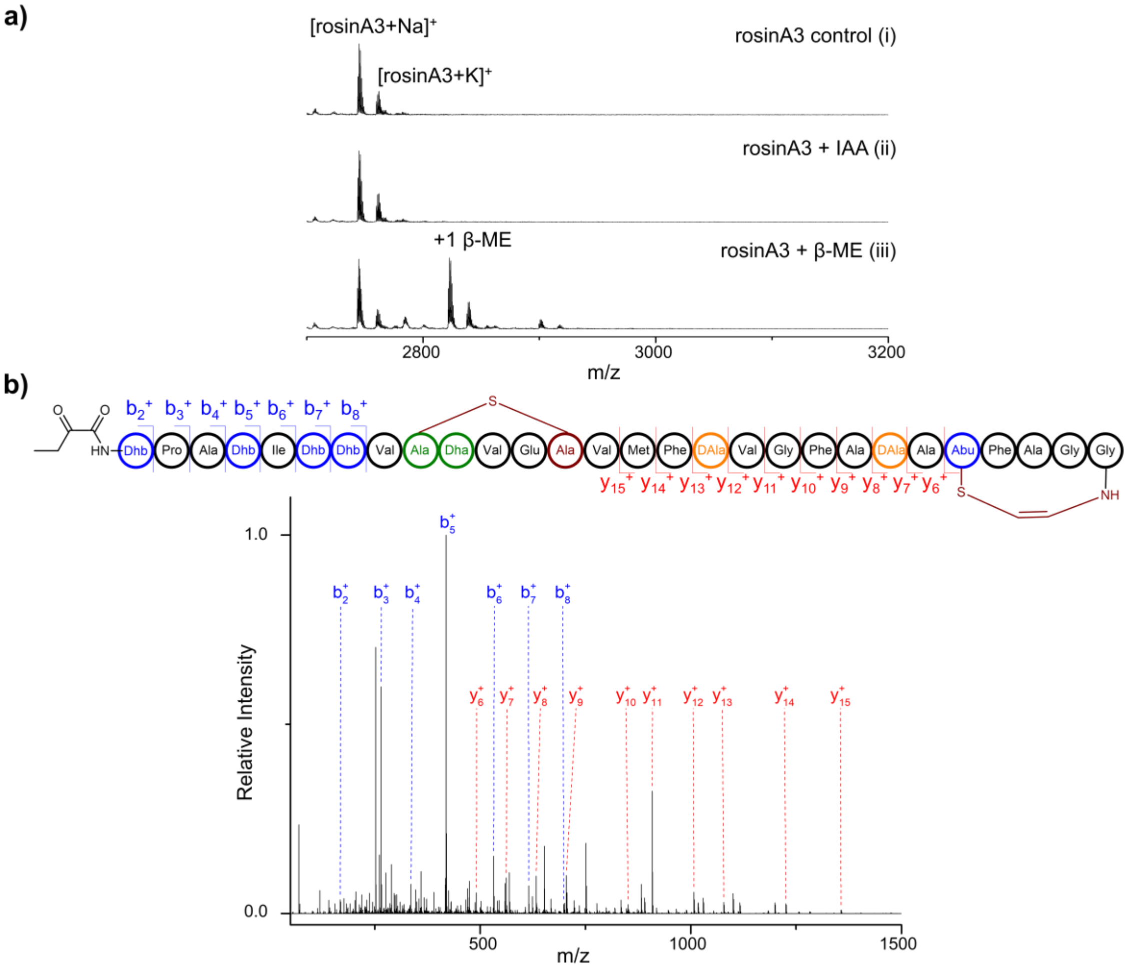

Ions	calc.	obs.	Er. (ppm)	Ions	calc.	obs.	Er. (ppm)
b_2^+	168.0656	168.0659	1.8	y_6^+	491.2072	491.2078	1.2
b_3^+	265.1183	265.1188	1.9	y_7^+	562.2443	562.2449	1.1
b_4^+	336.1555	336.1559	1.2	y_8^+	633.2814	633.2824	1.6
b_5^+	419.1926	419.1924	-0.5	y_9^+	704.3185	704.3197	1.7
b_6^+	532.2766	532.2769	0.6	y_{10}^+	851.3869	851.3885	1.9
b_7^+	615.3138	615.3142	0.7	y_{11}^+	908.4084	908.4098	1.5
b_8^+	698.3509	698.3514	0.7	y_{12}^+	1007.4768	1007.4792	2.4
				y_{13}^+	1078.5139	1078.5161	2.0
				y_{14}^+	1225.5823	1225.5855	2.6
				y_{15}^+	1356.6228	1356.6261	2.4

Figure S48. MS and MS/MS analysis of rosin A3. (a). MALDI-TOF-MS analysis of rosin A3 treated with IAA and β-ME. Assay conditions: (i) Rosin A3 was incubated in 20 mM Tris-HCl, pH 8.0, for 1 hour at room temperature. [rosinA3+Na]$^+$: $M_{obs.}$ = 2744.03 Da, $M_{calc.}$ = 2744.28 Da. [M+K]$^+$: $M_{obs.}$ = 2760.03 Da, $M_{calc.}$ = 2760.25 Da. (ii) Rosin A3 was incubated in 20 mM Tris-HCl, pH 8.0, 0.5 mM TCEP, with 1 mM IAA for 30 min at room temperature. (iii) Rosin A3 was incubated in 20 mM Tris-HCl, pH 8.0, with 0.5 mM βME for 1 hour at 37°C. [rosinA3+βME+Na]$^+$: $M_{obs.}$ = 2921.83 Da, $M_{calc.}$ = 2822.29 Da. (b). LC-MS/MS analysis of rosin A3. The *b* and *y* ions are listed in table and marked in the spectrum.

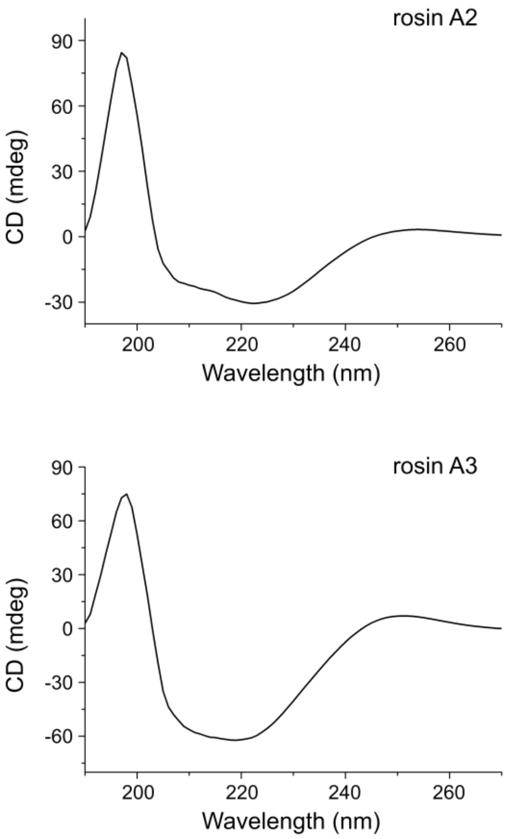

Figure S49. The circular dichroism spectras of rosin A2/A3 in MeOH at 298 K.

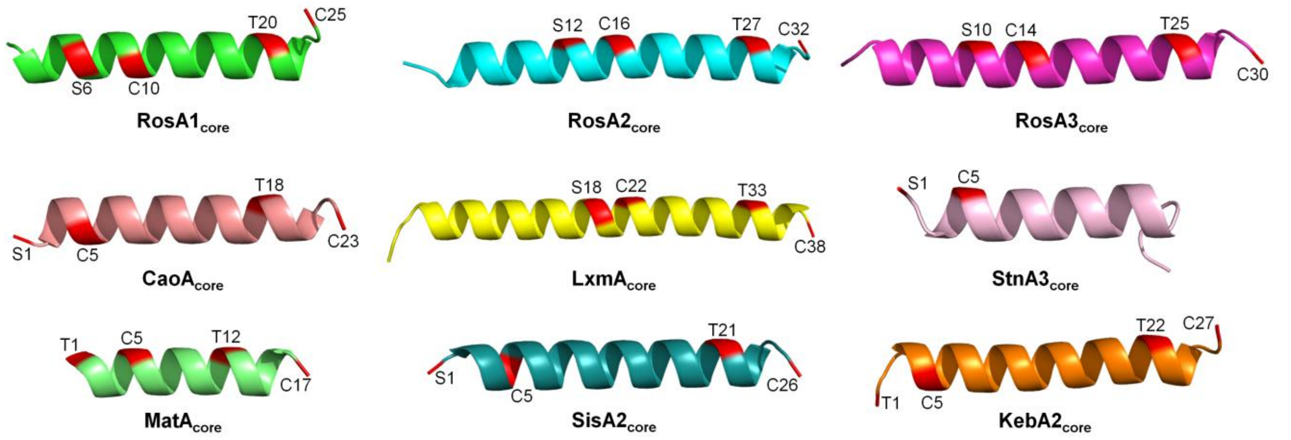

Figure S50. The rank_1 models from AlphaFold3 prediction for the core peptides of known class V_a lanthipeptides. The amino acids involved in the cyclization are marked in red.

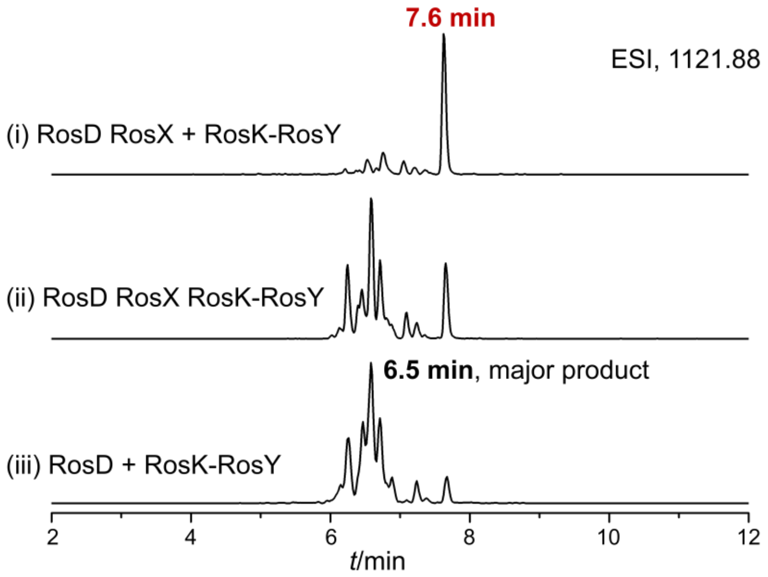

Figure S51. EIC signals of $[M+3H]^{3+}$ = 1121.8, which is the mass of fully dehydrated and decarboxylated RosA1 peptide, of enzymatic reactions with varied order of enzyme addition.
(i) RosD and RosX were added to the solution of RosA1 10 min prior to the addition of the RosK-RosY complex;
(ii) RosD, RosX and the RosK-RosY complex were added simultaneously;
(iii) RosD was added to the solution of RosA1 10 min prior to the addition of the RosK-RosY complex.
Reaction condition: His_6-RosA1 (100 μM), His_6-RosD (20 μM), RosX (20 μM), His_6-RosK-RosY complex (20 μM), 5 mM ATP, 1 mM $MgCl_2$, 5 μM FMN, 1 mM DTT, 50 mM HEPES, pH 8.0, 28°C.

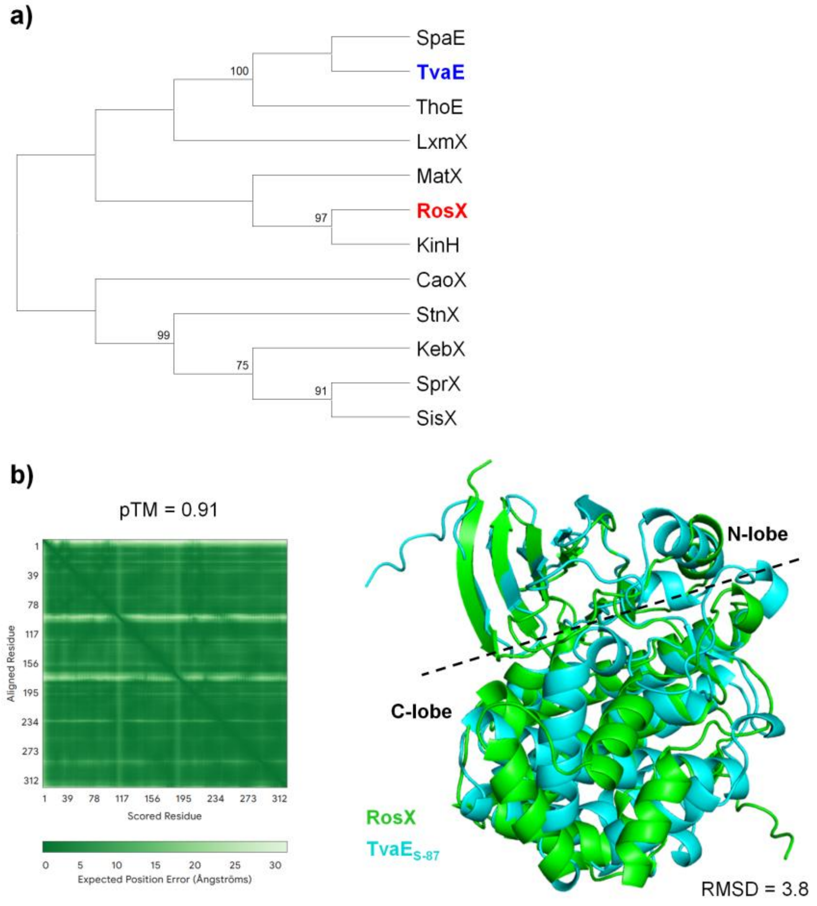

Figure S52. (a). The maximum likelihood phylogenetic analysis of TvaE$_{S-87}$ in comparison with other LanXs involved in the biosynthesis of class V lanthipeptides and thioamitides. Only bootstrap values greater than 70 are shown on the trees. (b). The predicted template modeling (pTM) score of TvaE$_{S-87}$ predicted structures and the highest-confidence predicted structural model superposition with RosX.

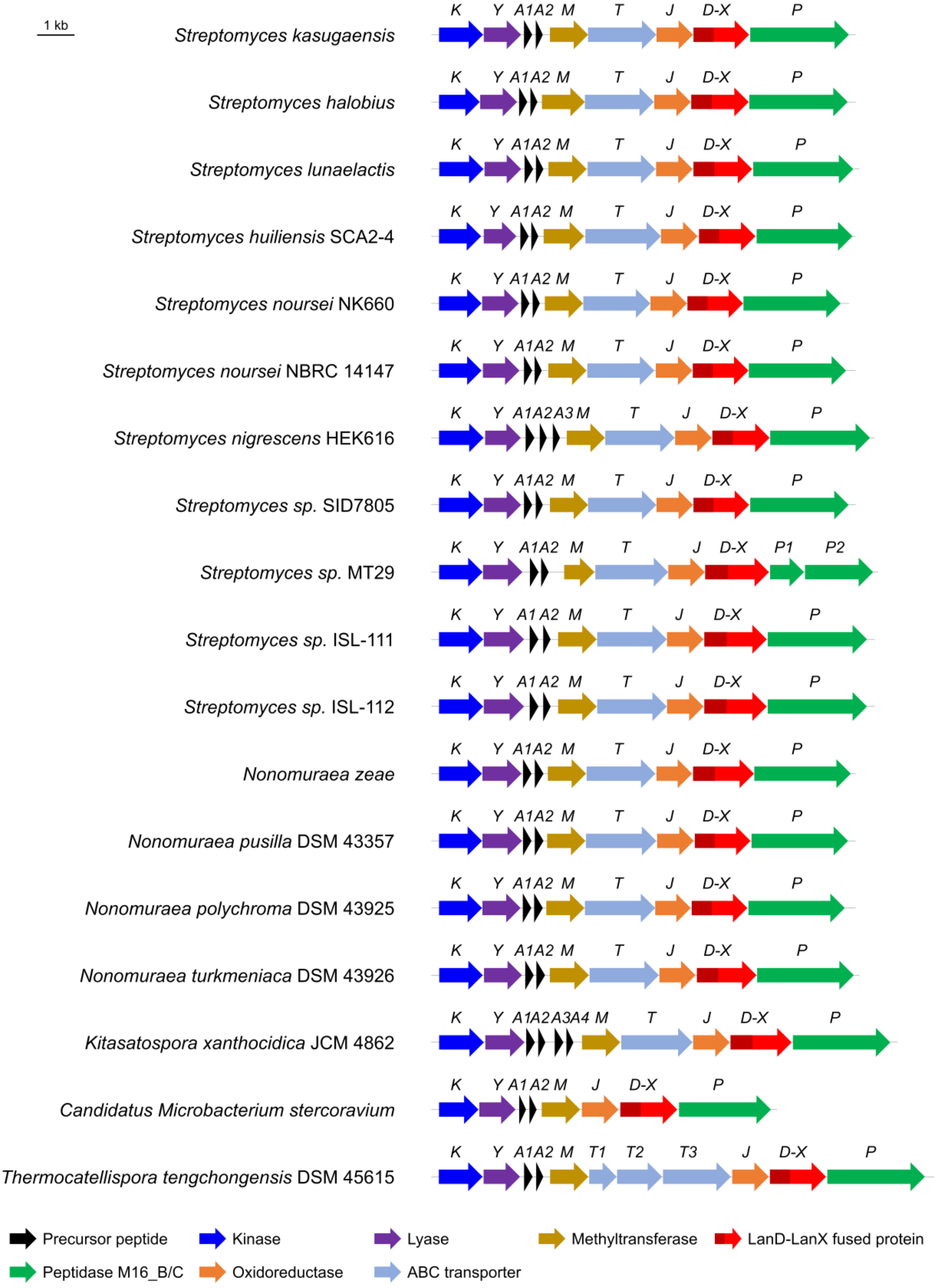

Figure S53. Representative class V lanthipeptide BGCs containing LanD-LanX fused protein.

References

1. Montalbán-López, M.; Scott, T. A.; Ramesh, S.; Rahman, I. R.; van Heel, A. J.; Viel, J. H.; Bandarian, V.; Dittmann, E.; Genilloud, O.; Goto, Y.; Grande Burgos, M. J.; Hill, C.; Kim, S.; Koehnke, J.; Latham, J. A.; Link, A. J.; Martínez, B.; Nair, S. K.; Nicolet, Y.; Rebuffat, S.; Sahl, H.-G.; Sareen, D.; Schmidt, E. W.; Schmitt, L.; Severinov, K.; Süssmuth, R. D.; Truman, A. W.; Wang, H.; Weng, J.-K.; van Wezel, G. P.; Zhang, Q.; Zhong, J.; Piel, J.; Mitchell, D. A.; Kuipers, O. P.; van der Donk, W. A., New Developments in Ripp Discovery, Enzymology and Engineering. *Natural Product Reports* **2021,** *38* (1), 130-239.
2. Gilchrist, C. L. M.; Booth, T. J.; van Wersch, B.; van Grieken, L.; Medema, M. H.; Chooi, Y. H., Cblaster: A Remote Search Tool for Rapid Identification and Visualization of Homologous Gene Clusters. *Bioinform Adv* **2021,** *1* (1), vbab016.
3. Grant-Mackie, E. S.; Williams, E. T.; Harris, P. W. R.; Brimble, M. A., Aminovinyl Cysteine Containing Peptides: A Unique Motif That Imparts Key Biological Activity. *JACS Au* **2021,** *1* (10), 1527-1540.
4. Cheng, Z.; He, B.-B.; Lei, K.; Gao, Y.; Shi, Y.; Zhong, Z.; Liu, H.; Liu, R.; Zhang, H.; Wu, S.; Zhang, W.; Tang, X.; Li, Y.-X., Rule-based Omics Mining Reveals Antimicrobial Macrocyclic Peptides Against Drug-Resistant Clinical Isolates. *Nature Communications* **2024,** *15* (1), 4901.
5. Ding, W.; Wang, X.; Yin, Y.; Tao, J.; Xue, Y.; Liu, W., Characterization of a LanC-free Pathway for the Formation of an ll-MeLan Residue and an alloAviMeCys Residue in the Newly Identified Class V Lanthipeptide Triantimycins. *Chemical Science* **2024,** *15* (24), 9266-9273.
6. Ortiz-López, F. J.; Carretero-Molina, D.; Sánchez-Hidalgo, M.; Martín, J.; González, I.; Román-Hurtado, F.; de la Cruz, M.; García-Fernández, S.; Reyes, F.; Deisinger, J. P.; Müller, A.; Schneider, T.; Genilloud, O., Cacaoidin, First Member of the New Lanthidin RiPP Family. *Angewandte Chemie International Edition* **2020,** *59* (31), 12654-12658.
7. Xu, M.; Zhang, F.; Cheng, Z.; Bashiri, G.; Wang, J.; Hong, J.; Wang, Y.; Xu, L.; Chen, X.; Huang, S.-X.; Lin, S.; Deng, Z.; Tao, M., Functional Genome Mining Reveals a Class V Lanthipeptide Containing a D-Amino Acid Introduced by an $F_{420}H_2$-Dependent Reductase. *Angewandte Chemie International Edition* **2020,** *59* (41), 18029-18035.
8. Tietz, J. I.; Schwalen, C. J.; Patel, P. S.; Maxson, T.; Blair, P. M.; Tai, H.-C.; Zakai, U. I.; Mitchell, D. A., A New Genome-Mining Tool Redefines the Lasso Peptide Biosynthetic Landscape. *Nature Chemical Biology* **2017,** *13* (5), 470-478.
9. Tamura, K.; Stecher, G.; Kumar, S., MEGA11: Molecular Evolutionary Genetics Analysis Version 11. *Mol Biol Evol* **2021,** *38* (7), 3022-3027.
10. Thompson, J. D.; Higgins, D. G.; Gibson, T. J., CLUSTAL W: Improving the Sensitivity of Progressive Multiple Sequence Alignment through Sequence Weighting, Position-Specific Gap Penalties and Weight Matrix Choice. *Nucleic Acids Res* **1994,** *22* (22), 4673-80.
11. Robert, X.; Gouet, P., Deciphering Key Features in Protein Structures with The New ENDscript Server. *Nucleic Acids Res* **2014,** *42* (Web Server issue), W320-4.
12. Vijayasarathy, S.; Prasad, P.; Fremlin, L. J.; Ratnayake, R.; Salim, A. A.; Khalil, Z.; Capon, R. J., C3 and 2D C3 Marfey's Methods for Amino Acid Analysis in Natural Products. *Journal of Natural Products* **2016,** *79* (2), 421-427.
13. Luo, Y.; Xu, S.; Frerk, A. M.; van der Donk, W. A., Facile Method for Determining Lanthipeptide Stereochemistry. *Analytical Chemistry* **2024,** *96* (4), 1767-1773.
14. Liu, X.; Xing, Y.; Yuen, M.; Yuen, T.; Yuen, H.; Peng, Q., Anti-Aging Effect and Mechanism of Proanthocyanidins Extracted from Sea buckthorn on Hydrogen Peroxide-Induced Aging Human Skin Fibroblasts. *Antioxidants* **2022,** *11* (10), 1900.
15. Pan, C.; Lang, H.; Zhang, T.; Wang, R.; Lin, X.; Shi, P.; Zhao, F.; Pang, X., Conditioned Medium Derived from Human Amniotic Stem Cells Delays H_2O_2-Induced Premature Senescence in Human Dermal Fibroblasts. *Int J Mol Med* **2019,** *44* (5), 1629-1640.
16. Schubert, M.; Labudde, D.; Oschkinat, H.; Schmieder, P., A Software Tool for The Prediction of Xaa-Pro Peptide Bond Conformations in Proteins Based on ^{13}C Chemical Shift Statistics. *Journal of Biomolecular NMR* **2002,** *24* (2), 149-154.
17. Lu, J.; Li, J.; Wu, Y.; Fang, X.; Zhu, J.; Wang, H., Characterization of the FMN-Dependent Cysteine Decarboxylase from Thioviridamide Biosynthesis. *Organic Letters* **2019,** *21* (12), 4676-4679.
18. Zhao, C.; Sheng, W.; Wang, Y.; Zheng, J.; Xie, X.; Liang, Y.; Wei, W.; Bao, R.; Wang, H., Conformational Remodeling Enhances Activity of Lanthipeptide Zinc-Metallopeptidases. *Nature Chemical Biology* **2022,** *18* (7), 724-732.
19. Mukherjee, S.; van der Donk, W. A., Mechanistic Studies on the Substrate-Tolerant Lanthipeptide Synthetase ProcM. *Journal of the American Chemical Society* **2014,** *136* (29), 10450-10459.
20. Qiu, Y.; Liu, J.; Li, Y.; Xue, Y.; Liu, W., Formation of an Aminovinyl-Cysteine Residue in Thioviridamides Occurs through a Path Independent of Known Lanthionine Synthetase Activity. *Cell Chemical Biology* **2021,** *28* (5), 675-685.e5.
21. Huang, S.; Wang, Y.; Cai, C.; Xiao, X.; Liu, S.; Ma, Y.; Xie, X.; Liang, Y.; Chen, H.; Zhu, J.; Hegemann, J. D.; Yao, H.; Wei, W.; Wang, H., Discovery of a Unique Structural Motif in Lanthipeptide Synthetases for Substrate Binding and Interdomain Interactions. *Angewandte Chemie International Edition* **2022,** *61* (45), e202211382.
22. Lu, J.; Wu, Y.; Li, Y.; Wang, H., The Utilization of Lanthipeptide Synthetases Is a General Strategy for the Biosynthesis of 2-Aminovinyl-Cysteine Motifs in Thioamitides. *Angewandte Chemie International Edition* **2021,** *60* (4), 1951-1958.
23. Ortiz-Lombardía, M.; Pompeo, F.; Boitel, B.; Alzari, P. M., Crystal Structure of the Catalytic Domain of the PknB Serine/Threonine Kinase from *Mycobacterium tuberculosis*. *Journal of Biological Chemistry* **2003,** *278* (15), 13094-13100.
24. Scherr, N.; Honnappa, S.; Kunz, G.; Mueller, P.; Jayachandran, R.; Winkler, F.; Pieters, J.; Steinmetz, M. O., Structural Basis for The Specific Inhibition of Protein Kinase G, A Virulence Factor of *Mycobacterium tuberculosis*. *Proceedings of the National Academy of Sciences* **2007,** *104* (29), 12151-12156.
