# Supplementary material for: A Unique Class of Cyclases with a Kinase Fold Catalyzes Enethiol-Mediated Macrocyclization of Aminovinyl-Cysteine Motifs in Lanthipeptides
Source: ACS Cent Sci. 2025 Jun 18;11(7):1178–88. doi: 10.1021/acscentsci.5c00569 (PMC12291118; doi:10.1021/acscentsci.5c00569)
Supplement: Supplementary file 2 [file oc5c00569_si_002.pdf]

oc-2025-00569x.R1

Name: Peer Review Information for "A Unique Class of Cyclases with a Kinase Fold Catalyzes Enethiol-mediated Macrocyclization of Aminovinyl-Cysteine Motifs in Lanthipeptides"

First Round of Reviewer Comments

Reviewer: 1

Comments to the Author

In this manuscript, the authors discovered that the regio- and stereoselective AviMeCys macrocyclization is catalyzed by RosX, a newly identified cyclase with a kinase-like fold. Additionally, the authors demonstrate that Lan formation in rosins proceeds via a substrate-controlled cyclization pathway mediated by the kinase RosK and lyase RosY complex. I recommend acceptance after some revision.

1. The authors use AviMeCys and Avi(Me)Cys. Are they different? If they are identical, please use the unified name.

2. I wonder why the geometry of enthiol (RosD product) is trans after decarboxylation despite that the final AviMeCys moiety has the cis double bond. How does the geometry flip?

3. “Results show that the AviMeCys macrocycles derived from RosA1Lan-AviMeCys and rosin A1 have the same retention time, suggesting that they possess the same structure” The authors compared the retention time; however, the same retention time does not necessarily mean the same compound. Diastereomers may coelute. Please give more solid evidence.

4. Is RosY a monomer without RosK?

5. In the main text, the authors proposed that the cyclization is nonenzymatic; however, the figure 3d suggests that the cyclization is RosKY-mediated (green box). Please fix this.

6. RosX binds to RosA1LP with a KD of (6 ± 2) μM (Fig. 5b, Fig. S40) and primarily through

7. its N-terminal segment (Fig. S40). Please briefly explain how the authors determined these.

8. "but lacks putative ATP and Mg2+ binding motifs conserved in kinases" Please provide more detailed information about how RosX is different form typical kinases.

9. The authors use AlphaFold2 and AlphaFold3. Is there any reason why the authors used two different versions of AlphaFold?

10. The authors mentioned that Glu220, His269 and Asp272 are highly conserved among LanX proteins. Are these also conserved in typical kinases? Do mutations of these residues actually have impact on the catalytic activity of RosX?

11. The authors showed that RosX is critical for regioselectivity of the cyclization. Do the authors have evidence that RosX accelerate the cyclization reaction compared to the non-enzymatic

12. The difference between the two RosX sequences shown in Figure S44 should be clarified.

13. The timing of cysteine decarboxylation and AviMeCys macrocyclization should be further investigated.

14. If possible, the binding affinity of RosX to the RosK-RosY reaction product (without AviCys) should be measured.

15. A more detailed comparison between RosX and TvaE should be included and discussed in the manuscript.

Reviewer: 2

Comments to the Author

This manuscript describes a class of cyclases catalyzing enethiol-mediated macrocyclization of aminovinyl-cysteine motifs in lanthipeptides by discovery, structural elucidation, and biosynthesis characterization of class V lanthipeptides, rosins. The study provides a thorough in vitro reconstitution of each gene in the ros BGC, demonstrating their functionality. Furthermore, the work seems to be promising potential for future

applications in enzymatic studies. The authors' conclusions are well supported by experimental evidence, and this research represents a significant contribution to the field. Therefore, the reviewer strongly recommends that this manuscript should be an excellent contribution to Journal of the American Chemical Society after minor revision.

Please improve the manuscript by considering the following points.

1. The identification of the ros BGC was achieved through genome mining. However, the manuscript does not describe the genome mining methodology. Please include details regarding the genome mining procedure, including tools, databases, and selection criteria.

2. As NMR is a reliable analytical method for structure elucidation, it is essential to provide complete assignments—or as complete as possible—for all amino acid residues in Table S2. However, several residues are listed without NMR assignments, such as ketobutyrate-1 and Dbu-2. Furthermore, the NMR data for Dbu-7 is incomplete: only the Hγ chemical shift is reported, while the corresponding 13C shift is missing. A thorough report of the NMR table is therefore necessary.

3. The geometry configuration of Dhb residues should be clearly assigned. Cis/trans conformer assignment of Proline residues is also important. All this information will assist 3D structure modeling and also serve as an important reference for future studies.

4. Marfey's method: Both derivatization with FDAA of L- and D- standard amino acids together with hydrolysate should be reported to prove that the used LCMS condition can differentiate derivatives. Note that tyrosine can form either mono- or bis-FDAA derivatives, it is recommended to report bis-FDAA derivatives for clarity and consistency.

5. Page 4, line 203: The statement "a Z-geometry of the double bond in the AviMeCys crosslink was determined based on the corresponding 3JH,H value of 7.2 Hz" appears to conflict with the NOE correlations shown in Figure 2c. If Z-configuration is indeed correct, NOE signals such as those between both NH and Hα of TEE-25 and Hβ of the Abu moiety may be weak or insignificant. Please verify these NOE correlations and indicate whether they are significant.

6. Page 5, line 215-227: The current description of the AviMeCys configuration assignment is difficult to follow. To enhance clarity, consider using Newman projections or alternative schematic representations.

Typos are found in the manuscript.

1. Figure 1b, Dha and Dhb should be labeled consistently with their corresponding substructures. Please revise to ensure correct correspondence.

2. Abbreviations, such as “Abu,” are used without being defined at first mention. Please ensure all abbreviations are introduced in full upon first use and consistently applied throughout the manuscript.

Reviewer: 3

Comments to the Author

In this study Xie et al describe the first in vitro demonstration that a gene annotated originally as a kinase-like protein is involved in the formation of aminovinyl cysteine rings in class Va lanthipeptides. Previous studies by Deng and coworkers had implicated this protein as important based on its need for lexapeptide formation but without directly showing its activity. Another previous study by Liu et al had shown the same for thioviridamide formation and had assigned the function of the kinase-like enzyme as catalyzing the AviCys cyclization together with a decaboxylase (ref 41). The current study verifies these conclusions with in vitro activity and complements those data with several other pieces of data. The study is generally well supported by the data shown in the SI, with some concerns discussed below. If the authors can address these concerns, this study could be appropriate for publication in ACS Cent Sci.

In the abstract (and elsewhere), the wording “their biosynthetic machinery, particularly the cyclases catalyzing Avi(Me)Cys macrocyclization, has remained unknown” is not really correctly describing what is known. Ref 41, which is cited very late in the current paper, provided strong support that the RosX ortholog was directly involved in AviCys formation. In fact, the conclusions in ref 41 closely parallel the conclusions in the current study, with the important distinction that the current study shows direct in vitro evidence whereas ref 41 used co-expression. Hence, I believe that the authors of the current study should more appropriately acknowledge the findings in ref 41 and do so earlier. Yes, that study focused on thioviridamides and not lanthipeptides, but the exact same set of genes are involved. The current presentation of discovery of a new activity is not really describing accurately what was known.

The current work shows that the first ring in rosin A1 can be formed non-enzymatically with some facilitation by RosYK. But the authors do not at all comment on their conclusion that this spontaneously formed ring has the "DL"-configuration. They cite several other studies where spontaneous cyclization from a similar motif has been reported, but as far as I can tell, those other studies all generated LL stereochemistry. Hence, it seems that the system under study is an exception but the authors do not seem to recognize this or do not want to point out the difference. Please discuss.

The authors report they used advanced Marfey analysis using 1-fluoro-2,4-dinitrophenyl-5-L/D-alanine amide (L/D-FDAA) which is a confusing way of wording. Researchers usually use either L-FDAA or D-FDAA, not a mixture. In fact in Fig S7 it seems they only used L-FDAA. The other confusing thing is that advanced Marfey analysis refers to the use of the Leu derivative (L-FDLA), not FDAA. The authors seem to use the regular Marfey's method and not the advanced method.

On the topic of stereochemistry, the authors always draw the enethiol as having E stereochemistry in several figures. What is the support for this? If indeed E, how is the stereochemistry changed to Z in the cyclized product?

One of the more unique aspects of this report is the demonstration of promotion of wound healing. Since this is highly likely to be a non-physiological activity, the authors should test if the unmodified core peptide (or any of the analogs they made) has the same activity or whether the modifications are required.

The authors mention that their trimer of RosK and RosY is different from other studies on homologous proteins. They should make an AlphaFold3 model of this trimer and compare its pLDDT values with the dimer. Also, the authors mention that RosK forms a dimer but they do not say whether RosY by itself is a monomer or dimer. I am not convinced that SEC data with just one standard (SpaC-SpaD) and without a standard curve with a series of standards is sufficient support for the proposed 2:1 stoichiometry. AlphaFold and a standard curve may be able to provide stronger support.

Regarding using AlphaFold, what is the predicted structure of RosA1 binding to RosY-RosK? While the prediction of alpha helicity for the substrate by itself looks well supported, the peptide could bind very differently to the enzyme.

All of the following statements are missing citations or the citations are incomplete:

“Unlike other reported class V lanthipeptide BGCs, the ros cluster does not contain a methyltransferase.”

“This is distinct from their homologs CaoK-CaoY from cacaoidin biosynthesis and SpaC-SpaD from thiosparsoamide biosynthesis, which form heterodimer in solution.”

“MSMS analysis also revealed the presence of an N-terminal 2-oxobutyryl group (Obu), which is likely generated from the spontaneous deamination of an N-terminal Dhb residue after the leader removal (Fig. S6b).23” This was first shown in Kellner, R.; Jung, G.; Josten, M.; Kaletta, C.; Entian, K. D.; Sahl, H. G. Angew. Chem. 1989, 101, 618 and should be cited.

“LanX has been proposed to be essential for the Avi(Me)Cys biosynthesis in class Va lanthipeptide and thioamitides.”

“Consistent with typical LanD proteins, RosD forms a dodecamer in vitro, as determined by SEC analysis (Fig. S33a).33” While ref 33 by the authors indeed also shows this, this observation was already made much earlier in EMBO J . 2000 Dec 1;19(23):6299-310.

I did not understand the citation of ref 29 in this sentence: “Although the Ala6-Cys10 Lan crosslink (Lan6-10) was identical to that in the authentic rosin A1, the C-terminal Ala15-Cys25 Lan crosslink was formed with distinct regioselectivity compared with the AviMeCys crosslink in rosin A1.29" I read ref 29 and did not understand its relevance to this sentence. Please expand text.

Fig. S22, panel c. This is an odd-looking maximum likelihood phylogenetic plot. What are the distances/bootstrap values? How was it generated? I could not find it in the Methods or legend.

Typo’s:

Figure legend Fig 1 has text repeated (bold text)

Page 3: 87 sturctures:

Bacillus subtills

Page 5, line 280 There results should be These results.

Fig 3 legend ration should be ratio

Author's Response to Peer Review Comments:

Point-by-point Response

Formatting Needs:

1. Abstract: Please make sure the word count of your Abstract does not exceed 200 words.

   Response to the comment:

   Thanks for the careful evaluation, we have edited the abstract to 191 words.

2. Author List: Author list must match exactly in three places: (1) manuscript file, (2) supporting information, and (3) ACS Paragon Plus. Jiang-Tao Gao is not listed in the SI file.

   Response to the comment:

   Thanks for the careful evaluation. We have updated the full author in revised Supporting Information, please see **Page S1**.

3. Supporting Information: Please number all pages in the following format: S1, S2, S3, etc.

   Response to the comment:

   Thanks for pointing this out. We have updated the Supporting Information pagination to "S1, S2, S3, …" as suggested.

4. Synopsis: ACS Central Science requires a brief synopsis. The synopsis should be no more than 200 characters (including spaces) and should reasonably correlate with the Table of Contents (TOC) graphic. The synopsis is intended to explain the importance of the article to a broader readership across the sciences. Please place your synopsis in the manuscript file after the TOC graphic and label as "Synopsis."

   Response to the comment:

   We have added a brief synopsis after the TOC graphic in the revised manuscript, please see **Page 13**.

5. TOC Graphic: Include a TOC graphic illustrating the significance of the paper. The TOC graphic should be something that is representative of your entire work. Color schemes or illustrations typically make good choices. The TOC graphic must be original and free from any copyright issues. Confirm that all text is legible. Present the TOC graphic on the last page of the manuscript by itself. Please label the TOC as "TOC Graphic". A caption describing the TOC is not needed. Please see more information/guidelines for TOC Graphics at the following link: http://pubsapp.acs.org/paragonplus/submission/toc_abstract_graphics_guidelines.pdf?

   Response to the comment:

   We have added a TOC graphic in the revised manuscript, please see **Page 13**.

Reviewer: 1

Recommendation: Publish in ACS Central Science after minor revisions noted.

Comments:

In this manuscript, the authors discovered that the regio- and stereoselective AviMeCys macrocyclization is catalyzed by RosX, a newly identified cyclase with a kinase-like fold. Additionally, the authors demonstrate that Lan formation in rosins proceeds via a substrate-controlled cyclization pathway mediated by the kinase RosK and lyase RosY complex. I recommend acceptance after some revision.

1. The authors use AviMeCys and Avi(Me)Cys. Are they different? If they are identical, please use the unified name.

Response to the comment:

Thanks for the comment.

We have provided the following description to explicitly distinguish between the terminologies of AviMeCys and Avi(Me)Cys in revised manuscript:

"(2*S*, 3*S*)-*S*-((*Z*)-2-aminovinyl)-D-cysteine (AviCys) or (2*S*, 3*S*)-*S*-((*Z*)-2-aminovinyl)-3-methyl-D-cysteine (AviMeCys), collectively referred to as Avi(Me)Cys motifs"

This nomenclature follows a review article on AviCys-containing natural products: Botao Cheng et al., *ChemPlusChem* **2024**, e202400047.

Please see **Page 2, Line 32-35** in the revised manuscript for details.

2. I wonder why the geometry of enethiol (RosD product) is trans after decarboxylation despite that the final AviMeCys moiety has the cis double bond. How does the geometry flip?

Response to the comment:

Thanks for this question.

The geometry of enethiol (RosD product) is generally proposed to adopt a cis-configuration after Cys decarboxylation (Clarissa S. Sit et al., *Acc. Chem. Res.* **2011**, 44, 261-268; Emily S. Grant-Mackie et al., *JACS Au* **2021**, 1, 1527-1540; Botao Cheng et al., *ChemPlusChem* **2024**, e202400047). Theoretical analysis of Avi(Me)Cys derivatives supports that the cis-configuration is thermodynamically favorable (Angela K. Carrillo et al., *Org. Lett.* **2017**, 19, 5146-5149).

In the original manuscript, we made mistakes by drawing the enethiol motif in *trans* configuration in Fig. 4a and Fig. S33. We have corrected these mistakes accordingly.

Please see the revised manuscript **Page 8, Figure 4a**, and the revised Supporting Information **Page S55, Figure S33** for details.

3. “Results show that the AviMeCys macrocycles derived from RosA1$_{Lan\text{-}AviMeCys}$ and rosin A1 have the same retention time, suggesting that they possess the same structure” The authors compared the retention time; however, the same retention time does not necessarily mean the same compound. Diastereomers may coelute. Please give more solid evidence.

Response to the comment:

We thank the reviewer for this comment.

To further support the conclusion that the AviMeCys macrocycles derived from RosA1$_{Lan\text{-}AviMeCys}$ and native rosin A1 share identical stereochemistry, we conducted Marfey’s analysis on two samples: (1) the AviMeCys macrocycle from RosA1$_{Lan\text{-}AviMeCys}$ (designated AviMeCys-1), and (2) the AviMeCys macrocycle from rosin A1(referred to as authentic AviMeCys). Since the Abu motif represents the sole stereocenter generated during macrocyclization in both compounds, we focused on its analysis. Results showed that the Abu residues in AviMeCys-1 and authentic AviMeCys exist as single isomers exclusively in the D-configuration (Fig. R1). This result further support that the enzymatically synthesized AviMeCys macrocycle in RosA1$_{Lan\text{-}AviMeCys}$ is structurally identical to that in the naturally derived rosin A1, confirming the fidelity of the *in vitro* enzymatic modifications.

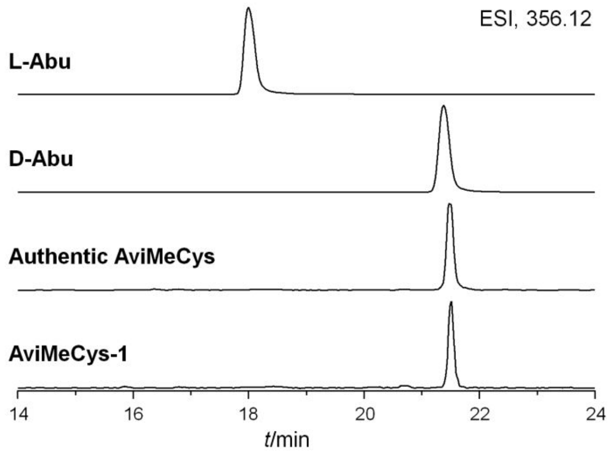

Fig. R1 Marfey’s analysis of the Abu residue derived from the hydrolysis of the AviMeCys motif in RosA1$_{Lan\text{-}AviMeCys}$ (designated AviMeCys-1), with the authentic AviMeCys motif from rosin A1 as a control.

Please see the revised manuscript **Page 7, Line 468-479** and the revised Supporting Information **Page S59, Figure S37** for details.

4. Is RosY a monomer without RosK?

Response to the comment:

Thanks for the question.

We performed a systematic analysis of protein oligomerization status using size-exclusion chromatography (SEC) data (Fig. 3a and Fig. S22), RosY exists as a monomer without RosK in

aqueous solution. We have added one sentence in the revised manuscript to further clarify this point.

Please see the revised manuscript (**Page 5, Line 298**; **Page 6, Figure 3a**) and the revised Supporting Information (**Page S37, Figure S22a**) for details.

5. In the main text, the authors proposed that the cyclization is nonenzymatic; however, the figure 3d suggests that the cyclization is RosKY-mediated (green box). Please fix this.

Response to the comment:

We thank the reviewer for identifying this inconsistency, we have corrected Fig. 3d in the revised manuscript.

Please see the revised manuscript **Page 6, Figure 3d**.

6. RosX binds to RosA1$_{LP}$ with a K_D of (6 ± 2) μM (Fig. 5b, Fig. S40) and primarily through its N-terminal segment (Fig. S40). Please briefly explain how the authors determined these.

Response to the comment:

Thanks for the comment.

We agree with the reviewer that the original description was not accurate and have revised the original text as following:

"RosX binds to RosA1$_{LP}$ with a K_D of (6 ± 2) μM (Fig. 5b, Fig. S40). Truncation of the predicted α-helix spanning residues -18 to -26 significantly decreased the RosX-RosA1$_{LP}$ binding (Fig. S40), which is similar to that observed in the RosA1$_{LP}$ recognition by RosK-RosY (Fig. 3c)."

Please see the revised manuscript **Page 8, Line 497-502**.

7. "But lacks putative ATP and Mg^{2+} binding motifs conserved in kinases" Please provide more detailed information about how RosX is different form typical kinases.

Response to the comment:

Thanks for the comment.

We have performed comprehensive sequence alignments of RosX with representative kinases including protein kinases PknB and PknG, as well as lanthipeptide kinases TvaC, CurKC, SpaKC (Fig. R2 and Fig. S44d in the revised SI). These analyses consistently demonstrate that RosX lacks the conserved residues critical for Mg^{2+} coordination and catalytic activity that are characteristic of typical kinases.

We have provided the following description in revised manuscript to delineate the differences between RosX and typical kinases:

"But RosX lacks putative catalytic and Mg^{2+} binding residues conserved in typical kinases. Specifically, key residues present in LxmK, such as those involved in catalysis (Asp221), Mg^{2+}

coordination (Asp241 and Gln226), and ATP binding (Arg49 and Lys66), are completely absent in RosX (Fig. S44cd)."

Please see the revised manuscript **Page 9, Line 542-548**, and the revised Supporting Information **Page S66, Figure S44d** for details.

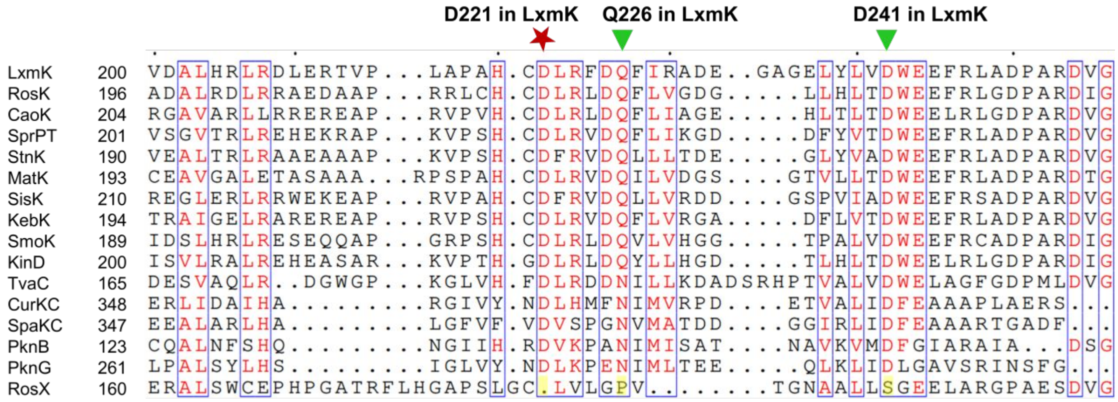

Fig. R2 Sequence alignment of RosX with representative kinases (TvaC, CurKC, SpaKC, PknB, and PknG) demonstrating the absence of conserved Mg^{2+}-coordinating and catalytic residues in RosX. The conserved catalytic residue Asp is indicated by red star. Mg^{2+}-chelating residues Asn and Asp are highlighted with green triangles.

8. The authors use AlphaFold2 and AlphaFold3. Is there any reason why the authors used two different versions of AlphaFold?

Response to the comment:

Thanks for the question.

In the initial stages of our study, AlphaFold3 had not yet been published, which necessitated our use of AlphaFold2 for structural predictions.

Following the reviewer's request, we have now systematically updated all structural predictions in the revised manuscript to the AlphaFold3 versions, including Fig. 3c and Fig. S44

Please see the revised manuscript **Page 6, Fig. 3c**, and the revised Supporting Information **Page S66, Figure S44ab** for details.

9. The authors mentioned that Glu220, His269 and Asp272 are highly conserved among LanX proteins. Are these also conserved in typical kinases? Do mutations of these residues actually have impact on the catalytic activity of RosX?

Response to the comment:

Thanks for the comments.

Residues Glu220, His269 and Asp272 in RosX are highly conserved among LanX proteins (Fig. S45d), and Ala mutations of these residues abolished the capability of RosX to catalyze the

correct AviMeCys macrocyclization in RosA1 peptide (Fig. S46). In contrast, typical kinases display low sequence similarity with RosX in related regions, and Glu220/ His269/Asp272 have no conserved counterpart residues in kinases. This analysis is demonstrated in Fig. R3 and the newly added Fig. S45e in the revised SI.

Please see the revised manuscript **Page 9, Line 558-559**, and the revised Supporting Information **Page S67, Figure S45e** for details.

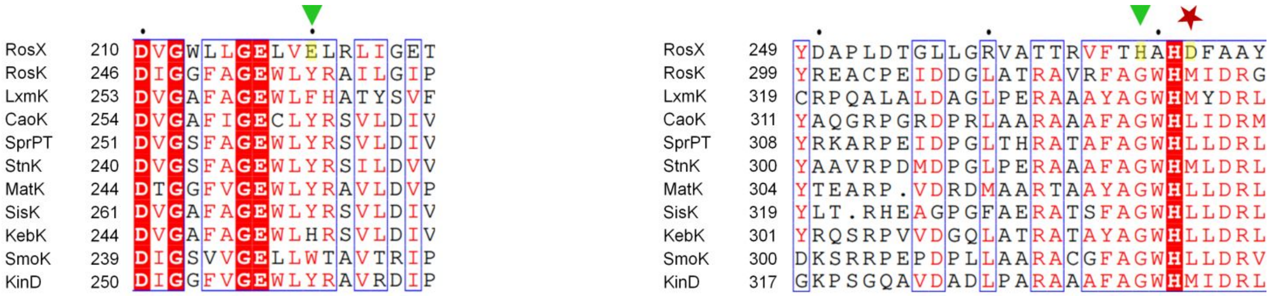

Fig. R3 Sequence alignment analysis demonstrated that LanKs lack the conserved Glu220, His269, and Asp272 residues present in RosX.

10. The authors showed that RosX is critical for regioselectivity of the cyclization. Do the authors have evidence that RosX accelerate the cyclization reaction compared to the non-enzymatic?

Response to the comment:

We appreciate the reviewer's insightful inquiry.

As described in our original manuscript, our experimental data demonstrate that RosX governs the regioselectivity of AviMeCys macrocyclization. In the absence of RosX, the native cyclization pathway is disrupted, leading to predominant accumulation of the RosA1$_{\text{Lan-AviCys (15–25)}}$ byproduct alongside additional minor derivatives (Fig. R4). These findings also suggest that the Michael addition between the enethiol motif and a Dha residue proceeds rapidly under aqueous conditions as a competing side reaction.

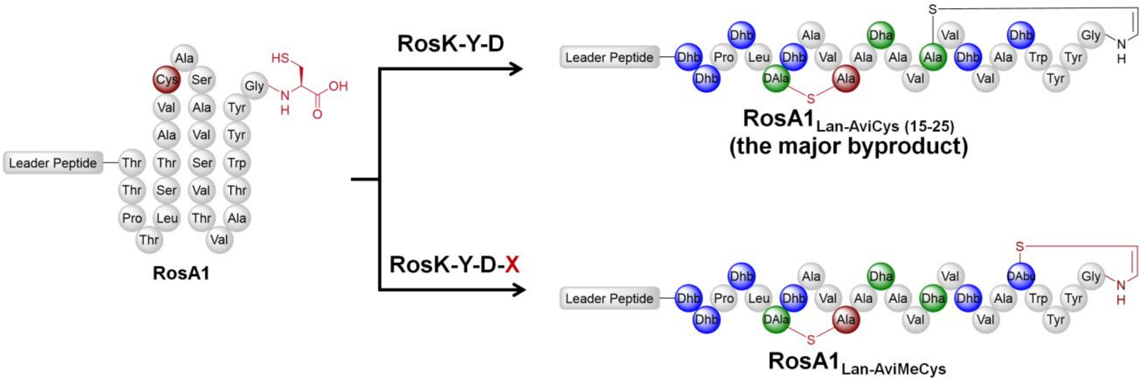

Fig. R4 The modifications of RosA1 by RosK-Y-D and RosK-Y-D-X.

To directly address the reviewer's question regarding the kinetic contribution of RosX to AviMeCys cyclization, we engineered two RosA variants (RosA1$_{S15A}$ and RosA1$_{S12A_S15A}$)

designed to eliminate the enethiol-Dha side reaction. This strategy aimed to isolate and compare the kinetics of enzymatic (RosX-dependent) versus non-enzymatic cyclization pathways. However, we found that RosK-Y-D catalysis of RosA1$_{S15A}$ unexpectedly generated an alternative AviCys ring (12-25 crosslink) (Fig. R5). Furthermore, we were not able to express RosA1$_{S12A_S15A}$ in *E. coli*, probably due to its degradation after expression. Regrettably, these technical challenges prevented the generation of a suitable peptide substrate to quantitatively dissect RosX's catalytic kinetics.

Moving forward, we plan to investigate analogous class V lanthipeptide biosynthetic systems, which may provide alternative strategies to resolve this mechanistic question. We remain committed to elucidating the kinetic role of RosX and anticipate reporting progress in future studies.

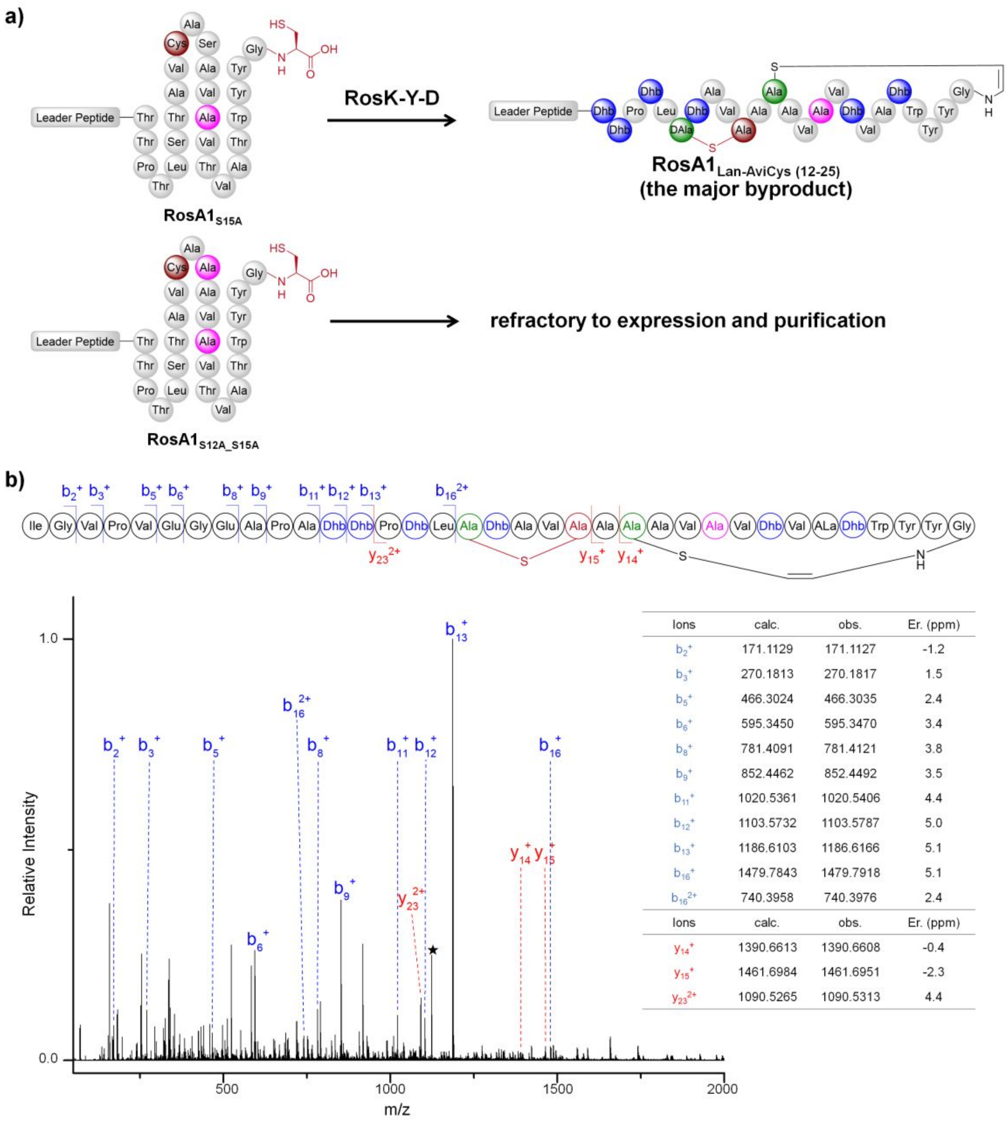

Fig. R5 (a). RosK-Y-D catalyzes the conversion of $RosA1_{S15A}$ into $RosA1_{Lan\text{-}AviCys(12–25)}$, the double mutant $RosA1_{S12A\_S15A}$ was difficult to obtain; (b). MS/MS analysis of $RosA1_{Lan\text{-}AviCys(12–25)}$. The *b* and *y* ions are listed in table and marked in the spectrum. The asterisk indicates $[M+3H]^{3+}$.

11. The difference between the two RosX sequences shown in Figure S44 should be clarified.

Response to the comment:

We appreciate the reviewer's detailed attention to comparative sequence analysis of RosX with typical kinases (LanKs) from class V lanthipeptide biosynthesis in Fig. S44cd.

The upper and lower panels represent distinct regions of sequence alignment between RosX and LanKs. Numeric labels along the left margin indicate the position of the first amino acid in the corresponding protein sequence. The upper panel spans residues 19 to 57 in the RosX sequence, while the lower panel covers residues 160 to 202. We have added a two-sentence description in the legend of Fig. S44 and Fig. S45.

Please see the revised Supporting Information **Page S66, Line 536-537** and **Page S67, Line 547-548** for details.

12. The timing of cysteine decarboxylation and AviMeCys macrocyclization should be further investigated.

Response to the comment:

We appreciate the reviewer's insightful suggestion.

Our findings support a biosynthetic sequence in which RosD-catalyzed decarboxylation of Cys25 precedes both the RosK-RosY-mediated dehydration of the RosA1 precursor and subsequent RosX-dependent cyclization. This conclusion is anchored in the observation that Cys25-to-Dha15 lanthionine crosslinking as a side reaction occurs rapidly following peptide dehydration (Fig. R6). Once this crosslink forms, the opportunity for decarboxylation at Cys25 is irreversibly blocked, as the modified residue is no longer accessible to RosD.

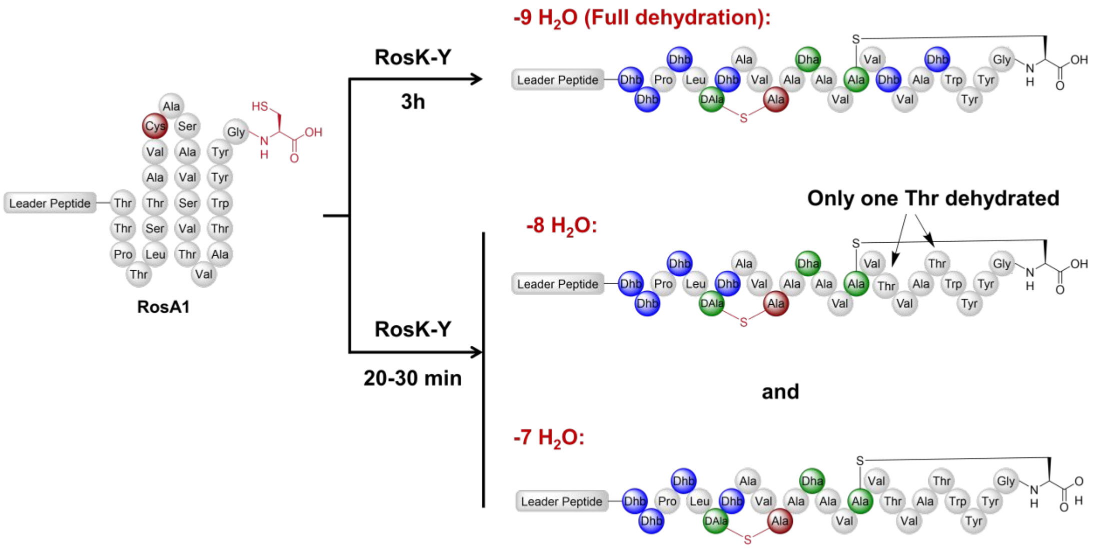

Fig. R6 Products of RosA1 derived from full and partial modification by RosK-RosY.

The reviewer’s insightful comment promotes us to further evaluate an alternative biosynthetic sequence: (1) the RosK-RosY-catalyzed dehydration of Thr17 and Thr20 residues, (2) the RosD-catalyzed Cys decarboxylation, (3) the RosX-catalyzed AviMeCys cyclization, and (4) RosK-RosY continue to dehydrate the rest of Ser/Thr residues N-terminally to the AviMeCys ring.

To test this hypothesis, we performed time-course assays of RosK-RosY activity. At abbreviated reaction times (20–30 min), we detected intermediates with only 7–8 dehydrations (vs. the full complement of 9). LC-MS/MS analysis revealed that Thr17 and Thr20 were the last dehydration sites, and the Cys25-to-Dha15 lanthionine crosslinking was already evident in these partially dehydrated intermediates (Fig. R6). These results further support that decarboxylation should occur prior to both dehydration and cyclization.

We have provided addition description of these results in the revised manuscript. Please

see the revised manuscript **Page 6, Line 365-372**, and the revised Supporting Information **Page S43-44, Figure S27c-d** for details. We again thank the reviewer for prompting this deeper mechanistic inquiry, which has further solidified our proposed biosynthetic sequence.

13. If possible, the binding affinity of RosX to the RosK-RosY reaction product (without AviCys) should be measured.

Response to the comment:

Thanks for the question.

We prepared and accumulated RosK-RosY-modified RosA1 of enough quantity and measured its binding with RosX. Results showed that RosK-RosY-modified RosA1 exhibited a binding affinity of $K_D \geq 5 \pm 2$ μM, which is comparable to that of the RosA1 leader peptide (K_D = 6 ± 2 μM) (Fig. R7). This result indicates that RosX primarily binds to the leader peptide of modified RosA peptide.

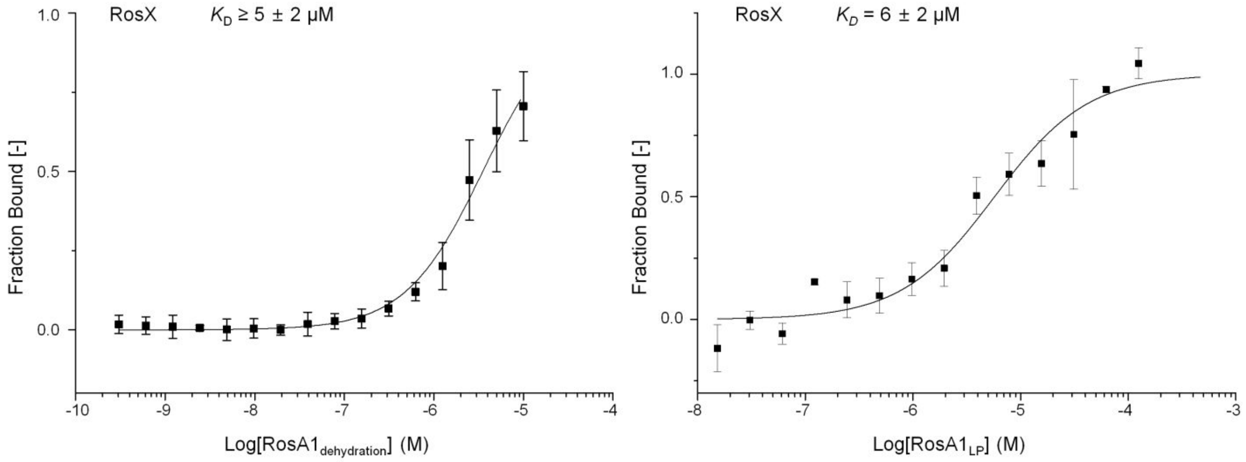

Fig. R7 Binding affinities between RosX and RosK-RosY-modified RosA1, RosX and RosA1$_{LP}$ measured by MST. Data represent the mean ± s.d. from three replicates.

14. A more detailed comparison between RosX and TvaE should be included and discussed in the manuscript.

Response to the comment:

We sincerely appreciate the reviewer's suggestion to further compare RosX and TvaE$_{S\text{-}87}$, both of which catalyze Avi(Me)Cys macrocyclization. We have now added detailed sequence and structural comparisons in the revised *DISCUSSION* section：

"Comparative analysis reveals that RosX shares limited sequence similarity with TvaE$_{S\text{-}87}$ (~23%), and phylogenetic analysis places them in distinct clades. RosX and TvaE$_{S\text{-}87}$ display overall structural similarity in their predicted structures with a kinase-like fold (RMSD = 3.8 Å). Both RosX and TvaE$_{S\text{-}87}$ lack the canonical catalytic residues and Mg^{2+}-chelating motif typical of kinases."

Please see the revised manuscript **Page 9, Line 636-644**, and the revised Supporting Information **Page S74, Figure S52** for details.

Reviewer: 2

Recommendation: Publish in ACS Central Science after minor revisions noted.

Comments:

This manuscript describes a class of cyclases catalyzing enethiol-mediated macrocyclization of aminovinyl-cysteine motifs in lanthipeptides by discovery, structural elucidation, and biosynthesis characterization of class V lanthipeptides, rosins. The study provides a thorough in vitro reconstitution of each gene in the *ros* BGC, demonstrating their functionality. Furthermore, the work seems to be promising potential for future applications in enzymatic studies. The authors' conclusions are well supported by experimental evidence, and this research represents a significant contribution to the field. Therefore, the reviewer strongly recommends that this manuscript should be an excellent contribution to Journal of the American Chemical Society after minor revision.

Please improve the manuscript by considering the following points.

1. The identification of the *ros* BGC was achieved through genome mining. However, the manuscript does not describe the genome mining methodology. Please include details regarding the genome mining procedure, including tools, databases, and selection criteria.

Response to the comment:

We sincerely appreciate the reviewer's suggestion.

We have now provided a detailed description of the genome mining process in both the main text and the Methods section in the revised Supporting Information. Please see the revised manuscript **Page 3, Line 140-152**, and Supporting Information **Page S3, Line 52-65**.

The final set of 151 target genomic accessions and their corresponding strain names have been compiled in **Page S10-11, Table S1** of the revised Supporting Information.

2. As NMR is a reliable analytical method for structure elucidation, it is essential to provide complete assignments—or as complete as possible—for all amino acid residues in Table S2. However, several residues are listed without NMR assignments, such as ketobutyrate-1 and Dbu-2. Furthermore, the NMR data for Dbu-7 is incomplete: only the Hγ chemical shift is reported, while the corresponding 13C shift is missing. A thorough report of the NMR table is therefore necessary.

Response to the comment:

Thank you for pointing these out.

We have made every effort to assign nearly all the chemical shifts. The assignments for ketobutyrate-1 and Dbu-2 are now nearly complete. We have corrected the typos in the Leu-5 δ atom assignments and added the missing γ atom assignments.

Please refer to **Page S13, Table S3** and **Page S27, Figure S13** in the revised SI for details.

3. The geometry configuration of Dhb residues should be clearly assigned. Cis/trans conformer assignment of Proline residues is also important. All this information will assist 3D structure modeling and also serve as an important reference for future studies.

Response to the comment:

Thank you for the careful evaluation.

Indeed, the geometric configurations of Dhb and Pro residues are critical for accurate 3D structure modeling and serve as important references for future studies. In our work, the geometry of Dhb residues was primarily determined by comparing the intensities of NOE cross peaks between NH–Hβ and NH–Hγ. The NH–Hγ cross peak was significantly stronger than the NH–Hβ cross peak (Figure S12), indicating a shorter distance between NH and Hγ. For proline residues, the cis/trans conformation of Xxx–Pro peptide bonds were identified based on the chemical shift difference between the Cβ and Cγ atoms [Δδ(Cβ–Cγ)], with reported values of 4.5 ± 1.2 ppm for trans and 9.6 ± 1.3 ppm for cis conformations (Mario Schubert et al., *J. Biomol.* NMR **2002**, 24:149–154). In the case of rosin A1, Pro-3 exhibits a Δδ(Cβ–Cγ) of 4.6 ppm, indicating a trans conformation.

Please see the revised Supporting Information **Page S13, Line 254** and **Page S26, Line 311-312** for details.

4. Marfey's method: Both derivatization with FDAA of L- and D- standard amino acids together with hydrolysate should be reported to prove that the used LCMS condition can differentiate derivatives. Note that tyrosine can form either mono- or bis-FDAA derivatives, it is recommended to report bis-FDAA derivatives for clarity and consistency.

Response to the comment:

We thank the reviewer for these valuable suggestions to improve the rigor of Marfey's analysis. In response, we have now performed comprehensive derivatization experiments with both L- and D-standard amino acids under identical LC-MS conditions. For consistency, we have modified all tyrosine analyses to exclusively report bis-FDAA derivatives (Fig. R8).

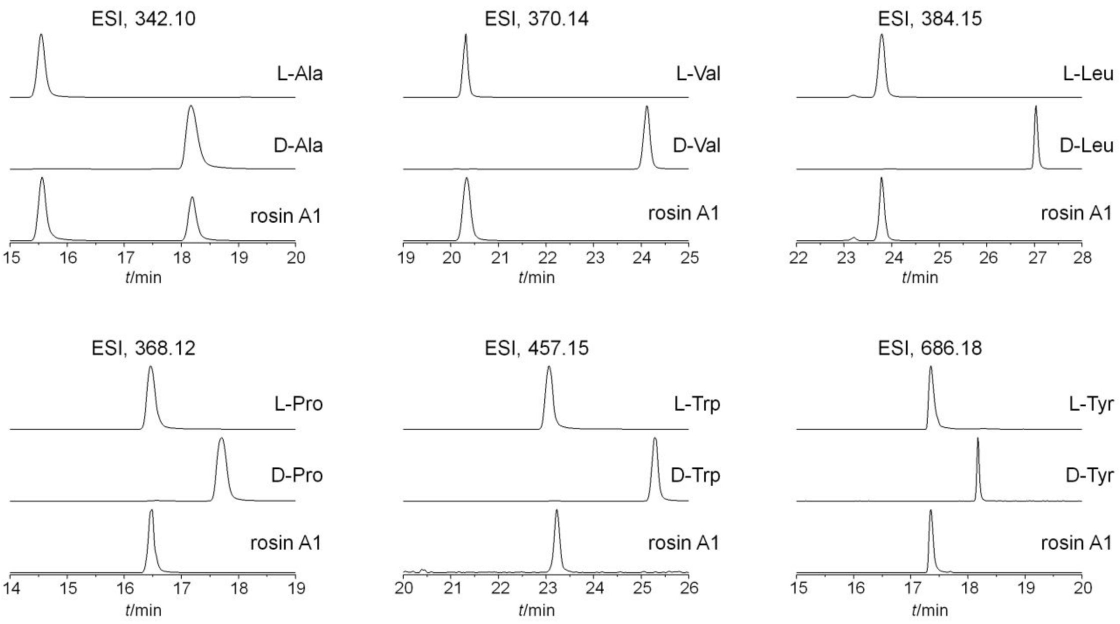

Fig. R8 Marfey's analysis of rosin A1. Tyrosine derivatives are reported as bis-FDAA adducts.

Please see the revised Supporting Information **Page S21, Figure S7** for details.

5. Page 4, line 203: The statement "a *Z*-geometry of the double bond in the AviMeCys crosslink was determined based on the corresponding $^3J_{H,H}$ value of 7.2 Hz" appears to conflict with the NOE correlations shown in Figure 2c. If *Z*-configuration is indeed correct, NOE signals such as those between both NH and Hα of TEE-25 and Hβ of the Abu moiety may be weak or insignificant. Please verify these NOE correlations and indicate whether they are significant.

Response to the comment:

Thank you for your detailed discussion of the *Z*-geometry of this double bond. This is indeed an important point, especially since some references do not report it clearly. In our study, the $^3J_{H,H}$ coupling constant between the two vinylic protons is clearly observed as 7.2 Hz in the 1D ^{1}H NMR spectrum (Figure S8). Additionally, strong NOE signals are observed between the NH and Hα of TEE-25 and the Hβ of the Abu moiety (Figure S17), corresponding to distances of 1.9 Å and 3.7 Å, respectively, in the 3D NMR structural model (2*S*,3*R* configuration for Abu-20) (Fig. R9).

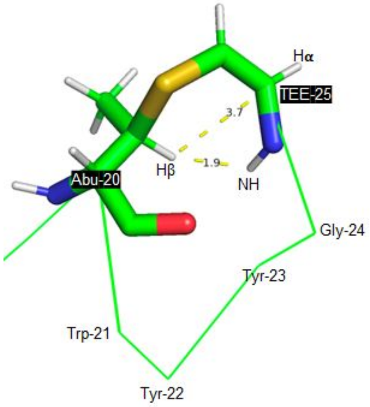

Fig. R9 Distance between the NH and Hα of TEE-25 and the Hβ of the Abu moiety in the 3D NMR structure, consistent with the observed NOE signals.

6. Page 5, line 215-227: The current description of the AviMeCys configuration assignment is difficult to follow. To enhance clarity, consider using Newman projections or alternative schematic representations.

Response to the comment:

Thank you for the helpful suggestion.

The configuration of AviMeCys was determined by analyzing the internal ^{1}H-^{1}H distances within Abu-20, such as HN–Hα, HN–Hγ, HN–Hβ, and Hα–Hγ. Furthermore, the abundant inter-residue NOE signals provide additional support for an unambiguous assignment of the configuration (Figures R10, S12, S17 and S18). Among the possible stereoisomers, the (2*S*,3*R*) configuration best fits the experimental NOE-derived distances. To further enhance clarity, we have added a stereochemical schematic in the revised Supporting Information, highlighting the key ^{1}H-^{1}H distances that support the (2*S*,3*R*) configuration (Figure R10, Figure S18c).

Please see the revised Supporting Information **Page S32, Figure S18c** for details.

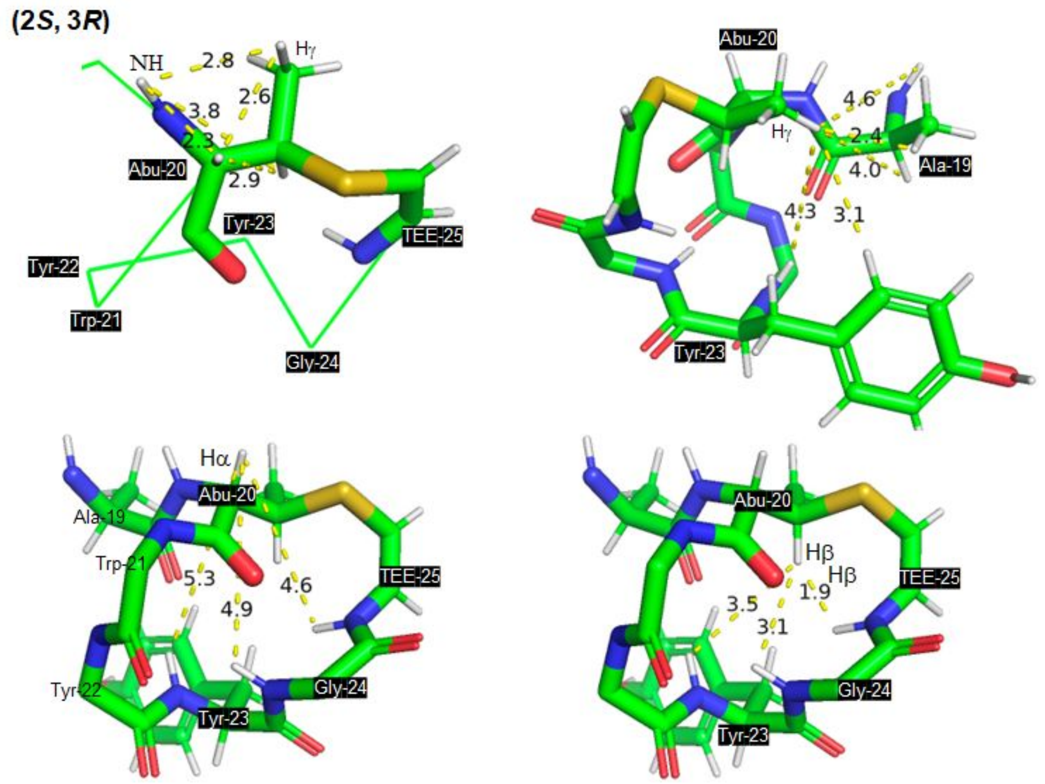

Fig. R10 Schematic representation of the (2*S*,3*R*) configuration of Abu-20, highlighting characteristic intra- and inter-residue ^1H–^1H distances.

Typos are found in the manuscript.

1. Figure 1b, Dha and Dhb should be labeled consistently with their corresponding substructures. Please revise to ensure correct correspondence.

Response to the comment:

This mistake has been corrected. Please see the revised manuscript **Page 2, Figure 1b**.

2. Abbreviations, such as "Abu," are used without being defined at first mention. Please ensure all abbreviations are introduced in full upon first use and consistently applied throughout the manuscript.

Response to the comment:

We have defined the abbreviation at first use in the main text.

Please see the revised manuscript **Page 5, Line 247**.

Reviewer: 3

Recommendation: Major revisions required.

Comments:

In this study Xie et al describe the first in vitro demonstration that a gene annotated originally as a kinase-like protein is involved in the formation of aminovinyl cysteine rings in class Va lanthipeptides. Previous studies by Deng and coworkers had implicated this protein as important based on its need for lexapeptide formation but without directly showing its activity. Another previous study by Liu et al had shown the same for thioviridamide formation and had assigned the function of the kinase-like enzyme as catalyzing the AviCys cyclization together with a decaboxylase (ref 41). The current study verifies these conclusions with in vitro activity and complements those data with several other pieces of data. The study is generally well supported by the data shown in the SI, with some concerns discussed below. If the authors can address these concerns, this study could be appropriate for publication in ACS Cent Sci.

In the abstract (and elsewhere), the wording "their biosynthetic machinery, particularly the cyclases catalyzing Avi(Me)Cys macrocyclization, has remained unknown" is not really correctly describing what is known. Ref 41, which is cited very late in the current paper, provided strong support that the RosX ortholog was directly involved in AviCys formation. In fact, the conclusions in ref 41 closely parallel the conclusions in the current study, with the important distinction that the current study shows direct in vitro evidence whereas ref 41 used co-expression. Hence, I believe that the authors of the current study should more appropriately acknowledge the findings in ref 41 and do so earlier. Yes, that study focused on thioviridamides and not lanthipeptides, but the exact same set of genes are involved. The current presentation of discovery of a new activity is not really describing accurately what was known.

Response to the comment:

We sincerely thank the reviewer for the valuable comments.

We agree with the reviewer that previous studies by Liu group, Tao group, Muller and Koehnke group have provided genetic and biochemical evidence that LanX proteins and their analogs in the thioamitide biosynthesis are essential for AviCys macrocyclization. Liu et al (*Cell Chemical Biology* **2021**, 28, 675–685) proposed that $TvaE_{S-87}$, the LanX homolog, might be the AviCys cyclase or a noncatalytic engaging protein, in which case $TvaF_{S-87}$ possesses dual activities of decarboxylase and AviCys cyclase. Our study is built on these previous studies and provides direct experimental evidence to support LanX as the AviCys cyclase.

In the revised manuscript, we have revised the wording in the abstract and introduction section to accurately reflect this prior knowledge and properly acknowledge these important findings. We also moved the citation of ref 41 (now ref 24) earlier in the text to give it appropriate visibility. We thank the reviewer again for pointing out this important context.

Please see the revised manuscript **Page 1, Line 16-17**; **Page 3, Line 100-115**.

The current work shows that the first ring in rosin A1 can be formed non-enzymatically with some facilitation by RosYK. But the authors do not at all comment on their conclusion that this

spontaneously formed ring has the "DL"-configuration. They cite several other studies where spontaneous cyclization from a similar motif has been reported, but as far as I can tell, those other studies all generated LL stereochemistry. Hence, it seems that the system under study is an exception but the authors do not seem to recognize this or do not want to point out the difference. Please discuss.

Response to the comment:

We sincerely thank the reviewer for this insightful comment.

As mentioned by the reviewer, to date, most characterized Lan crosslinks formed via the (Dha/Dhb)$_2$-Xxx-Xxx-Cys substrate-controlled mechanism in class II and class V$_a$ lanthipeptides are in the LL configuration, with cacaoidin and rosin A1 as the exceptions containing DL-configured Lan crosslinks (Francisco J. Ortiz-López et al., *Angew Chem Int Ed Engl* **2020**, 59, 12654-12658). This might imply the involvement of additional stereochemical mechanisms during non-enzymatic Lan cyclization, which requires further investigation.

We have included this discussion in the revised manuscript. Please see the revised manuscript **Page 7, Line 417-423**.

The authors report they used advanced Marfey analysis using 1-fluoro-2,4-dinitrophenyl-5-L/D-alanine amide (L/D-FDAA) which is a confusing way of wording. Researchers usually use either L-FDAA or D-FDAA, not a mixture. In fact in Fig S7 it seems they only used L-FDAA. The other confusing thing is that advanced Marfey analysis refers to the use of the Leu derivative (L-FDLA), not FDAA. The authors seem to use the regular Marfey's method and not the advanced method.

Response to the comment:

Thanks for the careful evaluation and pointing out this inconsistency.

We have revised the manuscript accordingly:

"To analyze the amino acid components of rosin A1, we conducted advanced Marfey's analysis for rosinA1 using 1-fluoro-2,4-dinitrophenyl-5-L/D-alanine amide (L/D-FDAA)" is now modified to "To analyze the amino acid components of rosin A1, we conducted Marfey's analysis for rosinA1 using 1-fluoro-2,4-dinitrophenyl-5-L-alanine amide (L-FDAA)".

Please see the revised manuscript **Page 4, Line 208-210**.

On the topic of stereochemistry, the authors always draw the enethiol as having E stereochemistry in several figures. What is the support for this? If indeed *E*, how is the stereochemistry changed to *Z* in the cyclized product?

Response to the comment:

Thanks for the comment.

As discussed in the response to Reviewer #1 Comment #2. The geometry of enethiol (RosD product) is generally proposed to adopt a cis-configuration after Cys decarboxylation (Clarissa

S. Sit et al., *Acc. Chem. Res.* **2011**, 44, 261-268; Emily S. Grant-Mackie et al., *JACS Au* **2021**, 1, 1527-1540; Botao Cheng et al., *ChemPlusChem* **2024**, e202400047). Theoretical analysis of Avi(Me)Cys derivatives supports that the cis-configuration is thermodynamically favorable (Angela K. Carrillo et al., *Org. Lett.* **2017**, 19, 5146-5149).

In the original manuscript, we made mistakes by drawing the enethiol motif in *trans* configuration in Fig. 4a and Fig. S33. We have corrected these mistakes accordingly.

Please see the revised manuscript **Page 8, Figure 4a**, and the revised Supporting Information **Page S55, Figure S33** for details.

One of the more unique aspects of this report is the demonstration of promotion of wound healing. Since this is highly likely to be a non-physiological activity, the authors should test if the unmodified core peptide (or any of the analogs they made) has the same activity or whether the modifications are required.

Response to the comment:

Thanks for the comment.

As suggested by the reviewer, we have performed parallel experiments with the unmodified RosA1 core peptide for comparison. Results showed that the RosA1 core peptide displayed no cell migration promotion effect (Figure R11), indicating that the modification of rosin A1 is required for its bioactivity. We have provided this information in the revised manuscript.

Please see the revised manuscript **Page 5, Lines 286-289**, Supporting Information **Page S35, Figure S20d-f** for details.

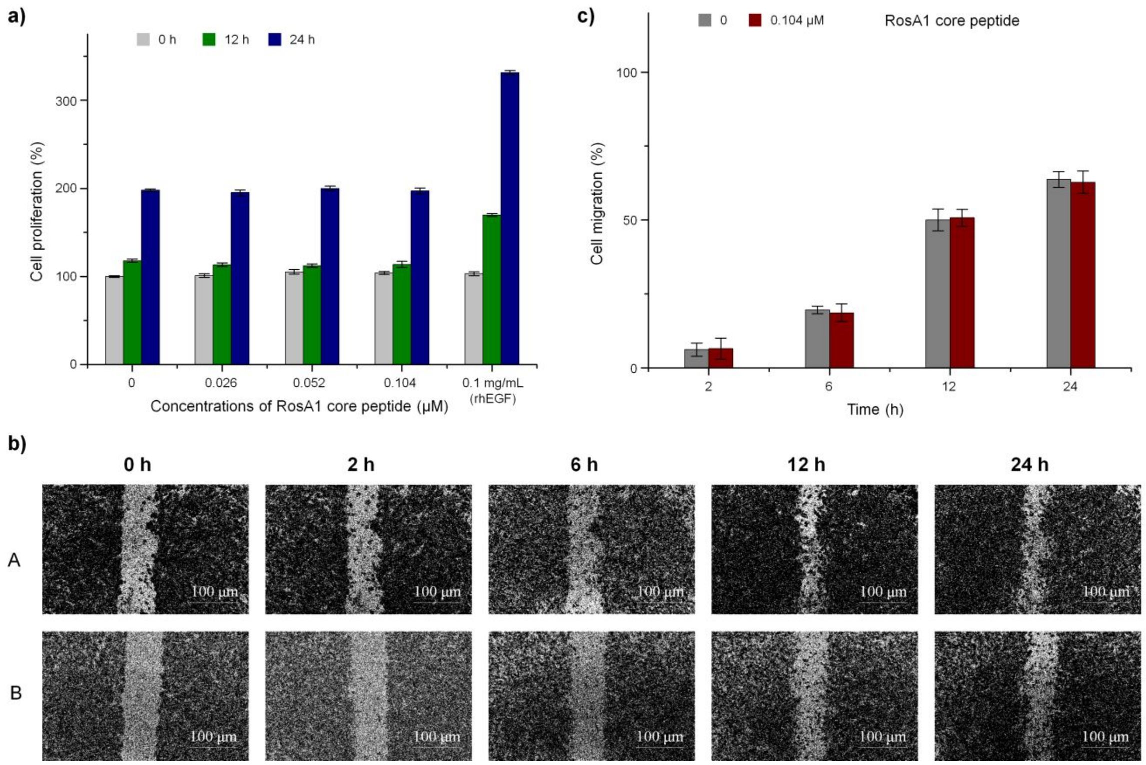

Fig. R11 (a). Proliferation trend of HSF induced by RosA1 core peptide. rhEGF was used as a positive control. (b). Wound-healing assay of HSF induced by RosA1 core peptide. (A. blank, B. 0.104 μM RosA1 core peptide). (c). Migration of HSF induced by RosA1 core peptide.

The authors mention that their trimer of RosK and RosY is different from other studies on homologous proteins. They should make an AlphaFold3 model of this trimer and compare its pLDDT values with the dimer. Also, the authors mention that RosK forms a dimer but they do not say whether RosY by itself is a monomer or dimer. I am not convinced that SEC data with just one standard (SpaC-SpaD) and without a standard curve with a series of standards is sufficient support for the proposed 2:1 stoichiometry. AlphaFold and a standard curve may be able to provide stronger support.

Response to the comment:

We thank the reviewer for this insightful comment.

In revised manuscript, we have added additional SEC data to Fig. 3a and Fig. S22, including a calibration curve generated using a series of molecular weight standards. The calibration confirms that RosY alone exists as a monomer, and the RosK–RosY complex has a molecular weight consistent with a 2:1 stoichiometry, distinct from the SpaC–SpaD complex. Please see the revised manuscript **Page 6, Fig. 3a**, and the revised Supporting Information **Page S37, Figure S22a** for details.

We also followed the reviewer's suggestion to model the RosK–RosY dimer (1:1) and trimer (2:1) complexes using AlphaFold3 (Fig. R12). Both predicted models yielded ipTM scores in the range of 0.6 to 0.8, which falls into the uncertain confidence zone and does not allow a definitive structural conclusion. We are trying to using structural biology techniques to

solve the structure of the RosK-RosY complex, and hopefully to report new findings in the near future.

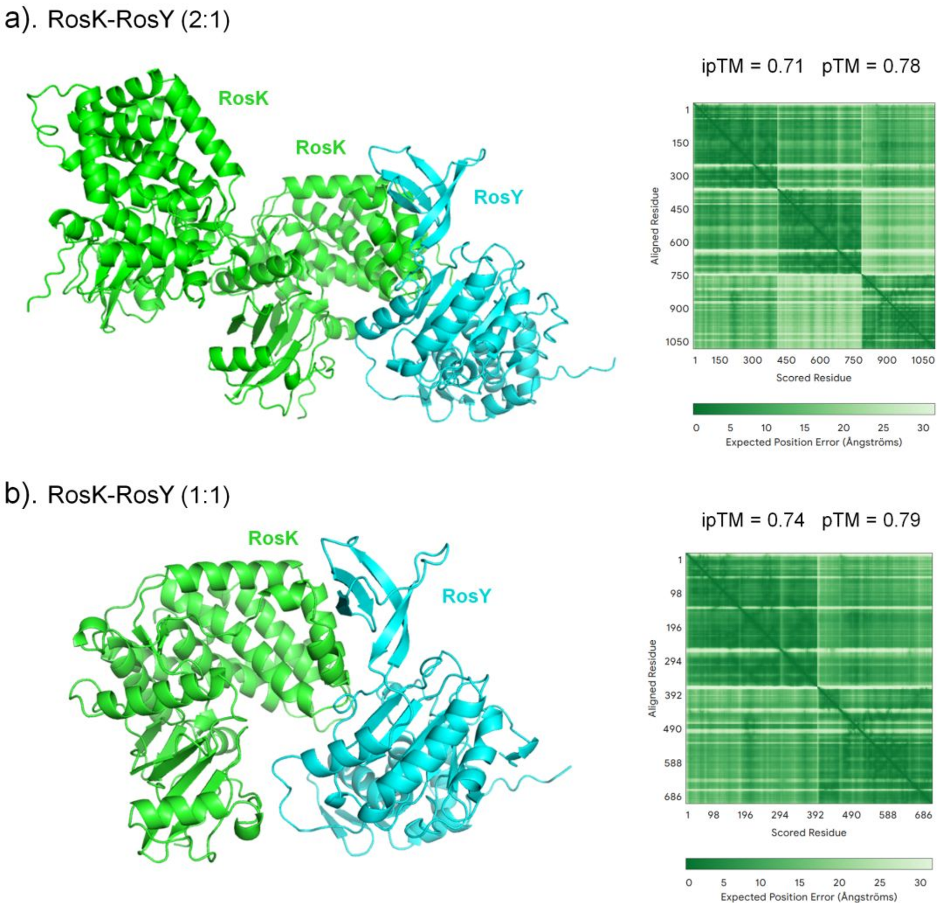

Fig. R12 Predicted complex structures of RosK–RosY (2:1 and 1:1) and their confidence scores.

Regarding using AlphaFold, what is the predicted structure of RosA1 binding to RosY-RosK? While the prediction of alpha helicity for the substrate by itself looks well supported, the peptide could bind very differently to the enzyme.

Response to the comment:

Thanks for the insightful comment.

Following the reviewer's suggestion, we have used AlphaFold3 to predict the complex structure of RosK–RosY (2:1) bound to the precursor peptide RosA1. The predicted pTM and ipTM scores of the RosK–RosY–RosA1 complex are both higher than those of the RosK–RosY (2:1) complex alone, suggesting that substrate binding may contribute to the overall stability of the complex. The predicted binding mode of the RosA1 leader peptide (RosA1$_{LP}$) shows high confidence (pLDDT > 90) and retains an α-helical structure (Fig. R13), which is consistent with the structure predicted for the leader peptide alone. This indicates that the interaction with the enzyme complex might not significantly alter the inherent secondary structure of the leader peptide.

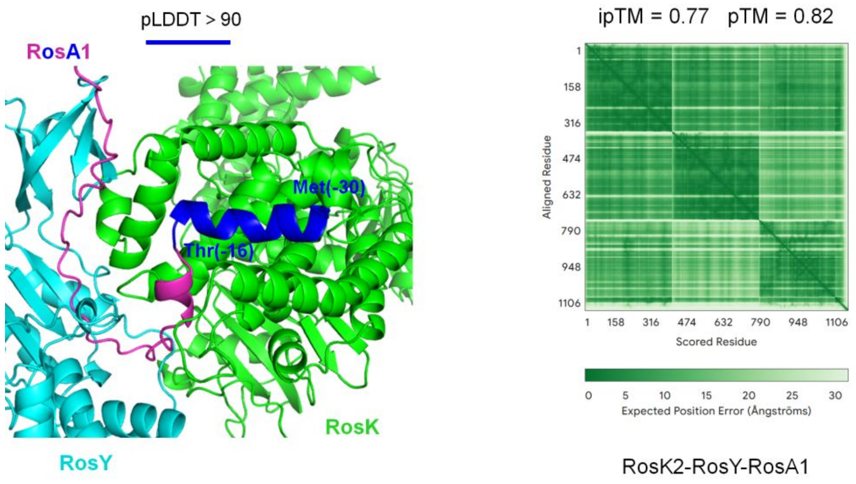

Fig. R13 Predicted complex structures of RosK–RosY-RosA1 and its confidence scores.

All of the following statements are missing citations or the citations are incomplete:

"Unlike other reported class V lanthipeptide BGCs, the ros cluster does not contain a methyltransferase."

Response to the comment:

Thanks for pointing this out. We have added the appropriate references to support the statement in the revised manuscript. Please see the revised manuscript **Page 3, Line 148-149** for details.

"This is distinct from their homologs CaoK-CaoY from cacaoidin biosynthesis and SpaC-SpaD from thiosparsoamide biosynthesis, which form heterodimer in solution."

Response to the comment:

Appropriate references and the corresponding figures have been added in the revised manuscript. Please see the revised manuscript **Page 5, Line 307-308** for details.

"MSMS analysis also revealed the presence of an N-terminal 2-oxobutyryl group (Obu), which is likely generated from the spontaneous deamination of an N-terminal Dhb residue after the leader removal (Fig. S6b).23" This was first shown in Kellner, R.; Jung, G.; Josten, M.; Kaletta, C.; Entian, K. D.; Sahl, H. G. Angew. Chem. 1989, 101, 618 and should be cited.

Response to the comment:

Thanks for pointing this out. We have now cited the original work. Please see **Page 4, Line 207, Ref. 28.**

"LanX has been proposed to be essential for the Avi(Me)Cys biosynthesis in class Va lanthipeptide and thioamitides."

Response to the comment:

We have added the appropriate references. Please see **Page 7, Line 443, Ref. 22, 24, 25**.

"Consistent with typical LanD proteins, RosD forms a dodecamer in vitro, as determined by SEC analysis (Fig. S33a).33" While ref 33 by the authors indeed also shows this, this observation was already made much earlier in EMBO J . 2000 Dec 1;19(23):6299-310.

Response to the comment:

Thanks for pointing this out. We have now cited the original work. Please see **Page 7, Line 436, Ref. 39.**

I did not understand the citation of ref 29 in this sentence: "Although the Ala6-Cys10 Lan crosslink (Lan6-10) was identical to that in the authentic rosin A1, the C-terminal Ala15-Cys25 Lan crosslink was formed with distinct regioselectivity compared with the AviMeCys crosslink in rosin A1.29" I read ref 29 and did not understand its relevance to this sentence. Please expand text.

Response to the comment:

Thanks for the comment. We have removed the original ref 29 to this sentence.

Fig. S22, panel c. This is an odd-looking maximum likelihood phylogenetic plot. What are the distances/bootstrap values? How was it generated? I could not find it in the Methods or legend.

Response to the comment:

Thanks for the comment.

We have revised Fig. S22c by reconstructing the phylogenetic tree and now show bootstrap values greater than 70 to indicate branch support. The corresponding construction method has also been added to the *Supplemental Materials and Methods* section (page S3, Line 66-71). Although the branch support between RosY and CaoY is relatively low, RosK appears evolutionarily distant from SpaC and CaoK, and RosY from SpaD. This evolutionary divergence may partially account for the differences in oligomerization patterns observed among these proteins. Nevertheless, we emphasize that the precise determinants of oligomerization remain to be further investigated.

Please see the revised Supporting Information **Page S3, Line 66-71** and **Page37, Figure S22c** for details.

Typo's:

Figure legend Fig 1 has text repeated (bold text)

Response to the comment:

This mistake has been corrected. Please see the revised manuscript **Page 2, Line 74**.

Page 3: 87 sturctures:

Response to the comment:

This mistake has been corrected. Please see the revised manuscript **Page 3, Line 92**.

Bacillus subtills

Response to the comment:

This mistake has been corrected. Please see the revised manuscript **Page 5, Line 279**.

Page 5, line 280 There results should be These results.

Response to the comment:

This mistake has been corrected. Please see the revised manuscript **Page 5, Line 324.**

Fig 3 legend ration should be ratio

Response to the comment:

Thank you for pointing this out. We have replaced the legend of Fig. 3 in the revised manuscript, and the word “ration” no longer appears. Please see the revised manuscript **Page 6, Line 375**.

oc-2025-00569x.R2

Name: Peer Review Information for "A Unique Class of Cyclases with a Kinase Fold Catalyzes Enethiol-mediated Macrocyclization of Aminovinyl-Cysteine Motifs in Lanthipeptides"

Second Round of Reviewer Comments

Reviewer: 1

Comments to the Author

The authors have responded to reviewers' concerns and the manuscript is now acceptable for publication.

Reviewer: 3

Comments to the Author

Generally the authors have addressed my concerns adequately. I am still worried about the stereochemistry of the spontaneously generated ring. In the response letter and in the manuscript the authors say that their finding has precedent for cacaidin, but I don’t think that is correct. They cite ref 21 as showing this, but that study did not experimentally determine the stereochemistry in an unambiguous way (no standards of defined configuration were used and stereochemistry was inferred). A more recent paper (ref 19) showed that the stereochemistry of that ring is most likely LL like all other cases. So, rosin would be the first example and I am worried that it is incorrect. At the very least the authors need to say that this is the only example (I strongly recommend they make absolutely sure they are correct) and remove the statement that cacoidin also has that stereochemistry as it was not truly shown and saying it will continue proliferating something without true support.

Reviewer: 2

Comments to the Author

The manuscript has been improved significantly by following the reviewer's comments and thus the current version is satifactory. The reviewer suggests that this should be published without change.

Author's Response to Peer Review Comments:

Response Letter

Formatting Needs:

1. Highlighting: Please submit your publication files without any markups. Any copies that contain highlights, colored text, or tracked changes should be submitted as "Supporting Information for Review Only."

Response to the comment:

Thanks for pointing this out. In accordance with the journal's guidelines, we have now submitted a clean version of the manuscript without any markups, highlights, or tracked changes.

The marked-up copy has been moved to the "Supporting Information for Review Only" section to ensure clarity for the editorial process.

Reviewer: 3

Recommendation: Publish in ACS Central Science after minor revisions noted.

Comments:

Generally the authors have addressed my concerns adequately. I am still worried about the stereochemistry of the spontaneously generated ring. In the response letter and in the manuscript the authors say that their finding has precedent for cacaidin, but I don't think that is correct. They cite ref 21 as showing this, but that study did not experimentally determine the stereochemistry in an unambiguous way (no standards of defined configuration were used and stereochemistry was inferred). A more recent paper (ref 19) showed that the stereochemistry of that ring is most likely LL like all other cases. So, rosin would be the first example and I am worried that it is incorrect. At the very least the authors need to say that this is the only example (I strongly recommend they make absolutely sure they are correct) and remove the statement that cacoidin also

has that stereochemistry as it was not truly shown and saying it will continue proliferating something without true support.

Response to the comment:

We sincerely thank the reviewer for the valuable comments.

We acknowledge the valid concern regarding the precedent citation of cacaoidin's stereochemistry and agree that Ref 21 did not provide definitive experimental evidence. Ref 19 provides a very comprehensive structural characterization method to determine the configuration of the MeLan ring in triantimycins, and we have learned and followed the method developed in Ref. 19.

In our study, the configuration of the $Lan_{6\text{-}10}$ ring in rosin A1 was determined through Marfey's derivatization and NMR characterization. Both methods yielding results that are consistent with the DL-configuration.

Following the reviewer's request, we have revised the manuscript to remove comparisons with cacaoidin, and now explicitly state that this may represent a rare stereochemical DLconfiguration. We are particularly grateful for the reviewer's comments that have enriched the discussion about structural characterization standards in RiPPs research. In our ongoing work, we are actively pursuing crystallographic verification to further substantiate these findings.

Please see Page 7, Line 417-421 in the revised manuscript:

"Although Lan/MeLan crosslinks in class V_a lanthipeptides are predominantly LL-configured due to their substrate-controlled mechanism, the Lan6-10 crosslink in rosin A1 exhibit the rare DL-configuration."
